# Deficiency of parkin causes neurodegeneration and accumulation of pathological **α**-synuclein in monkey models

**DOI:** 10.1172/JCI179633

**Published:** 2024-10-15

**Authors:** Rui Han, Qi Wang, Xin Xiong, Xiusheng Chen, Zhuchi Tu, Bang Li, Fei Zhang, Chunyu Chen, Mingtian Pan, Ting Xu, Laiqiang Chen, Zhifu Wang, Yanting Liu, Dajian He, Xiangyu Guo, Feng He, Peng Wu, Peng Yin, Yunbo Liu, Xiaoxin Yan, Shihua Li, Xiao-Jiang Li, Weili Yang

**Affiliations:** 1 State Key Laboratory of Bioactive Molecules and Druggability Assessment, Guangdong Key Laboratory of Non-human Primate Research, GHM Institute of CNS Regeneration, Jinan University, Guangzhou, China.; 2 Hubei Topgene Biotechnological Research Institute Co., Ltd. Wuhan, China.; 3 Institute of Laboratory Animal Science, Chinese Academy of Medical Sciences and Peking Union Medical College, Beijing, China.; 4 Department of Anatomy and Neurobiology, Xiangya School of Medicine, Central South University, Changsha, China.

**Keywords:** Aging, Neuroscience, Parkinson disease

## Abstract

Parkinson’s disease (PD) is characterized by age-dependent neurodegeneration and the accumulation of toxic phosphorylated α-synuclein (pS129-α-syn). The mechanisms underlying these crucial pathological changes remain unclear. Mutations in parkin RBR E3 ubiquitin protein ligase (*PARK2*), the gene encoding parkin that is phosphorylated by PTEN-induced putative kinase 1 (PINK1) to participate in mitophagy, cause early onset PD. However, current parkin-KO mouse and pig models do not exhibit neurodegeneration. In the current study, we utilized CRISPR/Cas9 technology to establish parkin-deficient monkey models at different ages. We found that parkin deficiency leads to substantia nigra neurodegeneration in adult monkey brains and that parkin phosphorylation decreases with aging, primarily due to increased insolubility of parkin. Phosphorylated parkin is important for neuroprotection and the reduction of pS129-α-syn. Consistently, overexpression of WT parkin, but not a mutant form that cannot be phosphorylated by PINK1, reduced the accumulation of pS129-α-syn. These findings identify parkin phosphorylation as a key factor in PD pathogenesis and suggest it as a promising target for therapeutic interventions.

## Abstract 2

<strong>Primate</strong><strong>-</strong><strong>specific </strong><strong>phosphorylation</strong><strong> of </strong><strong>Parkin </strong><strong>contributes to</strong><strong> the substantia nigra</strong><strong> neurodegeneration in</strong> <strong>Parkinson’s disease </strong>

## Graphical abstract

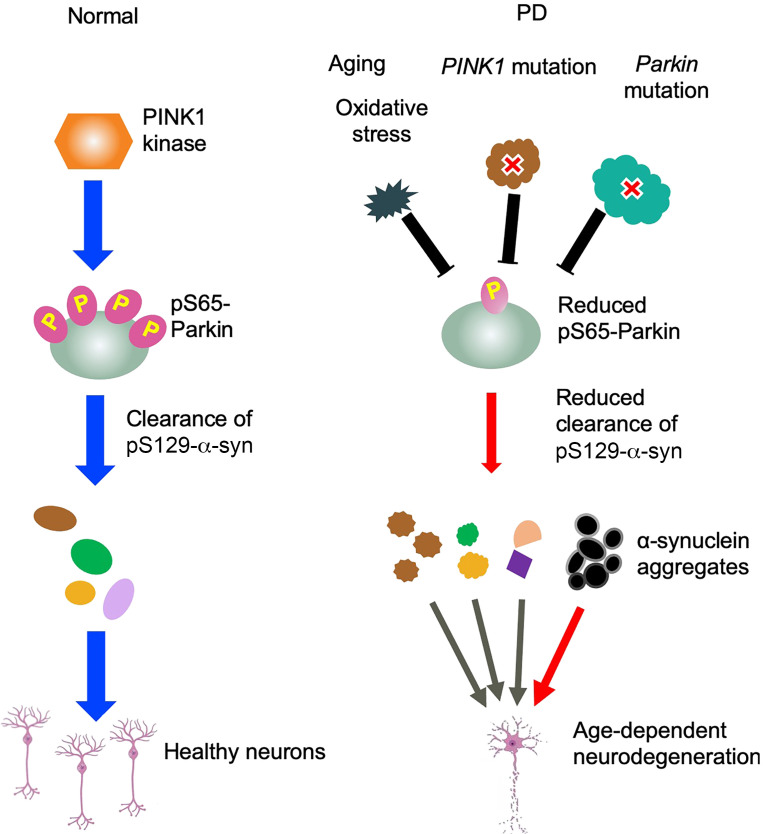

## Introduction

Parkinson’s disease (PD) is a prevalent neurodegenerative disease that affects 1%–2% of individuals over the age of 65 years (1). Although the majority of PD cases are sporadic with unknown causes, the common pathological event in PD is the age-dependent degeneration of substantia nigra (SN) neurons, which produce dopamine for striatal neurons to maintain normal body movement (1). Another key pathological feature in PD cases is the age-dependent formation of Lewy neurites or bodies caused by the accumulation of toxic α-synuclein (α-syn) or its phosphorylated form (pS129-α-syn), both of which are detrimental to neurons (2). However, the mechanisms underlying age-dependent neurodegeneration and accumulation of pS129-α-syn remain unclear.

Mutations in several genes, including PTEN-induced putative kinase 1 (*PINK1*) and the parkin gene parkin RBR E3 ubiquitin protein ligase (*PARK2*), cause autosomal recessive early onset PD in association with the accumulation of misfolded proteins (3). Numerous biochemical and in vitro studies have provided compelling evidence that PINK1 phosphorylates and activates parkin, an E3 ubiquitin ligase (4–8). Parkin phosphorylation leads to the removal of unhealthy mitochondria through the autophagic machinery. This process, known as mitophagy, is believed to protect neuronal cells (9, 10). However, all these findings were obtained from in vitro studies or under mitochondrial stress conditions. In vivo studies have been unable to demonstrate that the absence of PINK1 or parkin can alter the basal activity of mitophagy (11, 12). To date, there is a lack of strong in vivo evidence to demonstrate that endogenous PINK1 can phosphorylate parkin under physiological conditions. Nevertheless, a mutation (Ser65Ala) affecting parkin phosphorylation has been shown to cause PD (13).

Animal models play a crucial role in investigating the in vivo role of PD pathogenesis. However, mouse models with genetic mutations that can cause autosomal recessive PD have failed to show neurodegeneration, noticeable Lewy bodies, or the accumulation of pS129-α-syn observed in PD patients (14–18). Knocking out *PINK1* and *PARK2* in pigs via CRISPR/Cas9 was not found to elicit neurodegeneration either (19, 20). Histological studies of postmortem brains from patients with *PINK1* or *PARK2* mutations have yielded inconsistent and ambiguous results regarding the presence of Lewy bodies (21–28).

Our recent studies have revealed that PINK1 deficiency can lead to neurodegeneration in the developing brains of monkeys, primarily due to the loss of its kinase activity (29, 30). These findings have raised several important questions that need further investigation. First, we need to determine whether parkin deficiency in primate brains produces phenotypes similar to those with PINK1 deficiency because parkin is a substrate of PINK1. Second, we need to understand how mutations in *PINK1* and *PARK2* can result in similar phenotypes in humans. Finally, we need to explore whether the deficiency of parkin activity is associated with the accumulation of toxic α-syn, a pathological hallmark in PD.

Given that parkin is activated through PINK1-mediated phosphorylation (4, 31–34), and considering that PINK1 is uniquely expressed in primate brains (30, 35), it is essential to use nonhuman primate models to address the aforementioned questions. In our current study, we have discovered that parkin deficiency in monkey brains leads to age-dependent neurodegeneration in the SN, a phenomenon distinct from the degeneration observed in the developing brains in *PINK1* targeted monkeys. Consistently, parkin expression differs from that of PINK1. However, its phosphorylation at S65 (pS65-parkin) is dependent on PINK1. As the monkeys age, parkin phosphorylation decreases, resulting in increased insolubility and the accumulation of pS129-α-syn. Furthermore, overexpression of parkin, but not the nonphosphorylated form of parkin, reduces the age-dependent accumulation of pS129-α-syn. Therefore, our findings highlight the critical role of parkin phosphorylation deficiency in age-dependent neurodegeneration.

## Results

### Targeting PARK2 in monkey caused age-dependent neurodegeneration.

**Figure 1:**
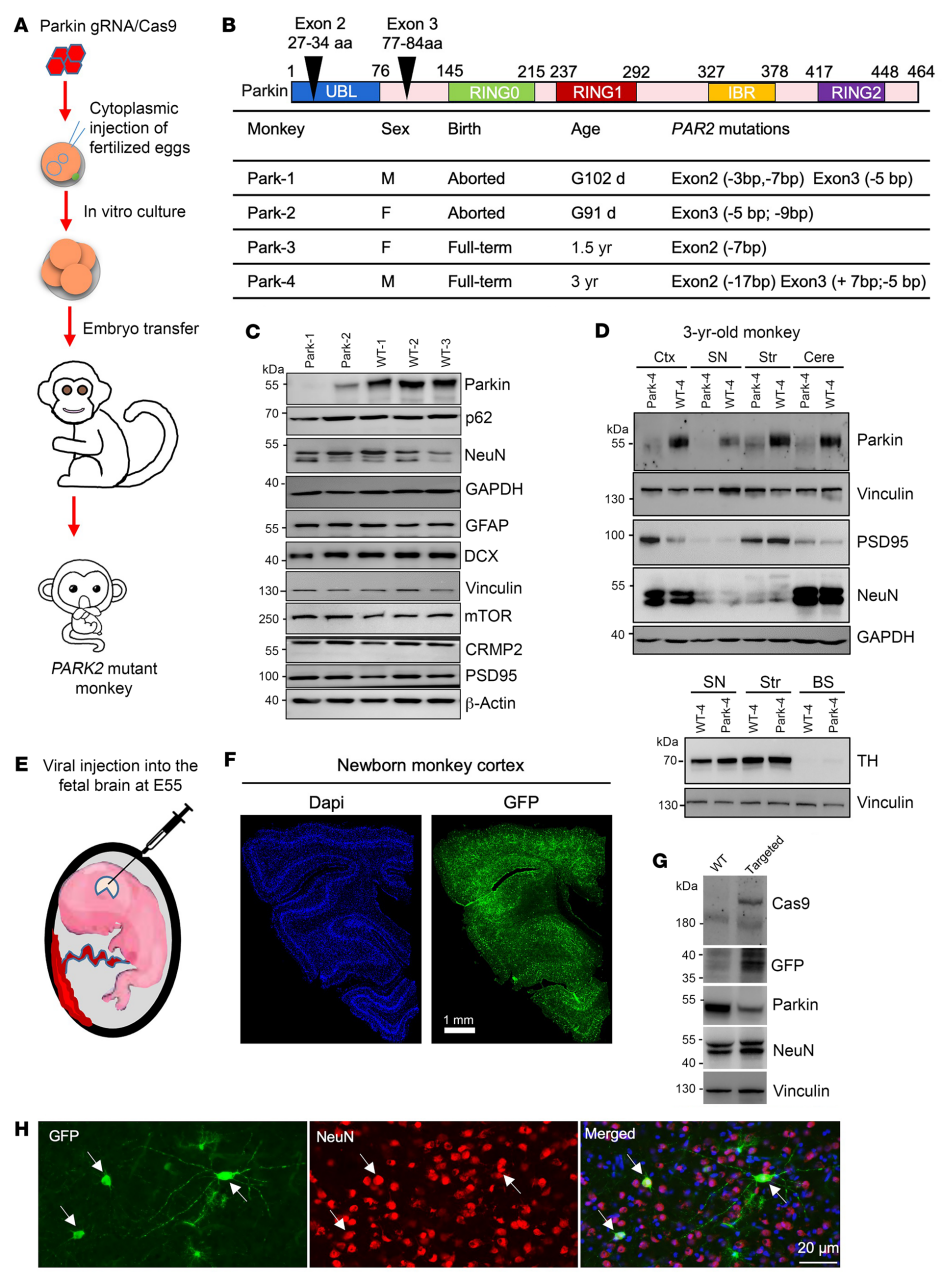
Parkin mutations do not induce neuronal loss in the developing monkey brain. (**A**) Schematic diagram illustrating the establishment of an embryonic *PARK2* targeting monkey model. One-cell stage embryos of Macaque monkeys were injected with parkin gRNA and Cas9 mRNA. Embryos at the 4–8 cell stage were transferred to surrogates to produce newborn monkeys with parkin mutations. (**B**) Parkin protein domains and targeted regions. Two parkin gRNAs (exon 2 and exon 3) were designed to target the monkey parkin (*PARK2*) gene. Two fetal monkeys (Park-1 and Park-2), one 1.5-year-old monkey (Park-3), and one 3-year-old monkey (Park-4) were found to carry parkin mutations generated by CRISPR/Cas9 targeting. (**C** and **D**) Western blotting showing a reduction in parkin levels in the brain tissues of Park-1 and Park-2 monkeys in **C**, as well as 3-year-old Park-4 monkey in **D**, without alterations in neuronal proteins (NeuN, PSD95) when compared with age-matched WT monkeys. Representative Western blotting results of multiple technical replicates from 3 WT and 3 parkin mutant monkeys. Ctx, cortex; Str, striatum; Cere, cerebellum; BS, brain stem. (**E**) In utero delivery of lentiviral parkin gRNA/Cas9 into the lateral ventricle of the fetal monkey brain at approximately G55. (**F**) Widespread expression of GFP, reflecting lentiviral parkin gRNA/Cas9 transduction in the brain of the newborn monkey. Scale bar: 1 mm. (**G**) Representative Western blotting results of 3 independent experiments showing reduced expression of parkin in the lentiviral-infected brain regions. Loss of parkin did not affect NeuN expression. (**H**) Representative images of NeuN labeling of the cortical region in the parkin-targeted newborn monkey, showing that targeting parkin did not affect NeuN expression or neuronal morphology when compared with untargeted cells. Multiple technical replicates were performed.

We generated *PARK2* mutant monkeys by targeting the *PARK2* gene in fertilized eggs using methods similar to those described in our previous studies (29) (Figure 1A). We designed 2 guide RNAs (gRNAs) to disrupt exons 2 and 3 of the monkey *PARK2* gene (Figure 1B) to mimic the deletion of large *PARK2* DNA fragments in patients (36, 37). *PARK2* gRNAs were coinjected with Cas9 RNA into the fertilized eggs of rhesus monkeys. We transferred 31 embryos to 12 surrogate female monkeys and obtained 2 aborted fetuses at gestational day 102 (G102) (Park-1) and G91 (Park-2) and 2 *PARK2*-targeted monkeys (Park-3 and -4) that were born healthy and developed normally. Park-3, at 1.5 years of age, died suddenly of unknown causes. Park-4 was euthanized at 3 years of age to examine its neuropathology (Figure 1B). T7E1 DNA analysis (Supplemental Figure 1, A and B; supplemental material available online with this article; https://doi.org/10.1172/JCI179633DS1) and sequencing (Supplemental Figure 1, C and D) of the brain DNAs from dead monkeys validated mutations in the targeted *Parkin* exon 2, exon 3, or both.

Western blotting results revealed depletion of parkin to various extents in the fetal brains of *PARK2*-targeted monkeys (Park-1 and Park-2) (Figure 1C), consistent with the nature of mosaic targeting by CRISPR/Cas9. However, no significant alterations of marker proteins for neurons (NeuN, PSD95, CRMP2), astrocytes (GFAP), autophagy (p62), and cell growth or differentiation (mTOR or DCX) were seen, even in the Park-1 brain in which parkin was almost completely depleted (Figure 1C). Due to the limited size of the SN region, the majority of SN tissues were allocated for Western blotting analysis, with a portion reserved for immunostaining. Western blotting analysis revealed that the levels of neuronal proteins (NeuN, PSD95, NFL, TH) in the SN of the Park-4 monkey did not differ from those in the WT monkey (Figure 1D). Immunostaining of NeuN-positive cells also showed no difference between WT and Park-4 monkey brain regions (Supplemental Figure 2). This finding was further supported by Western blotting analysis, showing no significant loss of neuronal and mitochondrial proteins in the cortex, striatum, and SN of the Park-4 monkey compared with the WT monkey (Supplemental Figures 3 and 4).

The lack of obvious neurodegeneration in the developing *parkin*-targeted monkey brain is different from the noticeable neuronal loss in the developing brain cortex of *PINK1*-targeted monkeys (30) but is in agreement with the fact that the deletion of the *PARK2* gene does not affect early human development (36). To validate that *PARK2* mutations do not trigger neuronal loss in the developing monkey brain, we conducted in utero injection of lentivirus (LV) expressing *PARK2* gRNA (parkin gRNA) GFP/Cas9 into the fetal monkey brain at G55 (Figure 1E), following the method established in our recent study (38). At G55, the lateral ventricle is visible for in utero injection under ultrasound guidance. A newborn monkey was naturally delivered and analyzed at P7. Since LV was used to express parkin gRNA and GFP, GFP-positive cells represent those cells that were targeted by parkin gRNA/Cas9. The in utero LV injection resulted in widespread expression of the GFP transgene in the newborn monkey brain cortex (Figure 1F). However, we did not observe strong evidence of efficient viral infection in the SN. Therefore, our focus remained on investigating the effects of parkin knockdown (KD) in the cortex to confirm the absence of noticeable neuronal loss, with a comparison with the previously identified effects of PINK1 KD. Western blotting showed the expression of Cas9 and GFP with a reduced level of parkin. However, NeuN expression was not decreased (Figure 1G), which was also confirmed by double immunofluorescent staining showing that GFP-positive cells displayed normal neuronal processes and levels of NeuN similar to those of untargeted cells (Figure 1H). Together, these data suggest that *PARK2* mutations do not induce neurodegeneration in the developing monkey brain, in contrast to our previous finding that knocking down PINK1 can result in severe neuronal loss in the brain cortex during early monkey brain development (29, 30).

**Figure 2:**
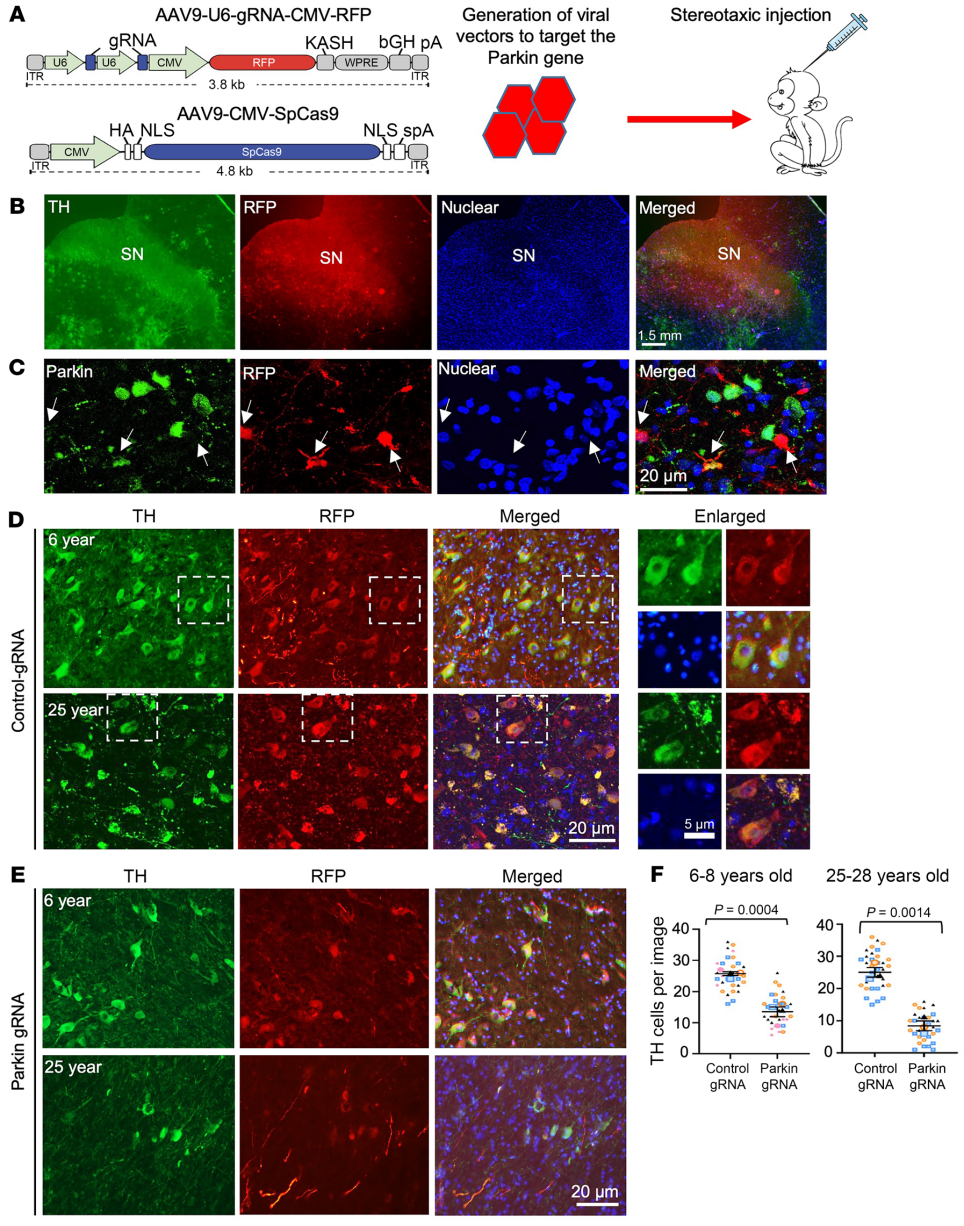
Targeting parkin in the adult monkey brain leads to SN neurodegeneration. (**A**) The monkey parkin gene (*PARK2*) was targeted by stereotaxic injection of AAV9-parkin gRNA-RFP/Cas9 into the monkey brain region. A control group was injected with AAV-Cas9 along with control gRNA-RFP. (**B**) Low-magnification micrographs show viral infection reflected by RFP in the injected monkey SN. Scale bar: 1.5 mm. (**C**) Double immunofluorescent staining indicates that AAV9-parkin gRNA-RFP/Cas9-infected neurons (arrows) in the SN displayed reduced expression of parkin. (**D** and **E**) Representative images of immunofluorescent staining revealed that targeting parkin in the SN of 6- or 25-year-old monkeys (**E**) resulted in a reduction of TH-positive neurons as compared with AAV-control gRNA-injected SN (**D**). Enlarged images in white boxed areas in the control gRNA groups are also presented to show viral infection of neuronal cells. Scale bar: 5 μm. Representative immunofluorescent staining images in **B**–**E** are from at least 3 biological replicates with multiple technical replicates. (**F**) Superplot analysis of the number of TH-positive neurons in the SN of monkeys injected with AAV-Cas9/control-gRNA and AAV-Cas9/parkin-gRNA. The experimental groups included 4 young monkeys aged 6–8 years and 3 old monkeys aged 25–28 years. The number of TH-positive cells in each image (×20) was recorded and color coded to represent the specific monkey brain it originated from. The average count of TH-positive cells in each animal was used for paired 2-tailed *t* test to obtain the *P* values. Data are represented as means ± SEM (*n* = 4 for 6-8 years and *n* = 3 for 25–28 years).

The extended period (4–5 years) of sexual maturity in monkeys and the high costs associated with nonhuman primate research limit extensive investigations of age-dependent pathological changes in monkeys with genetic editing at the fertilization stage. To determine whether parkin deficiency can elicit age-dependent neurodegeneration, we used stereotaxic injection of AAV9 to express parkin gRNA and Cas9 in the cortex and SN of adult monkeys at young ages (6–8 years) or old age (25–28 years) (Figure 2A and Supplemental Table 1). For comparison, the control gRNA was injected into the contralateral side of the cortex and SN in the same animal. The injected monkey brains were isolated for examination 11–12 weeks after AAV injection.

**Figure 3:**
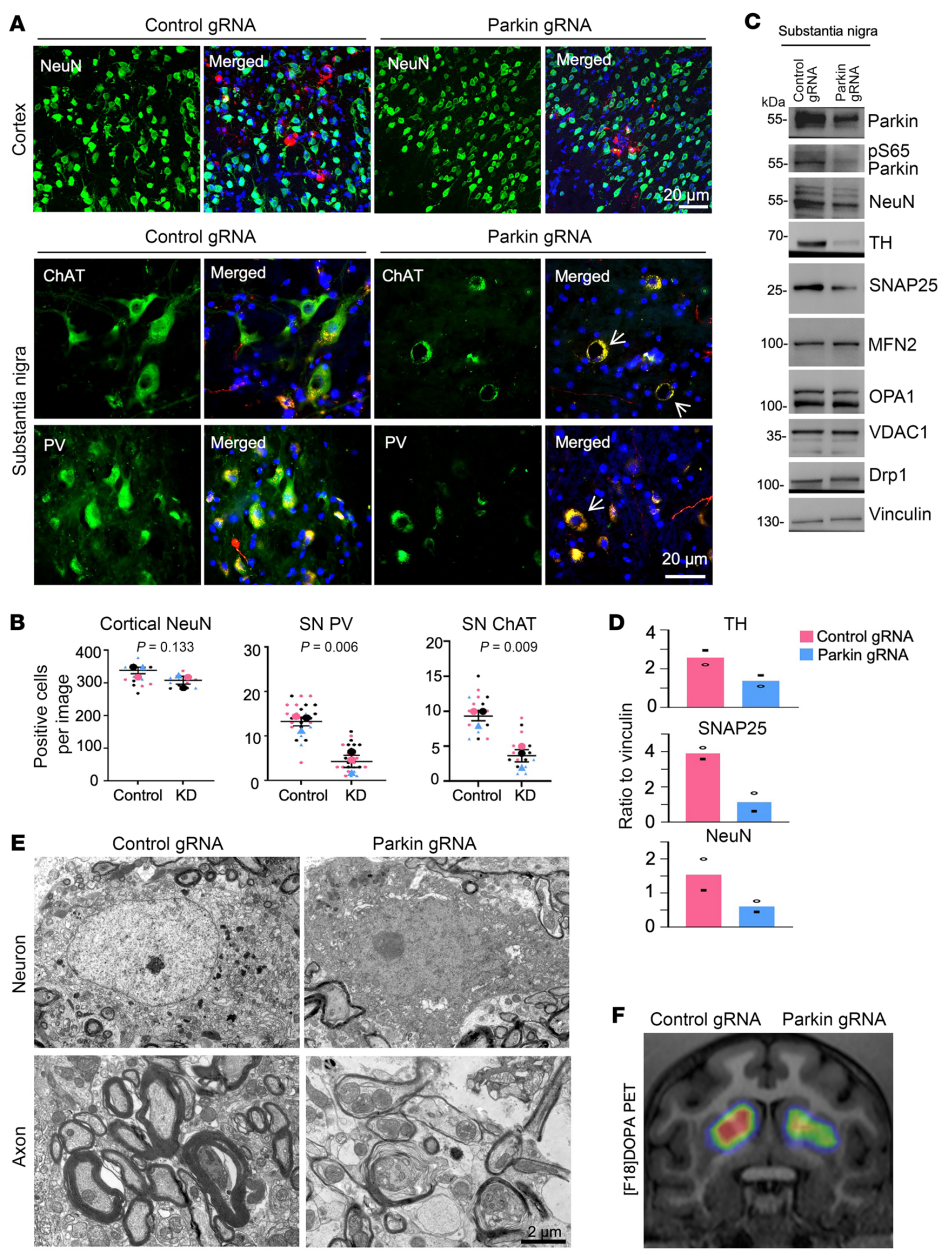
Selective neurodegeneration in the SN of monkeys targeted by AAV9-parkin gRNA/Cas9. (**A**) Immunostaining of the 6-year-old monkey prefrontal cortex and SN injected with control AAV9 gRNA or AAV9-parkin gRNA/Cas9. Antibodies to NeuN, PV, and ChAT were used to identify neurons while anti-RFP was used to show viral infection. Representative images of multiple technical replicates from 3 animals. (**B**) Superplot analysis of the number of cortical NeuN-, SN PV–, and ChAT-positive neurons in control gRNA/Cas9– and parkin gRNA/Cas9–injected monkeys, with each group comprising 3 monkeys. For each image captured at ×20 magnification, the number of NeuN-, PV-, or ChAT-positive cells was recorded and color coded to represent the specific monkey brain it originated from. The average count of these cells in each animal was used for paired 2-tailed *t* test to obtain *P* values. Data are represented as means ± SEM (*n* = 3 each group). (**C**) Western blotting analysis of neuronal and mitochondria proteins in the control and parkin KD SN of 6-year-old monkeys. (**D**) Ratios of parkin, S65-parkin, NeuN, TH, and SNAP25 to vinculin on Western blots from 2 independent experiments. (**E**) EM indicates that targeting parkin by AAV9 parkin gRNA/Cas9 resulted in degenerated neurons (upper panels) and axons (lower panels) in the SN as compared with the AAV control gRNA SN in a 6-year-old monkey. Representative images of multiple technical replicates from 1 control gRNA– and 1 parkin gRNA/Cas9-injected monkey. (**F**) Representative 18F-DOPA PET/CT imaging shows the reduction of dopamine in the striatum 2 months after injection of AAV parkin gRNA/Cas9 into the SN at one side in an adult monkey at age 8 years. The other side SN of the same monkey was injected with control gRNA/Cas9.

The AAV9-gRNA vector also expressed red fluorescent protein (RFP), allowing for the identification of the injected brain regions through coexpression of RFP with gRNA/Cas9 (Figure 2B and Supplemental Figure 5A). T7E1 assays confirmed mutations in the parkin gene in the AAV9-parkin gRNA/Cas9–injected monkey brains (Supplemental Figure 5B). In the prior experiments to determine efficiency of viral infection by stereotaxic injection, we verified coexpression of AAV9-gRNA/RFP and AAV9-Cas9 in the same tyrosine hydroxylase–positive (TH-positive) neurons, which represent dopaminergic neurons, 4 weeks after both viral vectors were coinjected into the monkey SN (Supplemental Figure 5C). In the monkey SN, dopaminergic neurons are large cells with large-sized nuclei that are less labeled by Dapi than the nuclei of other types of cells. In the AAV9-injected SN, the majority of neurons that were infected by AAV and expressed RFP were those large-sized neurons that were also positive for TH staining (Supplemental Figure 5D). Coimmunostaining of parkin demonstrated that parkin was reduced in AAV-infected cells (Figure 2C). We then used double immunofluorescent staining to identify the AAV-infected areas (red fluorescence), and neuronal cells (green) labeled by antibodies to neuronal proteins (NeuN) in the SN of adult monkeys (6–28 years old). Compared with the AAV control gRNA injection (Figure 2D), there was a reduction of TH-positive neurons in the AAV parkin gRNA/Cas9–injected SN (Figure 2E). Quantification of the relative numbers of neuronal cells confirmed that targeting *PARK2* resulted in loss of TH neurons in the SN, and this loss appeared to be more severe in the old monkeys (Figure 2F). However, targeting *PARK2* in the cortex of 6- and 8-year-old monkeys via injection of AAV parkin gRNA/Cas9 did not induce significant loss of neuronal cells as compared with the control gRNA/Cas9 injection (Figure 3, A and B). Examination of different types of neurons in the SN with antibodies to choline acetyltransferase (ChAT) and parvalbumin (PV) demonstrated that these neurons were also degenerated compared with those in the control gRNA-infected region (Figure 3, A and B). Additionally, increased fluoro–Jade C staining (Supplemental Figure 6, A and B) and light immunocytochemical (DAB) staining of the decreased TH-positive neurons (Supplemental Figure 6C) in the 12-year-old monkey injected with AAV parkin gRNA/Cas9 revealed consistent neurodegeneration.

We then used different approaches to confirm the *PARK2* targeting-induced SN neurodegeneration revealed by immunofluorescent staining. Western blotting serves as a crucial validation of immunohistochemical assays, as it enables the assessment of multiple proteins in the same tissues. Western blotting analysis of the 9-year-old monkey targeted by control gRNA or parkin gRNA in the SN showed that parkin and its phosphorylated form pS65-parkin were reduced in the parkin-targeted SN, accompanied by the reduction of neuronal proteins (TH and SNAP25) without alterations in mitochondrial proteins (MFN2, OPA1, VDAC1, and Drp1) (Figure 3, C and D). Electron microscopy offered ultrastructural evidence for degeneration of neuron and axons in the *PARK2*-targeted SN when compared with the control RNA–injected SN (Figure 3E). To determine whether dopaminergic neurons also exhibit degeneration of their axons projected to the striatum, we used a PET imaging technique that utilizes the radiotracer 18F-DOPA to monitor dopamine release from dopamine neuronal processes in the striatum. By comparing to the contralateral SN injected with AAV control gRNA, the SN injected with AAV parkin gRNA/Cas9 led to reduction of 18F-DOPA in the striatum, also indicating degeneration of dopamine neurons and their processes (Figure 3F). However, astrocytes did not appear to be affected by targeting *PARK2* in the AAV-injected monkey brain regions (Supplemental Figure 7). Thus, using multiple approaches to assess neuronal morphology and function, we provided robust evidence that parkin deficiency can cause degeneration of SN dopaminergic neurons in adult monkeys.

### Parkin phosphorylation relies on PINK1 expression in the primates.

**Figure 4:**
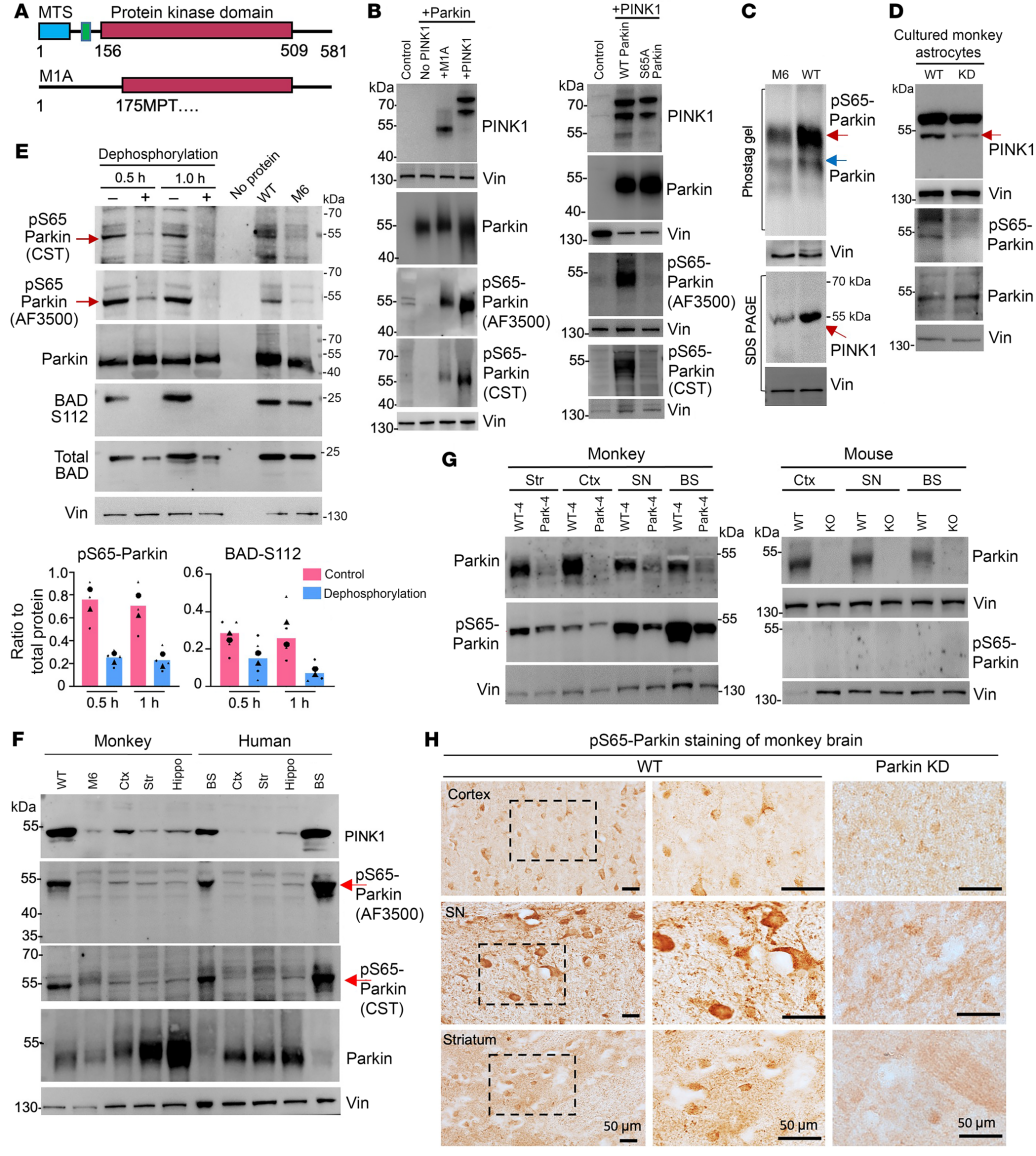
The phosphorylation of parkin at S65 relies on PINK1 expression in the primates. (**A**) The structure schematic of human full-length PINK1 that contains a mitochondrial targeting sequence (MTS), a transmembrane domain (TM), and a kinase domain (KD), and a mutant *PINK1* (M1A) that was generated by replacing methionine with alanine at amino acid position 1. (**B**) The plasmids of full-length PINK1 and PINK1 (M1A) were cotransfected with WT human parkin plasmid in 293 cells for 48 hours, and the cells were collected for Western blotting analysis. Two different anti-pS65-parkin (AF3500 and CST-36866S) antibodies were used to detect parkin phosphorylation. Western blotting results show that parkin phosphorylation at S65 occurred only in the presence of PINK1. (**C**) Phos-tag gel analysis also shows that PINK1 deficiency in the M6 monkey brain cortex reduces phosphorylation of pS65-parkin. (**D**) Knocking down PINK1 via siRNA in cultured monkey astrocytes reduced phosphorylation of parkin (pS65-parkin). (**E**) Brain stem (BS) lysates from a WT monkey at the age of 8 years were treated with alkaline phosphatase (1 U/μL) for 0.5 and 1 hour at 37°C to dephosphorylate endogenous proteins. Western blotting analysis revealed that dephosphorylation eliminated the immunoreactivity of pS65-parkin to the 2 antibodies (AF3500 and CST) specific to phosphorylated parkin. Phosphorylated S112-BAD served as a control. The *PINK1* mutant (M6) monkey BS tissues were included to show that endogenous pS65-parkin (red arrows) is reduced by knocking down PINK1. The ratios of phosphorylated proteins to total proteins from 4 independent experiments are presented beneath the blot (*n* = 2 animals). (**F**) Western blotting analysis of parkin, PINK1, and pS65-parkin expression in different brain regions in monkey (3 years old) and human (68 years old) who had passed away due to cancer. The level of pS65-parkin (arrows) recognized by 2 antibodies is dependent on the level of PINK1, but not the total parkin. Hippo, hippocampus. (**G**) Reduced parkin and pS65-parkin in different brain regions of a 3-year-old parkin mutant monkey (Park-4) when compared with a 3-year-old WT monkey. However, there was no specific alteration of pS65-parkin signals in parkin KO mouse brain tissues. Three independent experiments were conducted. Vin, vinculin. (**H**) Representative images of immunostaining of pS65-parkin in different brain regions (cortex, SN, striatum) of an 8-year-old monkey also showing that pS65-parkin is more abundant in the SN compared with other regions of monkey brains. The adult brain regions (cortex, striatum, and SN) injected with AAV9 parkin gRNA/Cas9 (parkin KD) show reduced levels of pS65-parkin. Representative Western blotting results and immunostaining images are from at least 3 independent experiments of 2 biological replicates.

Since parkin is activated by PINK1’s phosphorylation at its S65 position (4, 31–34), it is important to investigate the role of phosphorylated parkin in neurodegeneration. However, despite strong evidence for in vitro phosphorylation of parkin by PINK1, there is lack of in vivo evidence for parkin phosphorylation in the animal brains. Although we found that the PINK1 kinase form that lacks the N-terminal mitochondria targeting domain is uniquely expressed in the primate brains (30, 35), it remains to be investigated whether PINK1-mediated parkin phosphorylation is involved in neurodegeneration. To do so, it is crucial to confirm the ability of an antibody to specifically recognize phosphorylated parkin in a PINK1-dependent manner. Using transfected HEK293 cells expressing full-length human PINK1 or a truncated PINK1 (M1A) that contains the majority of the kinase domain (Figure 4A), we first verified that 2 anti-parkin antibodies we used could specifically react with pS65-parkin when PINK1 was expressed (Figure 4B). Moreover, the immunoreactivity of anti-pS65 parkin antibody could be eliminated by mutation of S65 to A65 in parkin (Figure 4B).

We also used different techniques and PINK1 KD (M6) monkey tissues (29) to rigorously verify the specificity of the antibody to phosphorylated parkin. First, phos-tag gel experiments have demonstrated that PINK1 induced by carbonyl cyanide m-chlorophenyl hydrazone (CCCP) or overexpressed PINK1 can yield abundant phosphorylated parkin in cultured cells (39, 40). Using phos-tag gel analysis, we found that PINK1 deficiency in the M6 monkey cortex reduced pS65-parkin phosphorylation as compared with WT monkeys (Figure 4C). Second, to determine whether the phosphorylation signals of parkin are dependent on PINK1, we used siRNA to inhibit PINK1 in cultured monkey astrocytes and verified that reducing PINK1 decreased pS65-parkin phosphorylation (Figure 4D). Third, to verify that antibodies for pS65-parkin reacted with endogenous phosphorylated parkin, we treated the monkey cortical tissue lysates with alkaline phosphatase (1 U/μL) for 0.5 and 1 hour at 37°C to dephosphorylate endogenous proteins. This treatment eliminated pS112-BAD phosphorylation and also diminished the immunoreactivity of pS65-parkin, which was also reduced in the M6 brain cortex, to the 2 antibodies specific to phosphorylated parkin (Figure 4E, arrows).

If PINK1 phosphorylates parkin in the monkey brains, we should observe that the levels of phosphorylated parkin are correlated with the expression levels of PINK1. Probing different regional tissues of monkey and human brains with 2 antibodies against pS65-parkin revealed that phosphorylation of parkin at S65 was indeed dependent on the expression level of PINK1, but not total parkin (Figure 4F). Furthermore, in the brain tissues of the parkin KD monkey (Park-4), pS65-parkin immunosignals were obviously reduced compared with those in the WT monkey, whereas no specific pS65-parkin signals were detected in the WT and *parkin* KO mouse brain tissues (Figure 4G). The lack of mouse pS65-parkin is consistent with the fact that PINK1 is undetectable in the mouse brain (30, 35). Immunostaining of the cortex, SN, and striatum of the WT monkey brain also showed higher levels of pS65-parkin in the SN neurons, which was diminished by knocking down parkin via AAV CRISPR/Cas9 injection (Figure 4H). All these findings from independent experiments using different methods confirmed the specificity of pS65-parkin antibodies in recognizing phosphorylated parkin at S65 and provide what we believe is the first in vivo evidence for PINK1-dependent parkin phosphorylation in the animal brain under physiological conditions.

### Aging affects parkin phosphorylation and increases α-syn accumulation.

**Figure 5:**
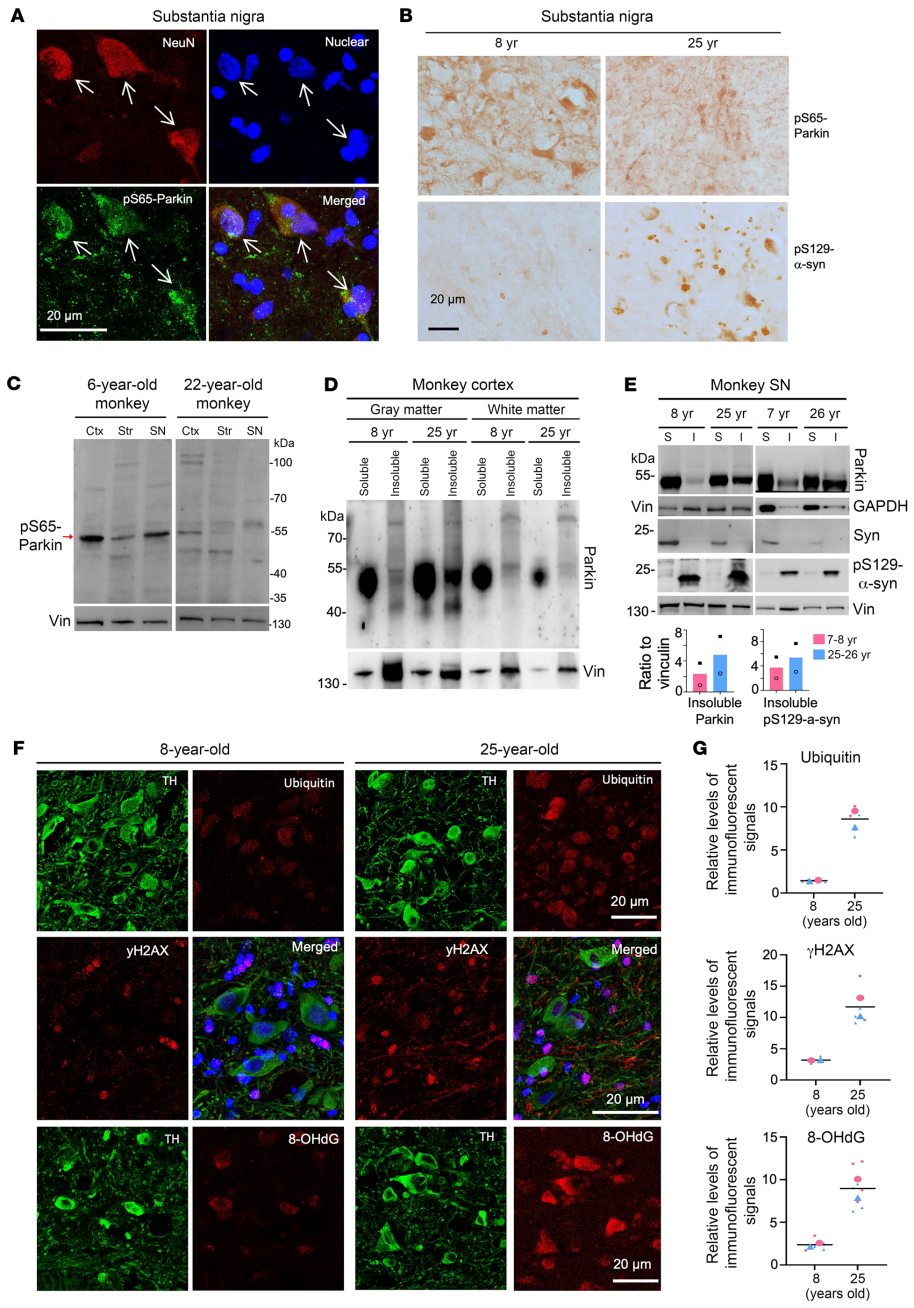
Age-dependent reduction of pS65-parkin phosphorylation and increase of pS129-α-syn. (**A**) pS65-parkin is more abundant in neuronal cells in the SN. The large-sized neurons (arrows) in the SN could be identified by double immunostaining with mouse anti-NeuN and rabbit anti-pS65-parkin. (**B**) Representative images of immunostaining showing decreased pS65-parkin and increased pS129-α-syn in the SN of the 25-year-old monkey when compared with the 8-year-old monkey brain. (**C**) Western blotting revealing age-dependent decline of pS65-parkin in the monkey brains. (**D**) Western blotting analysis was performed to examine parkin distribution in the soluble and insoluble fractions of the gray matter and white matter of the monkey brain cortex at 8 and 25 years of age. (**E**) The soluble (S) and insoluble (I) protein fractions were extracted from the SN tissues of young (7 and 8 years) and old (25 and 26 years) monkeys. Western blot analysis revealed increases in parkin and pS129-α-syn in the insoluble fraction of the aged monkey brains. The ratios of parkin and pS129-α-syn to vinculin are presented beneath the blots. In **C**–**E**, Western blot images represent the results in 3 independent experiments. (**F**) Representative images of immunostaining demonstrated an increase in γH2AX, 8-OhdG, and ubiquitination in the SN of the 25-year-old monkey compared with the 8-year-old monkey. (**G**) Quantification of the relative levels of immunostaining signals in **E** using 4–8 brain sections of each group (*n* = 2 animals per group). Representative Western blotting results and immunostaining images are from at least 3 independent experiments of 2 biological replicates.

After being able to detect parkin phosphorylation, we proceeded to investigate the relationship between parkin phosphorylation and the accumulation of pS129-α-syn, which is a pathological hallmark of PD (2). We found that phosphorylated parkin (pS65-parkin) is enriched in the TH neurons in the monkey SN (Figure 5A). Comparing with the SN in a young monkey (8 years), pS65-parkin staining appeared to be decreased and pS129-α-syn staining was increased in the SN of a 25-year-old monkey (Figure 5B). Similarly, pS65-parkin is also enriched in NeuN-positive neuronal cells in the striatum (Supplemental Figure 8A) and was reduced in the striatum of a 25-year-old monkey, accompanied by increased levels of pS129-α-syn (Supplemental Figure 8B). To confirm these findings, we analyzed monkeys at 6 and 22 years of age using Western blotting, an assay that can simultaneously detect pS129-α-syn and other proteins in the same samples. The results showed that pS65-parkin declined in different brain regions (cortex, striatum, SN) in the older monkey (Figure 5C).

Previous studies have reported an age-dependent increase in the insolubility of parkin in human brain tissues (41). In our examination of the monkey cortex, we found that this phenomenon was particularly prominent in the gray matter compared with the white matter (Figure 5D). By comparing the soluble and insoluble forms of parkin in the SN of both young (7 and 8 years) and old (25 and 26 years) monkeys, we also found that aging increased the amount of insoluble parkin and pS129-α-syn (Figure 5E). Since insoluble parkin is unlikely to function normally like soluble parkin, aging could impair the function of parkin. Considering that aging enhances oxidative stress, which can impact protein solubility and conformation (41), we then compared oxidative stress products in the SN of young (8 years) and old (25 years) monkey brains. The old monkey SN exhibited higher levels of yH2AX and 8-OHdG, 2 oxidative stress products (42, 43), compared with the young monkey (Figure 5, F and G). Together, aging-related products including oxidative stress appear to disrupt parkin phosphorylation in association with the accumulation and toxicity of pathological α-syn.

### Parkin deficiency resulted in the accumulation of pathological α-syn.

pS129-α-syn is known to be toxic to neurons and exhibits misfolding and aggregation in the brain (44–46). Parkin has been found to be involved in removing a number of misfolded proteins via complex mechanisms (47-49), and there are inconsistent results regarding whether pS129-α-syn is cleared by the proteasome or autophagy (50–52). Thus, it is important to verify the causative role of parkin deficiency in the accumulation of pS129-α-syn in the primate brain.

**Figure 6:**
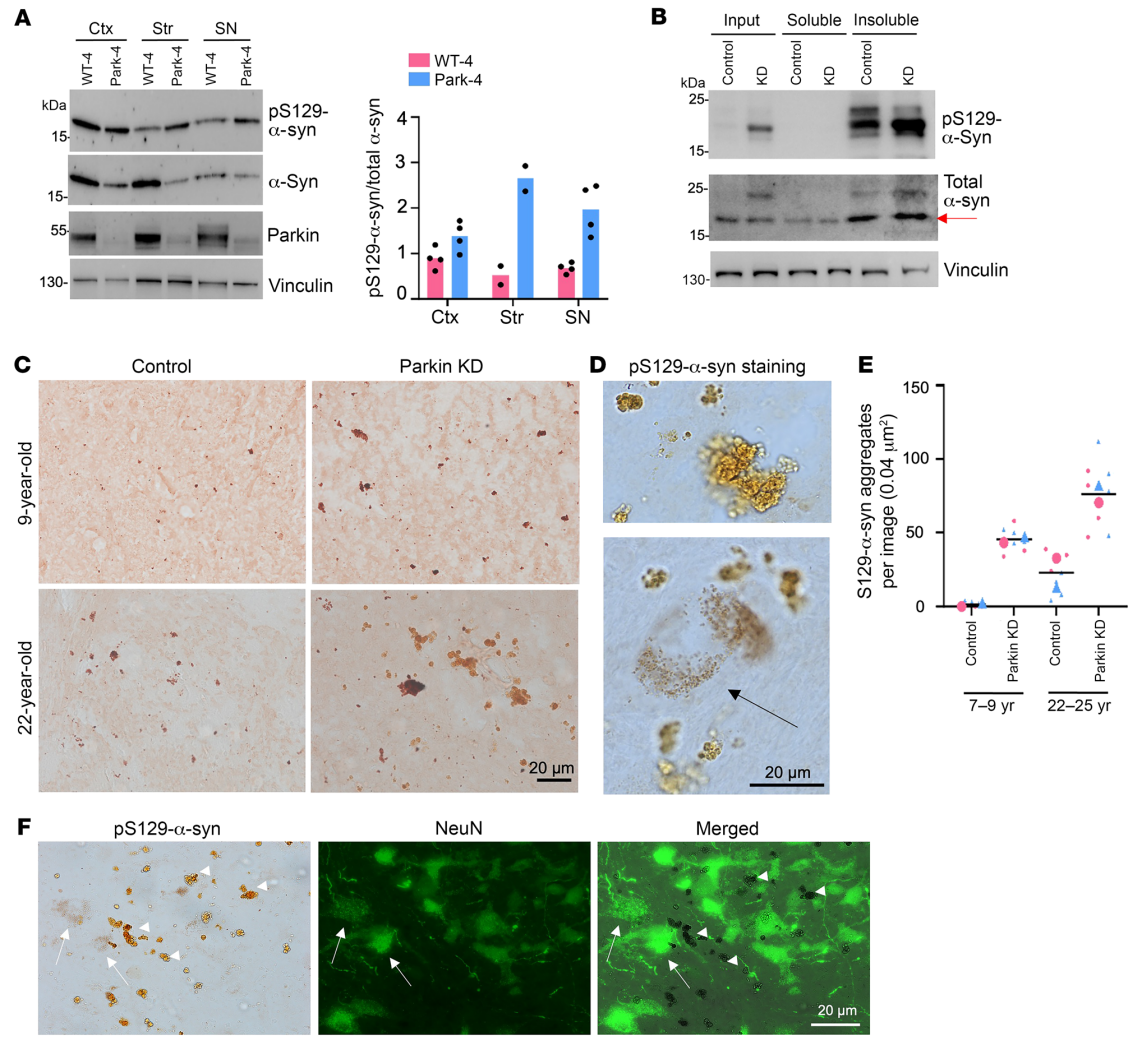
Parkin deficiency increased pS129-α-syn accumulation. (**A**) Increased pS129-α-syn in parkin-deficient monkey (Park-4) as compared with a 3-year-old WT monkey. The ratios of pS129- α-syn to total α-syn from 2-4 independent Western blotting assays are presented (right panel). (**B**) Western blotting showing that targeting parkin via injection of AAV9-parkin gRNA/Cas9 into the striatum of an aged monkey (22 years old) increased the amount of insoluble pS129-α-syn. At least 3 independent experiments using monkey brain tissues from 1 WT and 1 parkin KD were performed, and representative images are presented. (**C**) Representative images of immunostaining of the SN of the young (9 years old) and old (22 years old) monkeys injected with AAV9-parkin gRNA/Cas9 revealing the increased staining of pS129-α-syn as compared with AAV9 control gRNA/Cas9 injection. (**D**) High-magnification micrographs showing cytoplasmic pS129-α-syn accumulation (arrow) and aggregates in a 22-year-old monkey. (**E**) Quantification of the numbers of pS129-α-syn aggregates in the SN of young (7 and 9 years old) and old (22 and 25 years old) monkeys. The injected SN regions were used to obtain the average numbers of pS129-α-syn aggregates (×40 or per 0.04 μm^2^) in multiple images. The number of aggregates was recorded and color coded to represent the specific monkey brain it originated from. (**F**) DAB staining of pS129-α-syn and immunofluorescent staining of NeuN allowed for detection of pS129-α-syn aggregates, which are present both intracellularly, within the cell body (arrows), and extracellularly, outside the cell body or in the neuronal processes that may not be identifiable by staining. Representative immunostaining images are from multiple technical replicates of at least 2 monkey brains.

Since we obtained a 3-year-old *PARK2* mutant (Park-4) monkey in which the *PARK2* gene was targeted by CRISPR/Cas9 in the fertilized egg, the brain tissues of this unique monkey model provided us with valuable materials to analyze the effect of knocking down parkin on pS129-α-syn accumulation. Western blotting showed increased levels of pS129-α-syn in the cortex, striatum, and SN of the parkin KD monkey compared with the age-matched WT monkey (Figure 6A). These results highlight how parkin KD can induce the accumulation of S129-α-syn prior to neurodegeneration in the young animals. Given that the accumulation of misfolded or toxic proteins typically precedes neurodegeneration in various neurodegenerative conditions including PD (53), our findings further suggest the potential causal role of parkin phosphorylation deficiency in neurodegeneration associated with PD. Even knocking down parkin in the aged (22 year old) monkey cortex through the injection of AAV9-parkin gRNA/Cas9 could also increase insoluble pS129-α-syn compared with the WT monkey cortex (Figure 6B). Since aging is a great risk factor for the development of PD pathology, we next examined whether aging can promote parkin deficiency–mediated accumulation of pS129-α-syn in the SN. To this end, we performed stereotaxic injection of AAV9-parkin gRNA/Cas9 in the SN of young (7 and 9 years old) and old (22 and 25 years old) monkeys and analyzed the injected brain regions 2 months after injection. Although the limited SN with AAV infection did not allow for a subcellular fractionation assay, immunostaining clearly demonstrated a higher abundance of pS129-α-syn aggregates in the old monkey SN when parkin was knocked down as compared with the control gRNA injection region (Figure 6C). High-magnification micrographs revealed aggregate-like structures and cytoplasmic accumulation of pS129-α-syn (Figure 6D, arrow). pS129-α-syn aggregates were found in various forms in the brains of PD patients (24, 27, 54–57), potentially influenced by their posttranslational modifications during disease progression and the sensitivities of antibodies in recognizing different misfolded α-syn forms. Interestingly, the aggregated pS129-α-syn in monkey brains resembled those detected in some PD patients’ brains (24, 27, 54, 56, 57)). Notably, the amounts of pS129-α-syn are higher in the aged monkey SN (22 and 25 years) compared with the young monkeys (7 and 9 years) (Figure 6E). Immunodouble staining confirmed that these aggregated pS129-a-syn were present both intracellularly, within the cell body, and extracellularly, outside the cell body or in the neuronal processes that may not be identifiable by staining (Figure 6F). These results imply that both aging and parkin deficiency contribute to the accumulation of toxic α-syn products.

### Parkin phosphorylation deficiency associates with the accumulation of pS129-α-syn.

**Figure 7:**
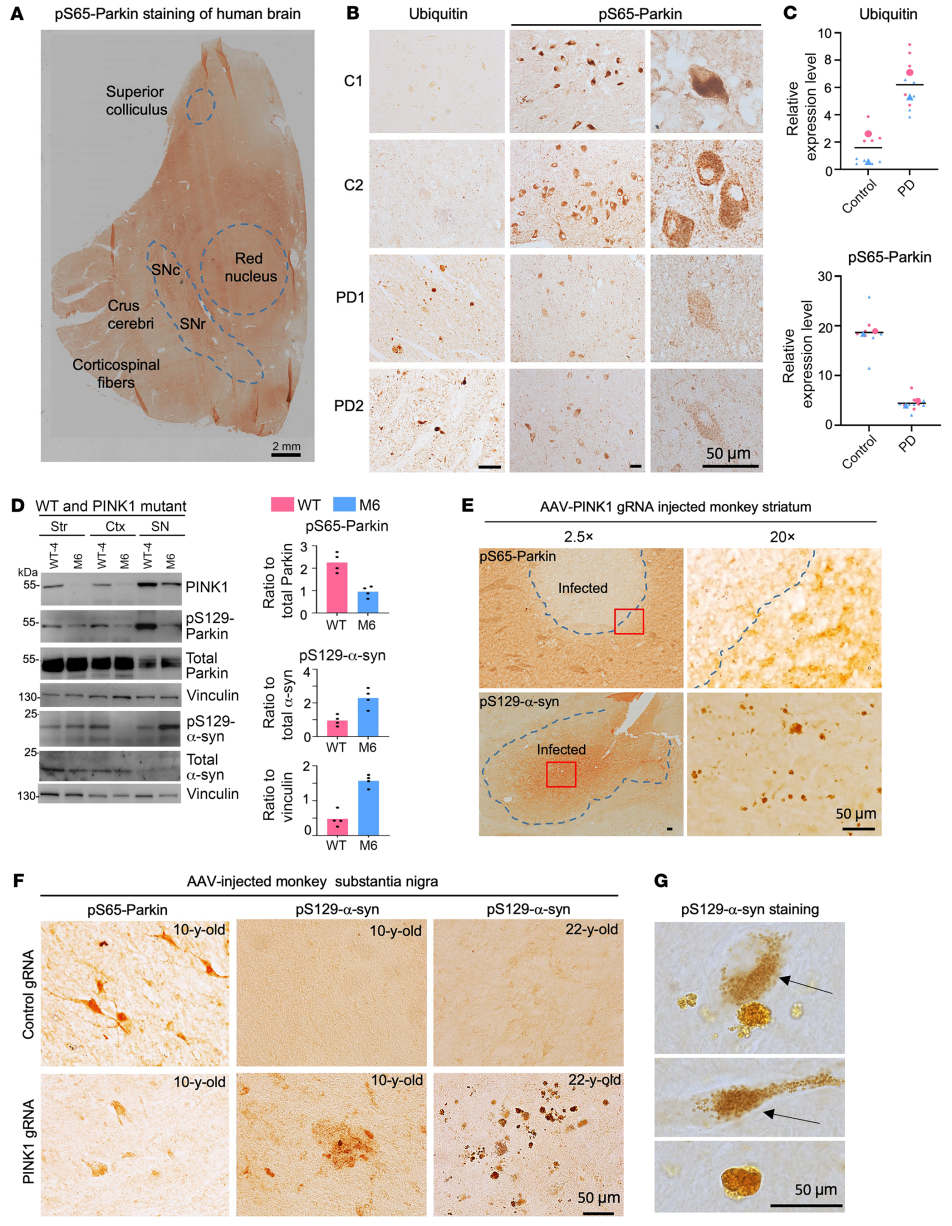
Reduced parkin phosphorylation and increased pS129-α-syn in the brains of PD monkey models and patients. (**A**) Low magnification of a human brain section containing the SN for examination. (**B**) In postmortem brains of 2 normal individuals (C1 and C2) and sporadic PD patients (PD-1 and PD-2), pS65-parkin expression is reduced in the SN neurons in PD patient brains. Scale bar: 2 mm. (**C**) Quantification of the relative expression levels of ubiquitin and pS65-parkin in **B**. Two human brain samples for each group were used for quantification: 2 PD cases (PD-1: 77 years, female; and PD-2: 55 years, female) and 2 control cases (C-1: 48 years, female; and C-2: 54 years, male). Representative immunostaining images are from multiple technical replicates of 2 human brains. (**D**) Reduced pS65-parkin (S65) and increased pS129-α-syn occurred in the SN of *PINK1* mutant monkey (M6, 3 years old) (left panel). Ratios of pS65-parkin to total parkin or pS129-α-syn to total α-syn or vinculin (*n* = 4 independent experiments each group) are also presented (right panel). (**E**) Representative images of reduced pS65-parkin and increased pS129-a-syn occurred in the SN of a 10-year-old monkey that was injected with AAV9-PINK1 gRNA/Cas9. (**F**) Representative images of PINK1 reduction by AAV9-PINK1 gRNA/Cas9 injection led to more pS129-α-syn accumulation in the SN of an older (22 years old) monkey than a younger (10 years old) monkey. (**G**) High-magnification micrographs showing cytoplasmic pS129-α-syn accumulation and aggregates. Arrows indicate cytoplasmic labeling of pS129-α-syn. In **E**–**G**, representative immunostaining images are from multiple technical replicates of 3 WT monkey brains.

Since parkin phosphorylation is necessary for its activity and since gaining and genetic mutations of *PARK2* or *PINK1* may affect this activity, we next investigated whether parkin phosphorylation deficiency is associated with pS129-α-syn accumulation in the SN of PD patient brains. Immunocytochemical staining revealed that pS65-parkin was enriched in the SN neurons of a normal human brain (Figure 7A and Supplemental Figure 9A). To explore whether there is reduced pS65-parkin in the SN of sporadic PD patient brains, we examined 2 normal human brains (C1 and C2) and 2 PD brains (PD-1 and PD-2) that did not carry *PINK1* or *PARK2* mutations, but displayed ubiquitinated aggregates (Figure 7B) and Lewy bodies (Supplemental Figure 9B) in the SN. pS65-parkin was decreased in the SN of PD patient brains, accompanied by increased ubiquitin-positive aggregate staining (Figure 7B). We quantified pS65-parkin staining intensity in the remaining SN neurons in the normal human and PD SN and found a statistically lower (*P* < 0.001) level of pS65-parkin in PD brains (Figure 7C). Thus, deficient pS65-parkin phosphorylation also occurred in the SN of PD patient brains that exhibit the accumulation of pathological α-syn.

The above findings suggest that parkin phosphorylation deficiency can lead to the accumulation of pS129-α-syn. Since parkin phosphorylation at S65 is mediated by PINK1, we next explored whether PINK1 dysfunction or deficiency could also cause the phenomenon similar to those mediated by loss of parkin. Using the brain tissues from a 3-year-old WT monkey and *PINK1* mutant monkey (M6) (30, 58), we confirmed that PINK1 deficiency diminished pS65-parkin, and this effect was most prominent in the SN despite its lower level of total parkin (Figure 7D). Next, we injected AAV-PINK1 gRNA/Cas9 into the striatum and SN of 10- and 22-year-old WT monkeys to KD PINK1. The injected monkey striatum showed a clear reduction in pS65-parkin staining and increase in pS129-α-syn labeling and accumulation (Figure 7E). Immunocytochemistry verified that PINK1 deficiency reduced pS65-parkin and caused a greater accumulation of pS129-α-syn aggregates in the old monkey’s SN (Figure 7, F and G). Moreover, cytoplasmic pS129-α-syn accumulation and aggregate formation (Figure 7F) were almost identical to those caused by parkin deficiency (Figure 6D).

### Overexpression of phosphorylated parkin reduced the accumulation of pS129-α-syn.

**Figure 8:**
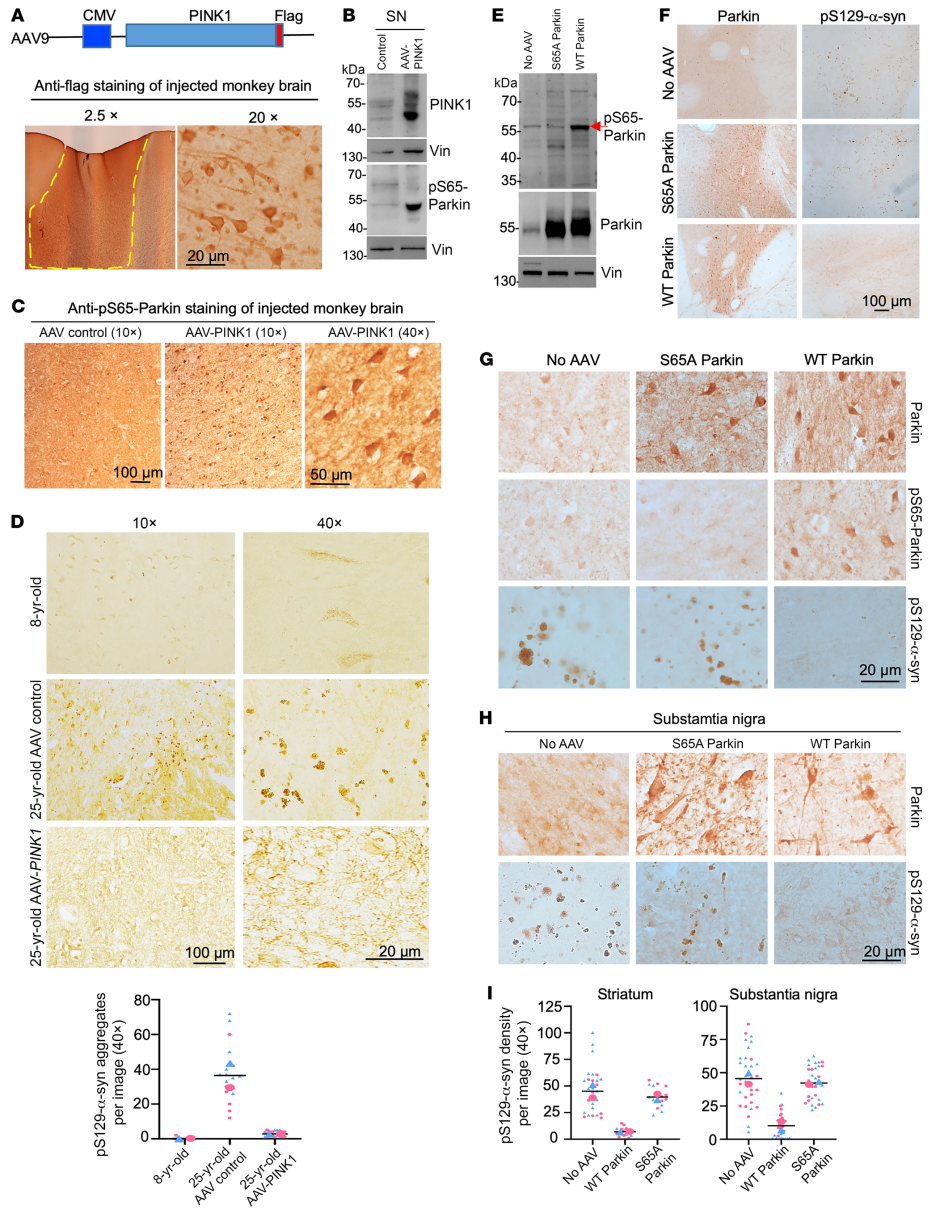
Overexpression of parkin and PINK1 increased the expression of pS65-parkin and reduced pS129-α-syn accumulation in the monkey brain. (**A**) AAV9-PINK1-Flag was injected into the cortex and SN of a 25-year-old WT monkey. The animal was analyzed 9 weeks later. Immunostaining with anti-flag antibody showing that the transgenic PINK1 could be unambiguously detected in the injected brain cortex. (**B** and **C**) Representative results of Western blotting (**B**) and immunostaining (**C**) showing that PINK1 overexpression in the 25-year-old monkey SN increased pS65-parkin phosphorylation. (**D**) The accumulation of pS129-α-syn in the old (25 years old) monkey SN was much more abundant than in the young (8 years old) monkey SN and could be reduced by overexpression of PINK1 via AAV9-PINK1-Flag injection. The density of aggregates per image is shown beneath the images (*n* = 2 animals per group). (**E**–**H**) AAV9-WT parkin and AAV9-S65A parkin were injected into the striatum and SN of old WT monkeys aged 22–25 years. The animals were analyzed 9 weeks later. Western blotting of the SN shows that only WT parkin, not mutant parkin (S65A), is phosphorylated (**E**, arrow). Representative immunostaining images (low magnification in **F** and high magnification in **G**) show that the transgenic WT parkin and S65A parkin could be unambiguously detected in the injected striatum tissues, and WT parkin, but not S65A parkin, reduced pS129-α-syn accumulation in the aged monkey brain. WT parkin, but not S65A parkin, was also able to reduce pS129-α-syn accumulation in the SN of the old monkeys (**H**). (**I**) Quantification of pS129-α-syn immunostaining density in the monkey striatum and SN injected with AAV WT parkin or AAV S65A parkin. The number of pS129-α-syn aggregates in each image (×40) was counted and color coded for 2 animals (22 and 25 years old). Representative Western blotting results and immunostaining images are from multiple technical replicates of 1 monkey (**A**–**C**) or 2 monkeys (**D**–**H**) for each group.

To further establish the role of parkin phosphorylation in the clearance of pS129-α-syn, we conducted rescue experiments by overexpressing AAV9-flag–tagged PINK1 via stereotaxic injection into one side of the monkey SN and an AAV control vector into the contralateral side of the SN in the same animal (Figure 8A). This approach allowed for clear detection of transgenic PINK1 using anti-Flag antibodies (Figure 8A). Consistently, the results showed a significant increase in pS65-parkin phosphorylation in the SN following AAV9-PINK1 injection (Figure 8, B and C). Although pS129-α-syn levels were higher in the SN of the older (25 year old) monkey compared with the younger (8 year old) monkey, overexpression of PINK1 effectively reduced the accumulation of pS129-α-syn in the SN of the older monkey (Figure 8D), further supporting that activated parkin by PINK1 can promote the clearance of endogenous pS129-α-syn in monkey brain.

**Figure 9:**
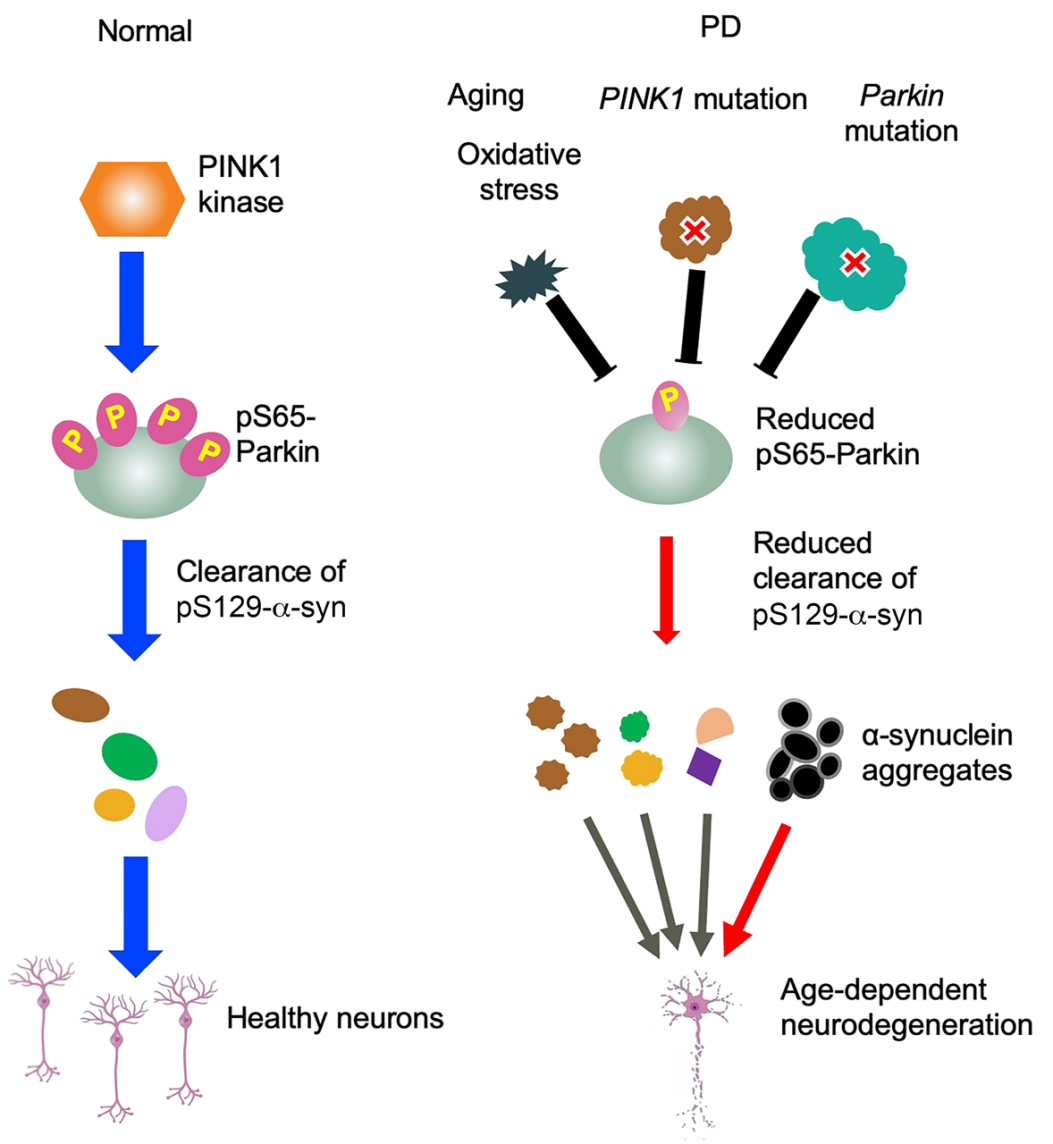
A proposed model for the critical role of pS65-parkin in PD pathogenesis. Aging-related oxidative stress and *PINK1/PARK2* mutations can reduce parkin activity, leading to the increased accumulation of toxic proteins including pS129-α-syn and selective SN neurodegeneration.

To further validate the importance of parkin phosphorylation in reducing pS129-α-syn accumulation, we performed stereotaxic injection of AAV-WT parkin and AAV-mutant (S65A) parkin, which cannot be phosphorylated by PINK1, into the striatum and SN of older monkeys aged 22 to 25 years. The overexpression of these 2 forms of parkin was confirmed through parkin Western blot (Figure 8E) and immunostaining (Figure 8F). Importantly, only WT parkin, not S65A parkin, was able to decrease pS129-α-syn accumulation in the striatum (Figure 8G) and SN (Figure 8H). Quantification of the relative density of pS129-α-syn in the striatum and SN of 2 older monkey brains verified that WT parkin, but not mutant parkin that is unable to be phosphorylated at S65, was able to reduce pS129-α-syn accumulation in the monkey striatum and SN (Figure 8I). These results strongly support the crucial role of parkin phosphorylation in the clearance of pS129-α-syn in primate brains. They are also consistent with previous reports of 2 PD patients with parkin (Ser65Asn) mutation (13), further suggesting the significance of parkin phosphorylation in primate brains. Thus, we propose that parkin phosphorylation at S65 plays a critical role in preserving the viability of SN neurons by eliminating toxic proteins, including pS129-α-syn. However, mutations in *PARK2* or *PINK1* and age-related oxidative stress can reduce parkin phosphorylation, leading to the accumulation of toxic proteins such as pS129-α-syn and ultimately contributing to age-dependent neurodegeneration in PD (Figure 9).

## Discussion

Although it is well known that parkin activity depends on its phosphorylation by PINK1, there is a lack of strong evidence to support the relationship between parkin phosphorylation and PINK1 in vivo, as PINK1 is rarely detected in small animals and cell lines under normal physiological conditions. Our recent studies discovered that PINK1 is selectively expressed in primate brains, but not in mouse or pig brains (30, 35). Thus, it is necessary to use primate brains to investigate how mutations in *PARK2* and *PINK1* can cause age-dependent neurodegeneration in PD. Our investigation using primate brain tissues indicates that parkin phosphorylation plays a critical role in selective neurodegeneration in PD and underscores the importance of understanding the in vivo function of parkin phosphorylation and its contribution to the development of PD.

There are limitations to using nonhuman primates for investigations, particularly due to the fact that the numbers of animals used cannot be as high as those used in studies involving small animals such as rodents. To validate important molecular and pathological changes in nonhuman primates, it is necessary to use at least 2 animals for biological replication (59). In our studies, we utilized multiple monkeys to validate significant findings, such as the SN degeneration caused by parkin deficiency and the association of defective parkin phosphorylation with the accumulation of pS129-α-syn. Additionally, we employed multiple assays to analyze the consequences of knocking down endogenous parkin. Our studies have uncovered several discoveries that warrant discussion.

First, we demonstrated for the first time, to our knowledge, that targeting the *PARK2* gene can cause SN neurodegeneration in the primate models, which is distinct from the lack of neurodegeneration in *Park2* KO mouse and pig models (18–20). Unlike the absence of noticeable neuronal loss in young (<3 years) monkeys following *PARK2* gene KD, adult monkeys (>6 years) exhibited SN neurodegeneration after targeting of the *PARK2* gene. These distinctions lend support to the hypothesis that parkin-mediated neuronal loss is contingent on age. However, whether neuronal loss is much more severe in the older monkeys (>20 years) would require further studies of additional old monkeys. Notably, the complete loss of PINK1 due to large DNA deletion in the *PINK1* targeted monkey models can cause neuronal loss in developing monkeys (29, 30). It is highly likely that PINK1 is essential for neuronal survival whereas PINK1-mediated parkin phosphorylation is more relevant to age-dependent neurodegeneration. This is because PINK1 acts as a kinase, phosphorylating various proteins, not just parkin (29, 30, 58). On the other hand, neurodegeneration caused by parkin deficiency may require insults associated with aging. In support of this idea, aging can increase oxidative stress and parkin insolubility and reduce parkin phosphorylation. Thus, in addition to mutations in *PINK1* and *PARK2*, multiple factors and insults, including aging-related oxidative stress and environmental toxins, could affect parkin phosphorylation and are involved in age-dependent neurodegeneration.

Second, in vitro studies have shown that parkin remains inactive and only becomes active after phosphorylation by PINK1 (4, 31–34). Our findings from primate brains, where PINK1 is more abundant compared with small animal brains, provide what we believe is the first in vivo evidence for parkin phosphorylation in the animal brain. The viral vectors used in our study appeared to preferentially infect neuronal cells, especially those large-sized dopamine neurons in the SN. Thus, knocking down parkin via AAV infection is more likely to affect parkin-associated neuronal function. Consistently, the phosphorylation of parkin is particularly prominent in the SN, highlighting its critical role in maintaining the normal function of the SN, which is highly vulnerable in PD. Therefore, mutations in *PINK1* or *PARK2* may selectively or preferentially impact the SN, leading to phenotypes similar to those observed in other cases of idiopathic PD.

One of the most intriguing findings in our study is the association between parkin phosphorylation deficiency and the accumulation of pS129-α-syn, which has not been reported in the literature. It is known that the accumulation and aggregation of α-syn occur under various pathological conditions (60, 61). Although the in vitro interaction between parkin and α-syn has been reported, strong in vivo evidence supporting their endogenous association is lacking (62), possibly due to the very low level of PINK1 and inactivation status of parkin under physiological conditions. In primate brains, the association of parkin deficiency with the accumulation of pS129-α-syn is supported by several findings from our studies. First, Western blotting analyses demonstrated an increase in pS129-α-syn in monkey brain tissues deficient in parkin or PINK1. Second, the increased expression of pS129-α-syn was limited to the injected brain areas where *PINK1* or *PARK2* was targeted. Third, aging reduced parkin solubility and increased the accumulation of pS129-α-syn. The most compelling evidence is that overexpressing parkin effectively reduced the accumulation of pS129-α-syn in the brains of aged monkeys. Furthermore, this protective effect was dependent on parkin’s phosphorylation at S65, as mutating S65 to A65 in parkin resulted in the inability of parkin to reduce pS129-α-syn. These findings suggest that parkin phosphorylation deficiency plays a critical role in the pathogenesis of PD.

Although previous in vitro studies have shown that parkin deficiency leads to the accumulation of various substrates (63–66), it remains unknown how endogenous parkin removes misfolded proteins when its phosphorylation is impaired. The noticeable accumulation of pS129-α-syn has not consistently been observed in animal models lacking PINK1 or parkin. Considering that parkin phosphorylation is rarely detected in small animal models, the association between parkin phosphorylation deficiency and the accumulation of pS129-α-syn in our monkey models is especially intriguing. It is also worth noting the ongoing debate regarding the presence of Lewy bodies in postmortem brain tissues from patients with *PARK2* or *PINK1* mutations (62). Further investigation is warranted to determine whether these human brain tissues exhibit aggregated pS129-α-syn in a manner similar to what we observed in our monkey models.

We recently found that PINK1 and parkin exhibit distinct subcellular distribution, despite their colocalization on depolarized mitochondria (67). Therefore, although parkin and PINK1 have been found to cooperate in regulating mitophagy during mitochondrial stress, they perform independent functions in vivo under normal unstressed situations. PINK1 is likely to phosphorylate a large number of substrates, and its phosphorylation of parkin is particularly relevant to the pathology of PD. On the other hand, parkin phosphorylation is not only regulated by PINK1, but also influenced by aging-related factors. It is likely that the activation of parkin through phosphorylation enables its function in clearing misfolded proteins, whereas its nonphosphorylated form is unable to clear toxic pS129-α-syn. These findings highlight the critical role of phosphorylated parkin in the pathogenesis of PD. Considering that the accumulation of pS129-α-syn is found in the majority of PD cases and other pathological conditions (2, 61), our findings also suggest that improving parkin phosphorylation could be a promising therapeutic approach for treating PD and other diseases associated with Lewy bodies or the accumulation of toxic pS129-α-syn.

## Methods

### Sex as a biological variable.

For studies involving humans and/or animal models, sex was not considered as a biological variable.

### Supplemental material.

Additional details on methods are available in the Supplemental Methods.

### Animals.

Generation of *PARK2* mutant rhesus monkeys via embryonic injection of CRISPR/Cas9 was described in our early studies (29, 58) using the same gene-targeting approach on monkey embryos described in our previous studies (29, 68, 69). In vitro fertilization of the injected embryos and embryo transfer had yielded a number of aborted fetuses or stillborn monkeys that carried mutations in specific genes or were WT without any gene targeting. There were animals at different ages without brain damage, but their body injury required euthanasia. These animals and some old animals (>20 years) that also needed to be euthanized were used for isolation of their brain tissues after MRI examination. Some adult animals that were unable to generate offspring or could not be used for behavioral studies but had normal MRI images of their brain structures were used for brain stereotaxic injection. The brain tissues isolated from WT and gene-targeted rhesus monkeys were saved and used in the current studies. Information on monkeys used for the study is listed in the Supplemental Table 1. For viral injection of monkey brains, we used the method described in our previous studies (30, 68, 70). The monkeys were housed at Yuanxi Biotech Inc., Guangzhou, and Guangdong Landao Biotechnology Co. Ltd, Guangzhou.

Parkin-KO mice were generated by deletion of the mouse parkin gene (*Park2*) at exon 3–exon 6 using CRISPR/Cas9. *Park2*-KO mice were obtained from Gempharmatech Co. and maintained at the animal facility at Jinan University.

### Human brain tissues.

Postmortem brains and peripheral tissues were obtained from the Brain Bank of Xiangya School of Medicine through the willed body donation program and from the Hanson Institute Centre for Neurological Diseases & SA Pathology School of Medicine, University of Adelaide, Adelaide, Australia. Paraffin sections of the midbrain and hippocampal formation from 2 clinically diagnosed PD cases (PD-1: 77 years, female; and PD-2: 55 years, female) and 2 control cases who died of ovarian cancer and paraplegia, respectively (C-1: 48 years, female; and C-2: 54 years, male), were examined in the current study for the presence of Lewy pathologies, pS65-parkin, and pS129-α-syn. The postmortem delays of brain collection were 5 to 12 hours. Postmortem tissues from 2 individuals were used for Western blotting analysis; 1 died of breast cancer (68 years, female) with a postmortem interval of 4 hours, and 1 died of liver cancer (38 years, male) with a postmortem interval of 8 hours. All brains were also assessed for optimal histological integrity and Alzheimer-type neuropathology following a standard protocol proposed by the China Human Brain Banking Consortium (71, 72).

### Western blot analysis and electron microscopy.

For Western blot analysis, monkey brain tissues were lysed in ice-cold RIPA buffer (50 mM Tris, pH 8.0, 150 mM NaCl, 1 mM EDTA pH 8.0, 1 mM EGTA pH 8.0, 0.1% SDS, 0.5% DOC, 50 mM NaF, and 1% Triton X-100) containing Halt protease inhibitor cocktail (Thermo Scientific) and PMSF. The lysates were incubated on ice for 30 minutes, sonicated, and centrifuged at the 12,000*g* for 10 minutes. Equal amounts of proteins from the supernatants determined by BCA assay were resolved by SDS-PAGE and subjected to Western blot analysis with appropriate primary antibodies (see Supplemental Table 2). Independent Western blot experiments were performed at least 3 times, and representative results are presented in figures. Acquired images were subjected to densitometric quantitation using ImageJ software (NIH).

For electron microscopy (EM), the left and right sides of the SN in a 6-year-old monkey were injected with AAV control gRNA/Cas9 and AAV parkin gRNA/Cas9, respectively. Two months after the virus injection, this monkey was deeply anesthetized by intraperitoneal injection of 0.3–0.5 ml of atropine, followed by 10–12 mg of ketamine and 15–20 mg of pelltobarbitalum natricum per kg body weight. The freshly isolated brain tissues of SN were fixed with 2.5% glutaraldehyde/4% PFA in 1× PBS overnight at 4°C. Brains were sectioned into 50 μm using a vibratome (Leica, VT1000s), and the sections were processed for EM examination. In brief, all sections were osmicated in 1% OsO4 in 0.1 M PB and embedded in Eponate12 (Ted Pella).

### AAV viral preparation and stereotaxic injection.

CRISPR/Cas9-expressing viral vectors (PX551, PX552) were obtained from Addgene (plasmids 60957 and 60958). The AAV9-*PINK1*-gRNAs for monkeys were generated as in our previous studies (30). Using the same strategy, the AAV-*PARK2*-gRNAs were generated by inserting gRNAs into PX552 via SapI restriction sites. Sequences were as follows: *PARK2* target sequence 1: 5′-CTCCAGCCATGGTTTCCCAG-3′, *PARK2* target sequence 2: 5′-CAAGAAATGAATGCAACTGG-3′, and control gRNA: 5′-ACCGGAAGAGCGACCTCTTCT-3′. AAV9-CMV-Cas9 vector was generated by replacing Mecp2 promoter (228 bp) in PX551 with CMV promoter (658 bp) using XbaI and AgeI restriction sites. These viral vectors were packaged to generate purified AAV9 viruses. The genomic titer of viruses (vg) (approximate 10^13^ vg/ml) of the purified viruses, which were used for injection, was determined by PCR method.

Viral injection of monkey brains was performed using the method described in our previous studies (30, 68). The monkey brain regions were located by MRI before injection. Each monkey was anesthetized by intraperitoneal injection of 0.3–0.5 mL of atropine, followed by 10–12 mg of ketamine and 15–20 mg of pelltobarbitalum natricum per kg body weight. The monkeys were then stabilized on a stereotaxic instrument (David Kopf Instruments). Five to ten microliters of viruses were injected into one side of the monkey prefrontal cortex, striatum, or SN. The right sides of these brain regions were injected with mixed AAV9-Cas9 with AAV9-parkin gRNA (1:4 ratio). The left sides were injected with control AAV9-GFP or AAV9-control gRNA. Two to three months after injection, the monkeys were euthanized by deep anesthesia with intraperitoneal injection of 0.3–0.5 mL of atropine, followed by 10–12 mg of ketamine and 15–20 mg of pelltobarbitalum natricum per kg body weight. The brain tissues of the monkeys were then isolated for immunohistochemical analysis and Western blotting.

### In utero delivery of lentiviruses to the fetal monkey brain.

The method was established in our recent study (38). The pregnancy of monkeys after timed mating was assessed periodically by ultrasound examination, and pregnancy was confirmed with ultrasound evidence of fetal heart activity. Pregnant monkeys having fetal monkeys at E55 or G55 were selected for in utero injection. After induction of anesthesia of the pregnant monkey with intramuscular injection of Zoletil 50 at a dose of 5 mg/kg, a 25-gauge Quincke needle (Becton Dickinson) was used to target the lateral ventricle of the fetal monkey brain closest to the anterior maternal abdomen under continuous ultrasound imaging. One hundred μl lentiviral of parkin or control gRNA mixed with 100 μL lentiviral Cas9 were injected as a slow bolus to deliver a total dose of 1.0 × 10^8^ vg (lentiviral gRNA) and 2.0 × 10^8^ vg (lentiviral Cas9) in each fetus. The correct delivery would result in ventricular swelling, and the needle was removed immediately. The injected fetus was monitored for another 15 minutes. We obtained 2 newborn monkeys, which were delivered naturally. One newborn monkey was euthanized with intramuscular injection of barbiturates with the dose of 100 mg/kg at P1 to isolate its brain for analysis.

### Dephosphorylation of endogenous proteins.

Brain stem tissues (20 mg) from one 8-year old WT monkey were homogenized in 0.5 mL ice-cold homogenization buffer (100 mM NaCl, 50 mM Tris-HCl, 10 mM MgCl_2_, 1 mM dithiothreitol, 0.5% Triton X-100, pH to 7.9) containing protease inhibitors (cOmplete ULTRA EDTA-free tablets, Roche, 05892791001) and with or without phosphatase inhibitors (PhosSTOP, Roche, 04906837001). Ten milligrams of proteins from the brain stem lysates were treated with alkaline phosphatase (1 U/μL, FastAP, Thermo Scientific, EF0654) for 0.5 or 1 hour at 37°C to dephosphorylate endogenous proteins. The samples were then analyzed by Western blotting with 2 antibodies specific to pS65 parkin, anti-phosphorylated S112-Bad, anti-parkin, and anti-Bad.

### Phos-tag gel analysis.

To examine the in vivo phosphorylation levels of endogenous pS65-parkin in WT and *PINK1* mutant (M6) monkey brains, Phos-tag SDS-PAGE analysis was performed according to our previous study (30) by using phos-tag acrylamide from Wako Pure Chemical Industries. Briefly, monkey brain cortical tissues from WT and *PINK1* mutant monkey brains were homogenized with RIPA buffer containing EDTA-free protease inhibitor cocktail supplemented with PhosSTOP (Roche). Proteins were resolved by 8% SDS-PAGE using a gel containing 50 μmol/L phos-tag acrylamide and MnCl_2_ at 85 V. Before blot transfer, gels were incubated twice in SDS-PAGE transfer buffer containing 10 mmol/L EDTA for 20 minutes. Gels were then incubated in SDS-PAGE transfer buffer without EDTA for 10 minutes. Gel transfer and immunoblotting were performed by following a standard procedure.

### Quantification of immunostaining signals.

At least 2 monkeys were analyzed to quantify immunostaining signals, in accordance with the minimum number of monkeys required for biological replication in the study of molecular and pathological changes in primate brains (59). Since stereotaxic injection of AAV was performed to target genes in specific brain regions, the monkey brain regions exhibiting GFP or RFP, which indicates AAV infection, were identified and subsequently sectioned for immunostaining. The brain region was cut into 30 μm sections. A minimum of 5 sections from each animal’s brain were examined. Four to 5 images were captured per section using a Zeiss Axiocam 506 color camera. The number of cells and aggregates labeled by specific antibodies per image (×20 or ×40 magnification) was counted. The data were presented as mean ± SEM for statistical analysis.

We also used digital immunostaining intensity to quantify immunostaining signals of individual cells by adopting the methods described previously (73). Basically, photomicrographs (300 pixels/inch) at a magnification of ×200 or ×400 under a Zeiss Axio Imager A2 microscope were captured by a Zeiss Axiocam 506 color camera and used for quantification by ImageJ. At least 50 cells per group were randomly selected for analysis. The intensity of immunolabeling of cells was measured by selecting the area of each individual cell to obtain the average pixel intensity. The same procedure was applied to obtain the background pixel intensity from an area without cells for each photomicrograph. Thus, the background pixel intensity was subtracted from the pixel intensity of an analyzed cell to obtain the specific labeling intensity of the analyzed cell. This specific labeling intensity reflects the relative values of immunostaining and was used for statistical comparison of different groups.

### Statistics.

Samples from multiple animals (Supplemental Table 1) were used in the current study. When analyzing Western blots, at least 3 independent experiments were conducted, and densitometric results were quantified. For immunocytochemical analysis, the number of cells and aggregates labeled by specific antibodies per image (×20 or ×40) from at least 3 independent experiments were quantified. Statistical significance was assessed using the 2-tailed Student’s *t* test for comparing 2 groups, and a *P* value of less than 0.05 was considered significant. When analyzing 3 or more sets, we used a 1-way ANOVA test to determine statistical significance. Data presented in figures represent the mean ± SEM. Calculations were performed using GraphPad Prism software, version 8.0.

### Study approval.

Postmortem tissues from 2 individuals were used for Western blotting analysis. The use of postmortem human tissues in the study was approved by the Institutional Ethics Committee of Central South University on March 10, 2021 (approval no. 2021-KT20), in compliance with the Code of Ethics of the World Medical Association (Declaration of Helsinki). All animal procedures and experiments on monkeys were approved by the IACUCs at Yuanxi Biotech Inc. and Guangdong Landao Biotechnology Co. Ltd. and were conducted according to the committees’ guidelines to ensure the safety of personnel and animal welfare. Mouse models used in this study were housed at the animal facility at Jinan University. All procedures involving mouse models were conducted in accordance with the Animal Care and Use Committee of Jinan University. This study occurred in strict compliance with the *Guide for the Care and Use of Laboratory Animals of the Institute of Laboratory Animal Science* (est. 2006) and *The Use of Nonhuman Primates in Research of the Institute of Laboratory Animal Science* (est. 2006) to ensure the safety of personnel and animal welfare.

### Data availability.

All data in this manuscript are available. All materials are available upon request. Values for all data points in graphs are reported in the Supporting Data Values file.

## Author contributions

WY, SL, and XJL designed and supervised the research experiments. WY, RH, QW, XX, XC, ZW, FZ, CC, TX, LC, Yanting Liu, DH, and MP performed molecular biology experiments and pathological studies. XC, ZT, XG, WY, PY, and BL performed animal experiments. Yunbo Liu provided technical help and advice for embryonic gene targeting in the initial study. FH and PW provided animal facilities for the establishment of the monkey model. XY provided human postmortem tissues. WY and XJL wrote the manuscript. Research using monkey models requires a long experimental period and substantial workload, necessitating extensive teamwork. RH, QW, XX and XC contributed substantially to this work, warranting co–first authorship. Relative contributions are reflected in authorship order. RH performed immunohistochemical staining and Western blotting to analyze pathological changes in the monkey and human postmortem brains, as well as the corresponding statistical analyses. QW conducted biochemical experiments including Western blotting, and participated in monkey behavioral and pathological examination. XX was responsible for viral vector construction, in vitro assays, and some immunohistochemical staining experiments. XC constructed various plasmids, assisted with monkey brain injections, and established primarycell cultures from monkey brains.

## Supplementary Material

Supplemental data

Unedited blot and gel images

Supporting data values

## Version 1 10/15/2024

Electronic publication
