# Supplementary material for: Deficiency of parkin causes neurodegeneration and accumulation of pathological α-synuclein in monkey models
Source: J Clin Invest. 2024 Oct 15;134(20):e179633. doi: 10.1172/JCI179633 (PMC11473153; doi:10.1172/JCI179633)
Supplement: Supplemental data [file jci-134-179633-s078.pdf]

Supplementary Table 1 Monkeys used in the study

**Name**	**Targeted gene**	**Sex**	**Death and age**
Park-1	*Parkin* KD (germline)	M	Gestation day 102 day (aborted)
Park-2	*Parkin* KD (germline)	F	Gestation day 91 day (aborted)
Park-3	*Parkin* KD (germline)	M	1.5 yr (sudden death)
Park-4	*Parkin* KD (germline)	M	3 yr (euthanized)
M6	*PINK1* KD (germline)	F	3 yr (euthanized)
WT-1	-	M	Gestation day100 day (aborted)
WT-2	-	F	Gestation day125 (aborted)
WT-3	-	M	Gestation day130 (aborted)
WT-4	-	M	3 yr (euthanized)
WT-5	-	M	3 yr (euthanized)
WT-6	-	M	3 yr (euthanized)
WT-7	-	F	1.5 yr (euthanized)
WT-8	*Parkin* KD (adult)	M	6 yr (AAV injection then euthanized)
WT-9	*Parkin* KD (adult)	M	7 yr (AAV injection then euthanized)
WT-10	*Parkin* KD (adult)	M	9 yr (AAV injection then euthanized)
WT-11	*Parkin* KD (adult)	M	12 yr (AAV injection then euthanized)
WT-12	*Parkin* KD (adult)	F	25 yr (AAV injection then euthanized)
WT-13	*Parkin* KD (adult)	F	22 yr (AAV injection then euthanized)
WT-14	*Parkin* KD (adult)	M	22 yr (AAV injection then euthanized)
WT-15	*PINK1* KD (adult)	M	10 yr (AAV injection then euthanized)
WT-16	*PINK1* KD (adult)	M	22 yr (AAV injection then euthanized)
WT-17	*AAV control and PINK1* OE	F	25 yr (AAV injection then euthanized)

WT-18	*Parkin* OE (adult)	M	25 yr (AAV injection then euthanized)
WT-19	*Parkin* OE (adult)	F	22 yr (AAV injection then euthanized)
WT-20	*AAV control* *and PINK1* OE	F	25 yr (AAV injection then euthanized)
WT-21	-	M	25 yr (euthanized as wild type)
WT-22	-	M	8 yr (euthanized as wild type)
WT-23	-	M	8 yr (euthanized as wild type and brain slice culture)
WT-24	-	M	9 yr (euthanized as wild type and brain slice culture)
WT-25	*Parkin* KD (adult)	M	8 yr (AAV injection then euthanized)
WT-26	*Parkin* KD (adult)	M	8 yr (AAV injection then DOPA imaging and euthanized)
WT-27	*Parkin* KD (adult)	M	7 yr (AAV injection then euthanized)
WT-28	-	F	26 yr (euthanized as wild type)

Supplementary Table 2 Antibodies and reagents used in the study

REAGENT or RESOURCE	SOURCE	IDENTIFIER	Dilution
**Antibodies**			
Rabbit Polyclonal Anti-Alpha Synuclein	Proteintech	10842-1-AP	1:1000(ICC)
Rabbit polyclonal anti-Alpha-synuclein S129	Abcam	Ab59264	1:500(IHC)
Mouse monoclonal Anti-β-actin	Santa Cruz	Cat# 47778	
Rabbit polyclonal anti-Beclin1	Abcam	Cat# ab55878	
Rabbit polyclonal anti-Caspase 3	Cell signaling	Cat# 9665	
Monoclonal Anti-CRMP2	Cell signaling	Cat# 9393	
Rabbit monoclonal anti-HA-Tag (C29F4)	Cell signaling	Cat#3724	1:500(ICC)
Rabbit polyclonal anti-HSP70	Cell signaling	Cat# 4876	
Rabbit polyclonal anti-HSP60	Abcam	Cat# ab46798	
Rabbit polyclonal anti-DJ-1	Abcam	Cat# ab18257	1:1000

Anti-20s β5	Santa cruz	sc-393931	1:2000
Anti-Flag	Sigma	F1804	1:1000
Mouse monoclonal anti-GFAP	Millipore	Cat# MAB360	
Rabbit polyclonal anti-GFAP	Abcam	Cat#ab7260	1:2000(ICC)
Mouse monoclonal anti-GAPDH	Proteintech	60004-1-Ig	
Rabbit polyclonal anti-Drp1	NOVUS	NB110-55288	
Rabbit polyclonal anti-LC3B	Novus biologicals	Cat# NB100-2220	
Rabbit polyclonal anti-mTOR	Cell signaling	Cat #2983	
Rabbit polyclonal anti-Mfn1	Cell signaling	Cat# 14739	
Mouse monoclonal anti-NeuN	Millipore	Cat# MAB377	
Rabbit monoclonal anti-NeuN	Abcam	Cat# ab177487	1:1000(ICC)
Rabbit polyclonal anti-OPA1	Cell signaling	Cat# 80471	
Mouse monoclonal anti-8-OHdG(15A3)	Santa Cruz	sc-66036	1:500(ICC)
Rabbit polyclonal anti-Parkin (phosopho S65)	CST	36866S	1:500
Rabbit polyclonal anti-Parkin (phosopho S65)	Affinity	AF3500	1:1000
Mouse monoclonal anti-Parkin	Santa cruz	sc-32282	1:1000(WB)
Mouse monoclonal anti-Parkin	Millipore	MAB5512	1:500(ICC)
Rabbit polyclonal anti-PINK1	Novus biologicals	Cat# BC100-494	
Rabbit monoclonal anti-Phospho-Histone H2A.X	Cell signaling	Cat#9718	1:500(ICC)
Mouse monoclonal anti-PSD95	ThermoFisher	MA1-045	
Rabbit polyclonal anti-RFP	Rockland	Cat#600-401-379	
Rabbit anti-SNAP25	Cell signaling	Cat# 5308	
Rabbit polyclonal anti-synapsin-1	Cell signaling	Cat# 5297	
Mouse monoclonal anti-γ-tubulin	Sigma	Cat# T6557	
Rabbit polyclonal anti-Tom20	Santa cruz	SC17764	
Rabbit Monoclonal anti-Tom20	Cell signaling	Cat# 42406	1:500(ICC)
Rabbit polyclonal anti-Tyrosine Hydroxylase (TH)	Abcam	Ab112	1:1000(ICC)
Mouse monoclonal anti-Ubiquitin(P4D1)	Cell signaling	Cat#3936	1:1000(ICC, WB)
Rabbit polyclonal anti-VDAC1	Abcam	Ab14734	1:2000
Rabbit monoclonal anti-Vinculin	Abcam	Cat# ab129002	1:3000
Donkey Anti-Mouse	Jackson Immunolabs	Cat#715-035-151	
Donkey Anti-Rabbit	Jackson Immunolabs	Cat#715-035-152	
Mouse and Rabbit specific HRP/DAB (ABC) Detection IHC Kit	Abcam	Ab64264	
Canada balsam	Solarbio	C8300	
Fluoro-Jade® C RTDTM Stain Reagent	Biosensis	Cat#TR-100-FJ	
QIAamp DNA Mini kit	QIAGEN	Lot.166047921	

Oligonucleotides

Monkey *PINK1*-Exon2 sgRNA	Thermo Fisher, Guangzhou,China	GGCTGGAGGAGTATCT GATAGGG

Monkey *PINK1*-Exon4 sgRNA	Thermo Fisher, Guangzhou,China	CCGGGTTCT CCGCGCTTT CACC
T7-sgRNA Sense Primer	Thermo Fisher, Guangzhou,China	GAAATTAATACGACTC ACTATA
T7-sgRNA Anti-sense Primer	Thermo Fisher, Guangzhou,China	AAAAAAAGCACCGACT CGGTGCCAC
Mouse and Monkey β-actin primer-F	Thermo Fisher, Guangzhou,China	GAAGATCAAGATCATT GCTCCTC
Mouse and Monkey β-actin primer-R	Thermo Fisher, Guangzhou,China	CTGCTTGCTGATCCAC ATCTGCTG
Mouse *Parkin* Exon3-6 genotyped primer-1F	Thermo Fisher, Guangzhou,China	AGCCAGATCCTGGCTC TGTTAGAA
Mouse *Parkin* Exon3-6 genotyped primer-1R	Thermo Fisher, Guangzhou,China	TTGGCAGTCTGGGCTT CTGAG
Mouse *Parkin* Exon3-6 genotyped primer-2F	Thermo Fisher, Guangzhou,China	GCCACCCACATTCCTA GTCTACAC
Mouse *Parkin* Exon3-6 genotyped primer-2R	Thermo Fisher, Guangzhou,China	AGGAAGGGATCTGGA GAGCTAGG
Monkey *Parkin*-Exon2 primer-1F	Thermo Fisher, Guangzhou,China	GCCTCTGTTTCTCAGG ATGG
Monkey *Parkin*-Exon2 primer-1R	Thermo Fisher, Guangzhou,China	TCCAATCTTTCCTGCTT GCT
Monkey *Parkin*-Exon2 primer-2F	Thermo Fisher, Guangzhou,China	GGGCAACTCTGTTTTT CACAA
Monkey *Parkin*-Exon2 primer-2R	Thermo Fisher, Guangzhou,China	TCCTGCTTGCTGTTTTA ATGC
Monkey *Parkin*-Exon3 primer-1F	Thermo Fisher, Guangzhou,China	GCCCCAGTTCAGTGTT GTTTG
Monkey *Parkin*-Exon3 primer-1R	Thermo Fisher, Guangzhou,China	CAGTGATGTCTCCTTG GAGTG
Monkey *Parkin*-Exon3 primer-2F	Thermo Fisher, Guangzhou,China	CATGTGCCTAAGCTGG TCAA
Monkey *Parkin*-Exon3 primer-2R	Thermo Fisher, Guangzhou,China	CCATGGAGACTGCACT AAACAA
Monkey *ACTB* primer-F	Thermo Fisher, Guangzhou,China	TCGTGCGTGACATTAA GGAG
Monkey *ACTB* primer-R	Thermo Fisher, Guangzhou,China	CAGGAAGGAAGGTTG GAAGA
**Recombinant DNA**		
Plasmid	Cas9-MLM3613	
Plasmid	sgRNA with T7 promoter	
Plasmid	pAAV-U6-sgRNA (PX552)	
Plasmid	pAAV-spCAS9 (PX551)	

HEK293 cell line was obtained from ATCC (Cat#CRL-11268).

Ovarian stimulation and recovery of monkey oocytes. The methods for monkey ovarian stimulation and oocyte recovery are similar to those described in our previous studies (Tu, et al., 2017; Yang et al., 2019). Regular cycling females arranging between 5-8 years of age were subjected to follicular stimulation using twice-daily intramuscular injections of 18 IU of recombinant human FSH (rhFSH) for 8 days, followed by 1000 IU of human chorionic gonadotropin (HCG) on day 9. Cumulus-oocyte complexes were isolated by surgery operation and aspiration 37 h post-rhCG. Follicular contents were placed in Hepes-buffered Tyrode's albumin lactate pyruvate medium (TALP-Hepes) containing 0.3% BSA at 37℃, supplemented with 5 IU ml^{-1} of heparin (Sigma, Inc.). Oocytes were stripped of cumulus cells with pipetting for 45-60 sec, then filtered through a 70 μm cell strainer, and collected in a 60 mm petri dish containing 5-7 ml of TALP-Hepes. Oocytes were collected under a dissecting microscope to separate GV (intact germinal vesicle), metaphase I (GVB, no germinal vesicle, no polar body), metaphase II (MII, first polar body present) from other dead oocytes. Oocytes were rinsed and then transferred to 50 μl pre-equilibrated maturation medium containing Connaught Medical Research Laboratories medium 1066 (CMRL-1066; Invitrogen Inc.) supplemented with 10% heat-inactivated fetal bovine serum (FBS), 40 μg ml^{-1} sodium pyruvate, 150 μg ml^{-1} glutamine, and 550 μg ml^{-1} calcium lactate under mineral oil. Immature oocytes such as GVB or GV cells were cultured in a 50 μl TALP-Hepes under 6% CO_2 at 37.5℃ for up to 24 h.

Male macaques were electro-ejaculated with a current isolation stimulator (JL-C4 V2a, JIALONG, China) equipped with electrocardiographic pad electrodes for direct penile stimulation (30–50 V 20-msec duration, 18 pulses/sec). Semen samples were collected into 15-ml tubes. Ejaculated sperm were diluted to 2×10^5 in 10% polyvinylpyrrolidone (PVP) to reduce motility and placed in a separate drop on the manipulation dish. A single sperm aspirated from the sperm drop into the injection needle, was transferred to the oocytes in the TALP-Hepes drop. MII oocytes

immobilized with a holding pipet on the polar body at the 6 o'clock or 12 o'clock position, and then injected with a sperm through a needle through the zona into the cytoplasm (ICSI). After ICSI, oocyte was washed twice in Hamster Embryo Culture Medium 9 (HECM-9) before being transferred into a pre-equilibrated 50 μl drop of HECM-9, covered with mineral oil and incubated at 37.5℃ with 6% CO_2 for 8-10 h. Oocytes with a second polar body and two pronuclei arising after ICSI were confirmed successful fertilization. Zygotes were injected with Cas9 mRNA (200 ng l^{-1}) and gRNAs (50 ng l^{-1}), and the injected zygotes were cultured for embryo development. Embryos at 4-8 cell stages were used for transfer or divided into single blastomere for PCR. The pronuclear formation was recorded 16–20 h post-ICSI, and the progression of embryo growth was recorded daily.

Cas9/sgRNA vectors for embryo injection. The Cas9/sgRNA vectors were also described in our early studies (Yang et al., 2019)**.** Briefly, Cas9 plasmid (MLM3613, Plasmid #42251) was used to express spCas9 nuclease (Streptococcus pyogenes) under the control of the CMV or T7 promoter. Dr. Liangxue Lai at The Guangzhou Institutes of Biomedicine and Health, Chinese Academy of Sciences, Guangzhou, China provided the p-U6-gRNA and p-T7-gRNA expression vectors, which were used separately for HEK293 transfection and in vitro transcription of gRNAs. gRNAs were designed based on the targeted sequences in the monkey *Parkin* genes and were generated by inserting gRNAs into p-U6-gRNA and p-T7-gRNA via BbsI restriction sites. gRNA sequences are as follows: *Parkin* exon 2 sgRNA: 5'- CTCCAGCCATGGTTTCCCAGtgg-3', *Parkin* exon 3 sgRNA: 5'- CAAGAAATGAATGCAACTGGagg-3'. Template DNAs for *in vitro* transcription were generated by PCR amplification of the gRNAs plasmids via the following primers: Sense (5'-GAAATTAATACGACTCACTATA-3'), Anti-sense (5'-AAAAAAAGCACCGACTCGGTGCCAC-3'). The PCR products were purified and transcribed *in vitro* by mMESSAGE mMACHINE T7 kit (Ambion, AM1344). Cas9 plasmids were linearized by PmeI and *in vitro* transcribed using MAXIscript T7 (Ambion, AM1312). The synthesized transcripts were added with poly-A using the

E. coli Poly (A) Polymerase kit (NEB, M0276) and were purified using LiCl with an additional ethanol precipitation.

Culture of monkey primary cortical glia cells. Cortical tissues were isolated from a fetal monkey brain at embryonic day 60 using the method described in our previous studies (Yang et al., 2022). Dissected tissues were treated with 0.0625 mg/ml trypsin in 1× HBSS buffer without calcium or magnesium for 10 min at 37°C, followed by triturating with a 1 ml pipette tip 20 times. Cells were then washed once with the tissue culture medium and centrifuged at 250 × *g* for 5 min. For culturing glia cells, cells were grown in DMEM medium with 10% FBS at a density of 10^5/mL. Transfection of cultured glia cells via electroporation was performed using Nucleofector Kit (Lonza) before plating primary cells on poly-D-lysine coated 12-well plates. In brief, 3 × 10^6 cells were used for electroporation with 50 pmol siRNA (control siRNA or *PINK1* siRNA). The cultured cells were collected for western blotting 72 hours after siRNA transfection. The sequences of siRNAs are listed as follows: *PINK1*, 5'-GCAAAUGUGCUUCAUCUAAGC-3', scramble, 5'-CCUAAGGUUAAGUCGCCCUCG-3'.

Analysis of insoluble proteins in monkey brain tissues. The sequential extraction of proteins from brain tissues was performed as described previously with minor modifications (Tokarew et al., 2021). Approximately 150 mg of the brain cortical tissues from 8-y- and 25-y-old monkeys were weighed and placed in 2 ml Tris buffer (5 mM Tris, 140 mM NaCl pH 7.5) containing complete EDTA-free protease inhibitor cocktail. The samples were homogenized on ice in a Dounce glass homogenizer by 60 passes, and then spun at 163,202 × g for 30 min at 4℃. The soluble supernatant was transferred to a fresh tube and the pellet including insoluble proteins was resuspended in 2 ml SDS buffer (5 mM Tris, 140 mM NaCl pH 7.5, 2% Triton X-100, 2% SDS). The resuspended samples were incubated on ice for 10 min and centrifuged again at 163,202 × g

for 30 min at 4℃. Extracted proteins from the SDS buffer were run on SDS PAGE, and membranes were immunoblotted for the detection of Parkin, pS65-Parkin and TH.

PCR analysis of targeted embryos and single blastomere. Monkey embryos injected with Cas9/gRNA complex were collected and lysed (50 mM Tri-HCL (pH 8.5), 1 mM EDTA (pH 8.0), 0.5% tween20, 20 mg l-1proteinaseK) for PCR. The *Parkin* DNA including the target sites were isolated by two rounds of PCR with nested primers for each round see table 1. The first round PCR was performed by initial incubation at 95°C for 5 min, followed by 35 cycles at 95°C for 30 s, 62°C for 45 s, and 72°C for 45 s. By using the products of the first round PCR as the templates, the second round PCR was performed by initial incubation at 95°C for 5 min, followed by 35 cycles of 95°C for 30 s, 62°C for 30 s, and 72°C for 30 s. T7E1 assay was used to analyze the PCR products to detect the targeted DNA mutations. PCR products corresponding to genomic modifications were then subcloned into a TA-cloning vector for sequencing to verify the mutation sequences.

Immunofluorescent staining and immunohistochemistry. For immunofluorescent staining, monkey brain tissues were sliced as slabs up to about a centimeter thick, then they were post-fixed in 4% PFA (paraformaldehyde) in 0.01 mol/L PBS at 4℃ for 5 days. After that, they were cryopreserved in 15% sucrose in 0.01 mol/L PBS at 4 °C for 3 days. When the tissues sink to the bottom, they were transferred into 30% sucrose at 4 °C for 10 days to let the brain completely sink to the bottom of the tube. 30 μm-thick coronal sections were cut using a cryotome, incubated first in a blocking solution (2% normal goat serum, 3% BSA, 0.3% Triton X-100 in PBS), then with primary antibodies to relative proteins for 18 h at 4 °C, and next with secondary antibodies and Hoechst diluted with 0.3% Triton X-100 in PBS. To remove the interference caused by lipofuscin, 0.3% Sudan black (in 70% ethanol diluted by 1xPBS) were used to incubate the slices for 3 min, and

next with 0.3% Triton X-100 in PBS to wash the slices. Double immunofluorescence staining was analyzed using a confocal imaging system (Olympus FV3000 Microscope).

FJC staining to detect neuronal degeneration was performed according to the manufacturer's protocol (Fluoro-Jade C staining Kit, Biosensis) and stained sections were imaged by IF microscopy.

For immunohistochemistry, Mouse and Rabbit specific HRP/DAB (ABC) Detection IHC Kit (Ab64264) was used to perform DAB staining. Images of DAB staining are captured on a Zeiss Axio Imager A2 microscope.

For Golgi staining, monkey brain tissues were sliced as slabs up to about a centimeter thick for staining with the FD Rapid GolgiStain Kit (FD NeuroTechnologies, PK401). Briefly, the brain slices were rinsed with double distilled water and then immersed in FD Solution A/B (1:1) for 2 weeks at room temperature in the dark. The brain slices were then transferred to FD Solution C for 72 h incubation at room temperature. For freezing treatment, the brain slices were placed in a plastic spoon and slowly dipped into isopentane that had been precooled with dry ice. After the tissue was completely immersed in isopentane, it was placed on dry ice for another minute to ensure that the tissue was well frozen. Coronal sections of 140 μm thickness were cut using a cryostat (Thermo NX50) at –22°C and then transferred to gelatin coated slides using Solution (D/E) for staining as soon as possible after drying overnight at room temperature.

MRI image acquisition. MRI scanning was conducted as described in our previous work (Li et al., 2023). Briefly, the monkeys were anesthetized by ketamine (10 mg/kg, i.m.) and placed into the scanner in the prone position for T1-weighted 3-dimensional MR scan. The scans were collected on a 3.0 T MR machine (Siemens MAGNETOM 3T Prisma) using a custom-designed 8-channel radio-frequency surface head coil at Guangzhou Women and children's medical center. The whole brain images were acquired with a 3D Bravo T1 sequence (TR = 5000 ms, TE = 2.31 ms, slice thickness = 0.3 mm, matrix size = 120 ×120, FOV = 12 × 12 cm), a T2 space dark sequence (TR = 3500 ms, TE = 178 ms, slice thickness = 0.3, matrix size = 120× 120, FOV = 12 × 12 cm),

and a T2 FLAIR sequence (TR = 6500 ms, TE = 184 ms, slice thick-ness = 0.5 mm, matrix size = 120 × 120, FOV = 12 ×12cm).

PET/CT imaging and data analysis. PET-CT scanning was conducted in PET/CT-MRI Center, the First Affiliated Hospital of Jinan University (JNUH) as described in our previous work (Tu et al., 2019; Nie et al., 2019; Tu et al., 2023). The PET tracer [18F]DOPA was radiolabeled in the Center of Cyclotron and PET Radiopharmaceuticals (CCPR) at JNUH. Briefly, animals were deprived of food for 12 h before tracer injection. The mean dose of [18F]DOPA administered was 0.5 mCi/kg body weight (18.5 MBq/kg. The animal was placed in the imaging chamber, anesthetized with 2.0% isoflurane/oxygen gas mixture throughout the imaging experiment. Body temperature was maintained at 37°C and monitored during anesthesia. Head position was fixed with a stereotactic frame. CT scan was performed first, followed by 10-minute static positron emission data collection at 60 min post-injection. CT data were acquired in breath-hold with 140 kV, 230 mA modulated using the GE AutomA technique (GE Medical System, Milwaukee, USA) with a noise index of 30, slice thickness of 3.75 mm, slice interval of 3.27 mm, matrix size of 256 × 256 and scan FOV of 70 cm. Data analysis was performed using PMOD4.1 (Pmod Technologies LLC, Zürich, Switzerland). The uptake of [18F]DOPA in occipital cortex was used as reference for SUVr analysis as previously described (Xiao Z, Acta Pharm Sin B. 2022). The co-registration of PET image to individual MR image, which was transformed into brain template MR images, was carried out following published protocols (McLaren et al., 2009; Tu et al., 2019). The bilateral striatum was analyzed, and the volume of interest (VOI) was drawn and resampled into the PET space to extract SUV by applying Carimas software (Turku, Finland) and PMOD software (Zürich, Switzerland). SUV was corrected by initial injection dose and body weight of monkeys. Normalization of PET imaging analysis was conducted by SUVRR/L (SUV of the right striatum/SUV of the left striatum).

Generation of plasmids. cDNAs encoding tagged human PINK1 (PINK1-Flag) and human Parkin were cloned into AAV vectors containing the CMV promoter. For the construction of PINK1 M1A and Parkin S65A mutants, we mutated the first methionine to alanine in the WT-PINK1-FLAG vector and changed the 65th amino acid serine to alanine in the WT-Parkin vector using the QuikChange II site-directed mutagenesis kit (Agilent).

References

Li B. et al. CHD8 mutations increase gliogenesis to enlarge brain size in the nonhuman primate. Cell Discov 9, 27, doi:10.1038/s41421-023-00525-3 (2023).

McLaren DG, et al. A population-average MRI-based atlas collection of the rhesus macaque. Neuroimage. 2009;45(1):52-9.

Nie B, et al. A population stereotaxic positron emission tomography brain template for the macaque and its application to ischemic model. Neuroimage. 2019;203:116163.

Tokarew JM, et al. Age-associated insolubility of parkin in human midbrain is linked to redox balance and sequestration of reactive dopamine metabolites. Acta Neuropathol. 2021;141(5):725-54

Tu Z. et al. Promoting Cas9 degradation reduces mosaic mutations in non-human primate embryos. Sci Rep 7, 42081, doi:10.1038/srep42081 (2017).

Tu Z, et al. CRISPR/Cas9-mediated disruption of SHANK3 in monkey leads to drug-treatable autism-like symptoms. Hum Mol Genet. 2019;28(4):561-71.

Tu Z, et al. Tauopathy promotes spinal cord-dependent production of toxic amyloid-beta in transgenic monkeys. Signal Transduct Target Ther. 2023;8(1):358.

Yang, W. et al. CRISPR/Cas9-mediated PINK1 deletion leads to neurodegeneration in rhesus monkeys. Cell Res 29, 334-336, doi:10.1038/s41422-019-0142-y (2019).

Yang W. et al. PINK1 kinase dysfunction triggers neurodegeneration in the primate brain without impacting mitochondrial homeostasis. Protein Cell 13, 26-46, doi:10.1007/s13238-021-00888-x (2022).

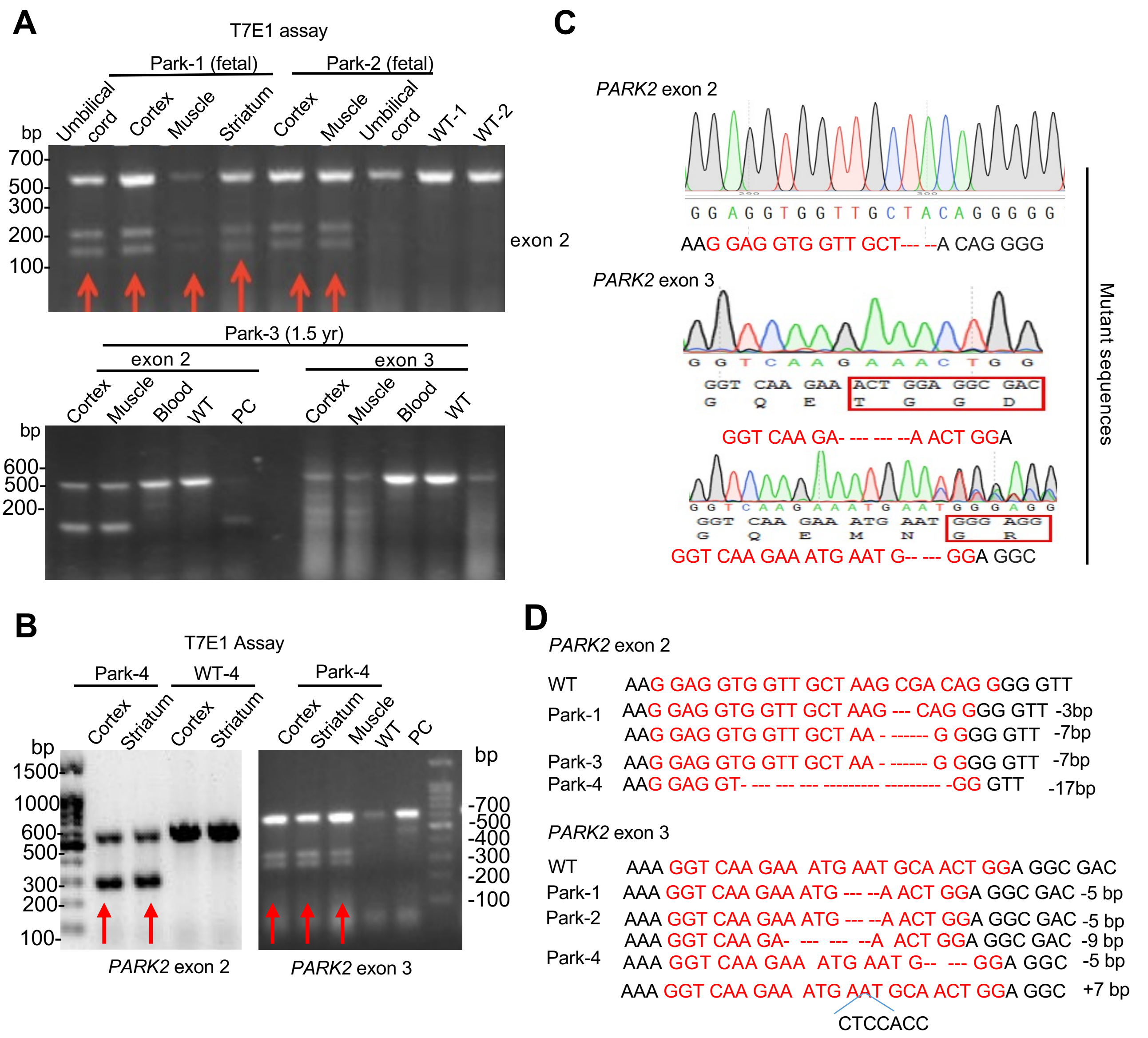

Supplemental Figure 1. *PARK2* targeting by CRISPR/Cas9 in monkeys. (A and **B)** T7E1 analysis of targeted *PARK2* DNAs in fetal monkey and Park-3 (in A), and Park-4 (in B) monkey tissues. PC: positive control. (**C** and **D)** DNA sequencing verified mutations in the targeted exon 2 and exon 3 of the monkey *PARK2* gene.

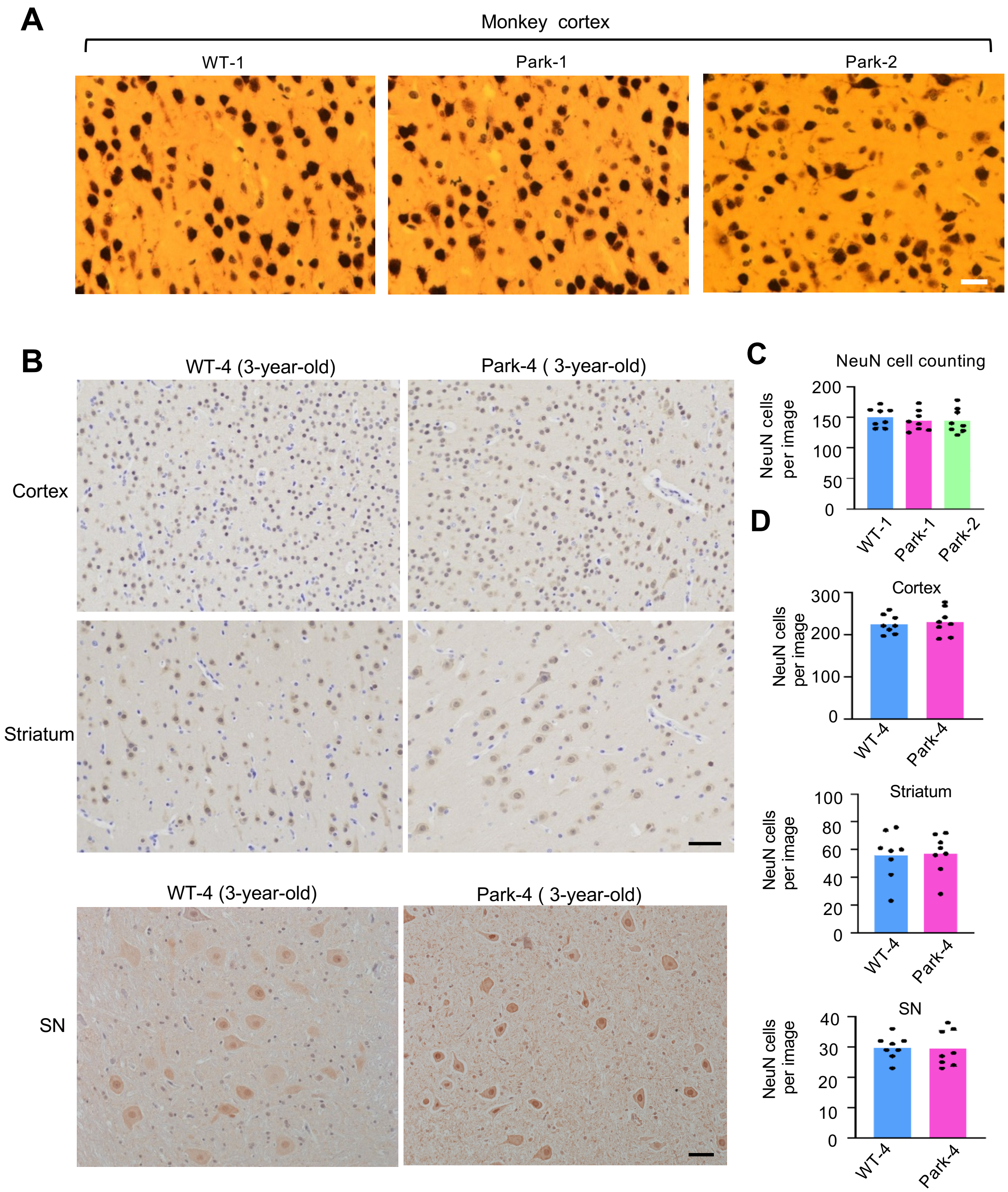

Supplemental Figure 2. ***PARK2*** **targeting did not cause neurodegeneration in the brain of young monkeys. (A** and **B)** Representative immunostaining images of neuronal cells with anti-NeuN in the cortex of fetal monkeys (WT-1, Park-1, and Park-2) (in A) and the cortex, striatum, and SN of 3-y-old WT-4 and Park-4 monkeys (in B). (**C** and **D)** Quantification of the numbers of NeuN positive cells in fetal monkey brains (in C) and 3-year-old monkeys (in D). Data shown as mean +/- SEM; two-tailed Student's t-test; n= different fields (10 x) for counting 900-1250 cells in the brain cortex and 150-300 cells in the substantia nigra and striatum. Scale bars: 20 μm (in A) ; 50 μm (in B).

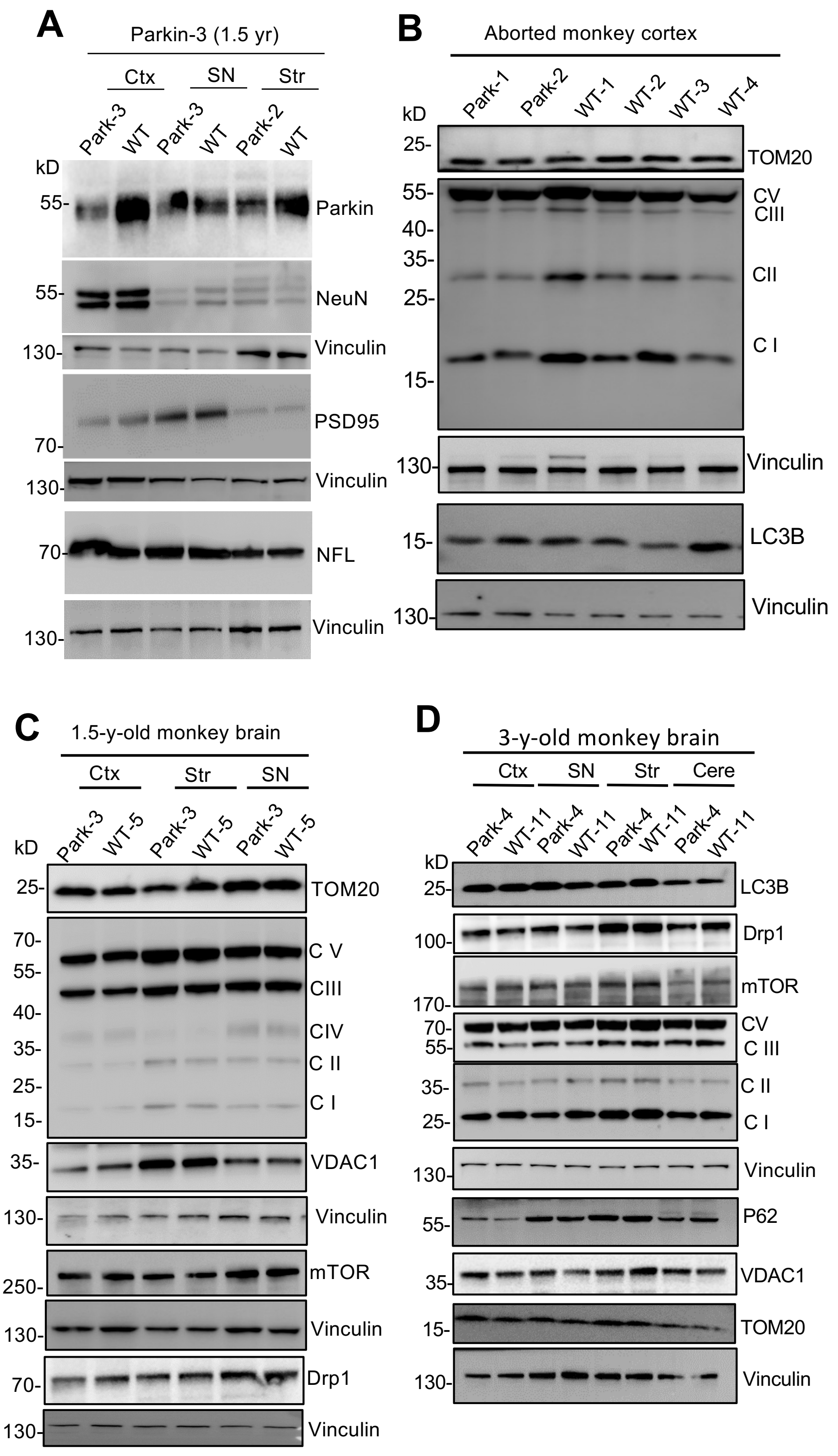

Supplemental Figure 3. *PARK2* targeting did not alter mitochondrial protein expression and function. (A) Western blotting showing a reduction in Parkin levels in the brain tissues (Ctx: cortex, Str: striatum, SN: substantia nigra, Cere: cerebellum) of Park-3 monkey, without alterations in neuronal proteins (NeuN, PSD95) when compared with age-matched WT monkeys. **(B, C** and **D)** Western blotting analysis of the brain tissues of fetal monkeys (B), 1.5-year-old monkeys (C) and 3-year-old monkeys (D) showing no obvious alterations of mitochondrial proteins in *PARK2*-targeted monkey brain regions (Ctx: cortex, Str: striatum, SN: substantia nigra, Cere: cerebellum). Representative western blot results of multiple technical replicates are presented.

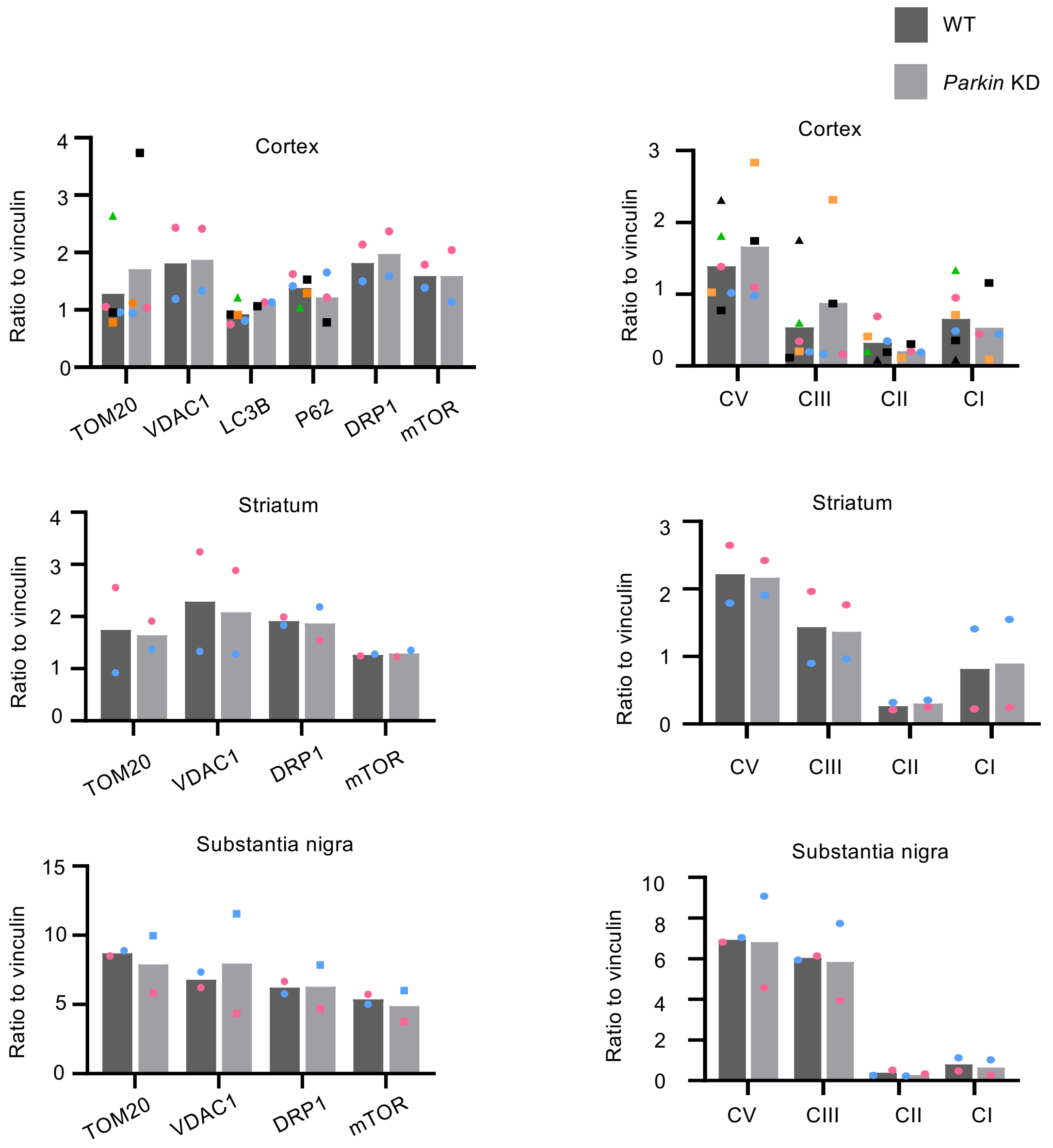

Supplemental Figure 4. Quantitative analysis of the ratios of mitochondrial related proteins to the loading control of Supplemental Figure 3 A-B. The results (mean+/-SEM) were obtained from 3 independent experiments, and no significant differences are found between WT and Parkin KD.

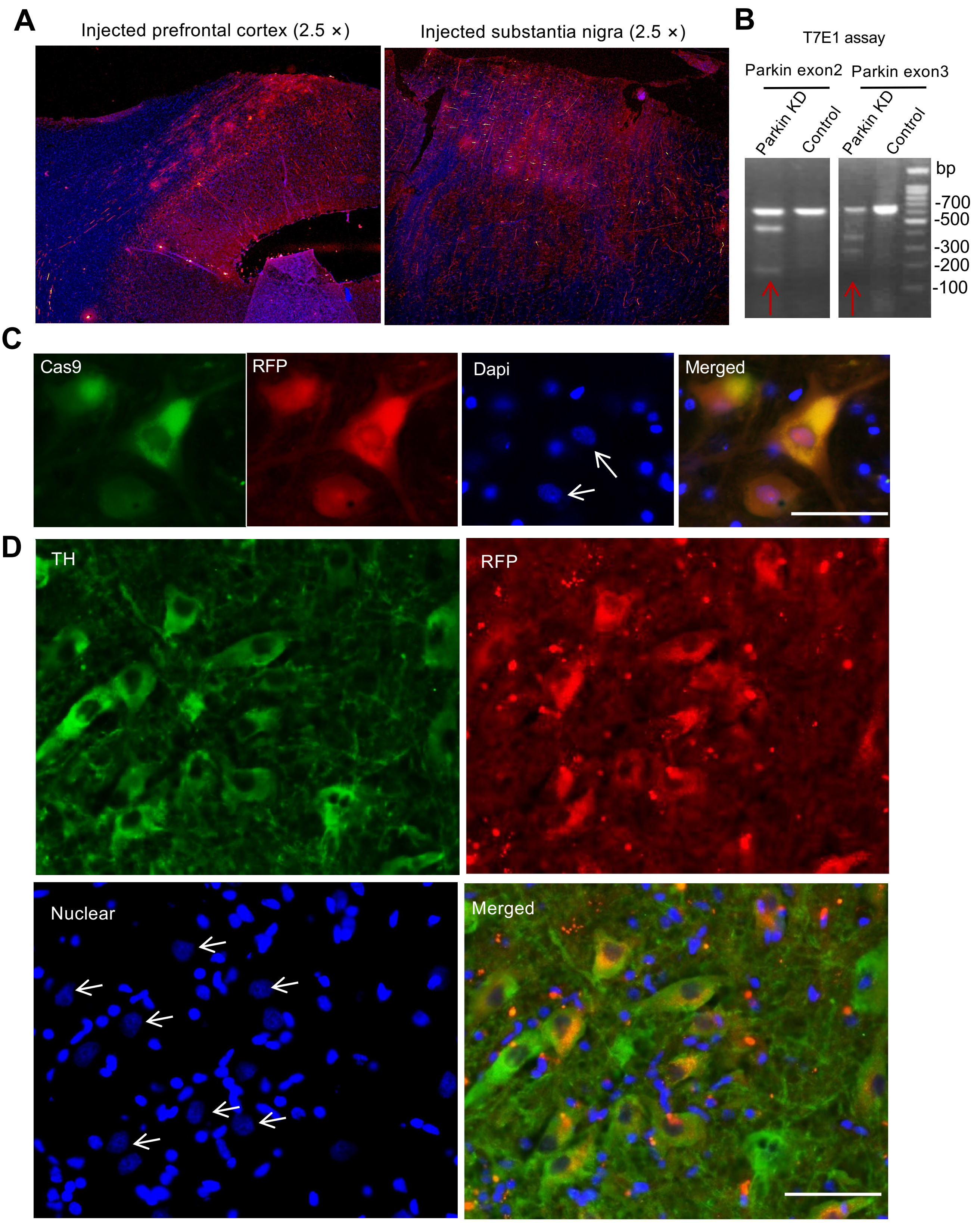

Supplemental Figure 5. Stereotaxic injection of AAV9-CRISPR/Cas9 in the monkey brain leads to the reduced expression of Parkin. (A) The AAV9-CRISPR/Cas9-injected area showing the expression of RFP that is co-expressed by the AAV-gRNA vector. **(B)**T7E1 analysis of *PARK2* DNAs in the monkey brain injected with AAV9 control gRNA or Parkin gRNA/Cas9 (KD). **(C)** Cas9 is co-expressed with AAV9 gRNA-RFP in the injected monkey SN. **(D)** Immunocytochemical staining of the injected monkey's substantia nigra shows that the majority of TH-positive cells were infected by AAV-control gRNA/RFP. Arrows indicate the nuclei of the large-sized neurons, which are less labeled by DAPI compared to those of other cells in the SN. Scale bars: 50 μm. Representative results of multiple technical replicates are presented.

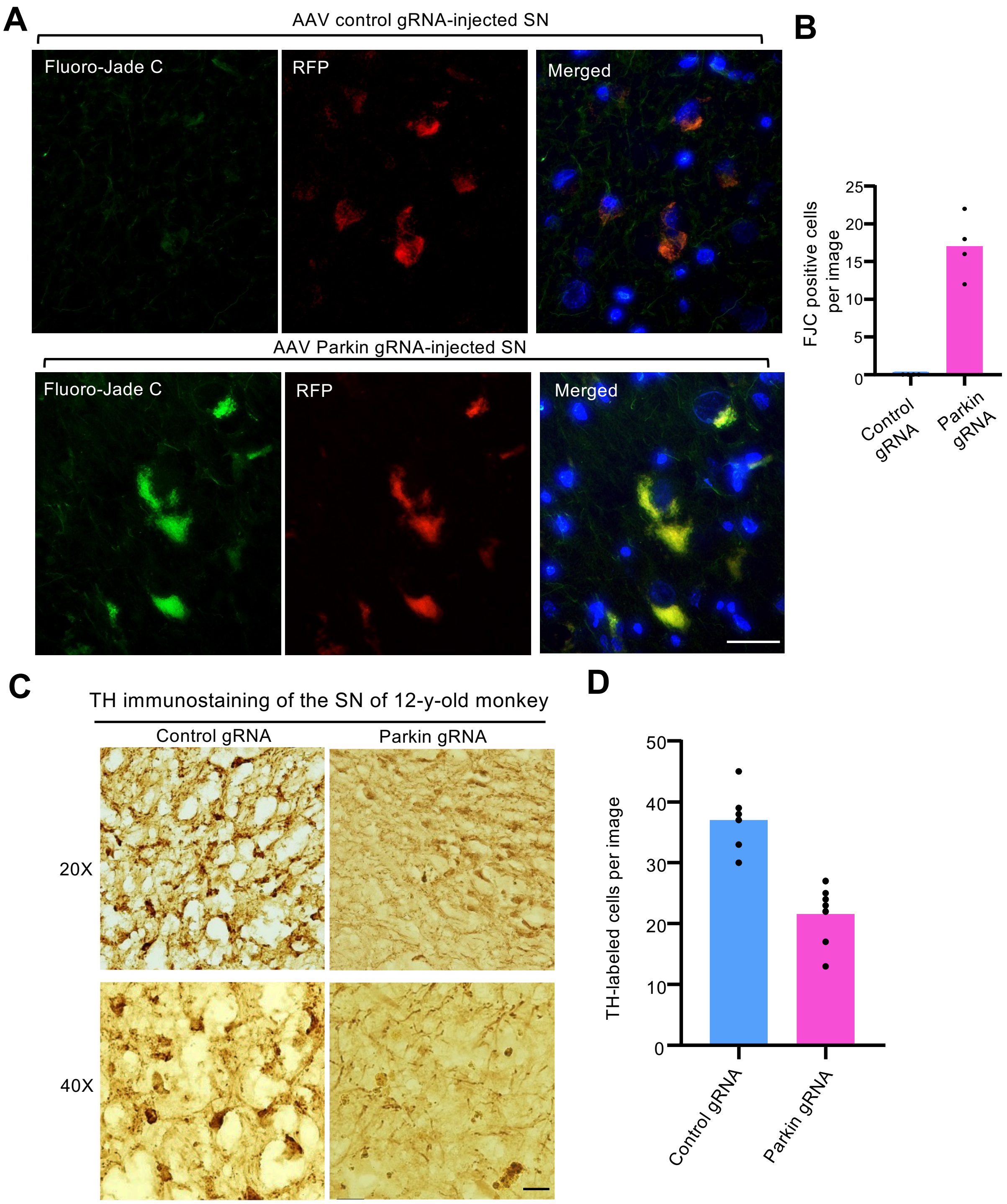

Supplemental Figure 6. Degeneration of the SN neurons in *PARK2*-targeted monkey brains. **(A)** Fluoro-Jad C staining of the SN revealing the degneration of SN neurons in the *Parkin*-targeted monkey brains. Scale bar: 50 μm. **(B)** Quantification of FJC positive cells per image (40 ×). **(C)** TH labeling of the SN of control gRNA and Parkin gRNA targeted 12-y-old monkey. Scale bar: 50 μm. **(D)** Quantitative analysis of the number of TH positive neurons. Representative results of at least three technical replicates are presented.

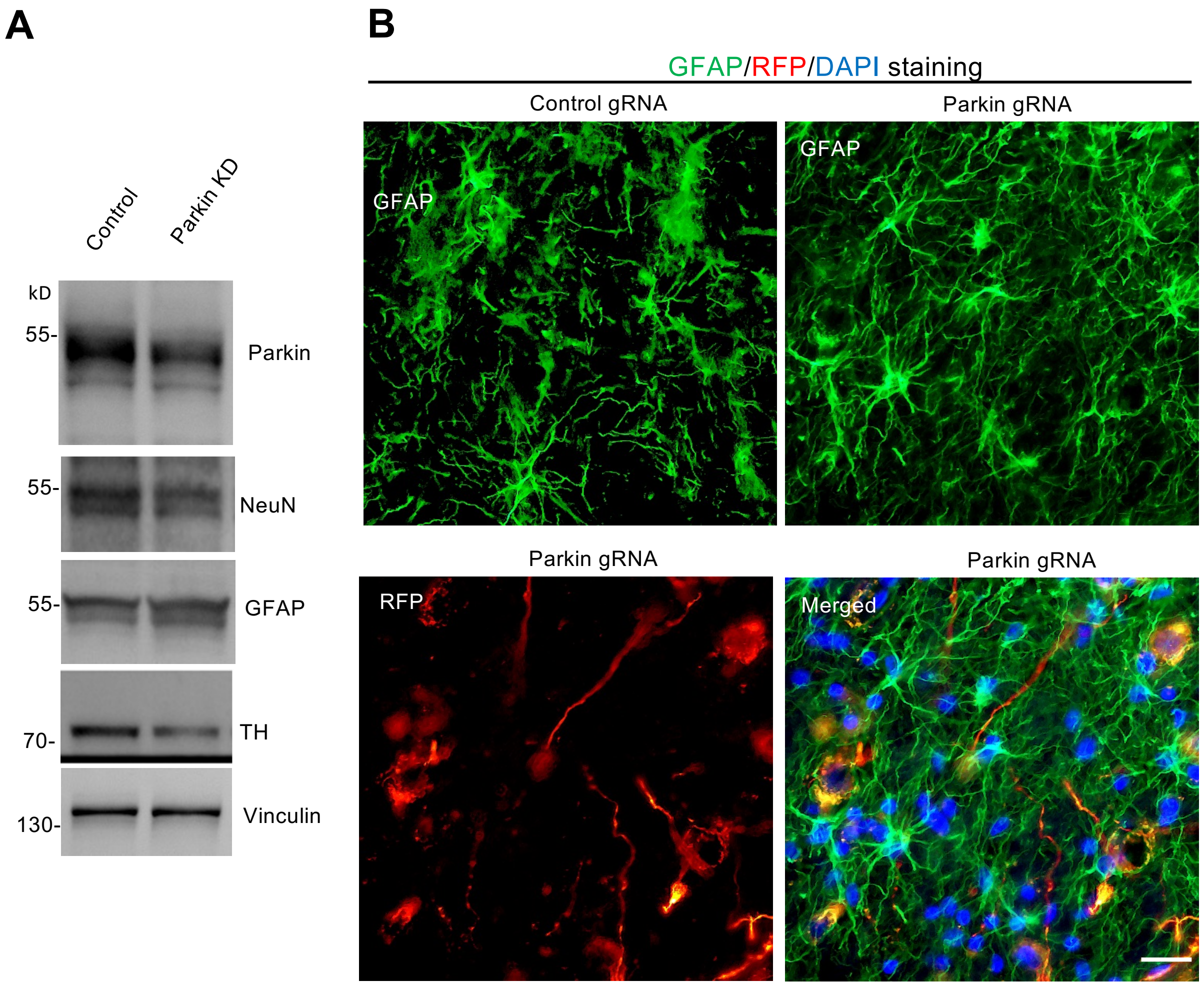

Supplemental Figure 7. *PARK2* targeting does not reduce astrocytes in the monkey brain cortex at 9 years of age. (A) Western blotting showing that targeting *PARK2* via AAV injection reduced Parkin and neuronal protein proteins (NeuN and TH) accompanied by a slight increase in GFAP. **(B)** GFAP immunostaining revealing that the astrocyte density and morphology in AAV Parkin gRNA/Cas9 are similar to those in the control gRNA-injected region. RFP reflects AAV-Parkin gRNA expression. Scale bar: 25 µm. Representative results of at least three technical replicates are presented.

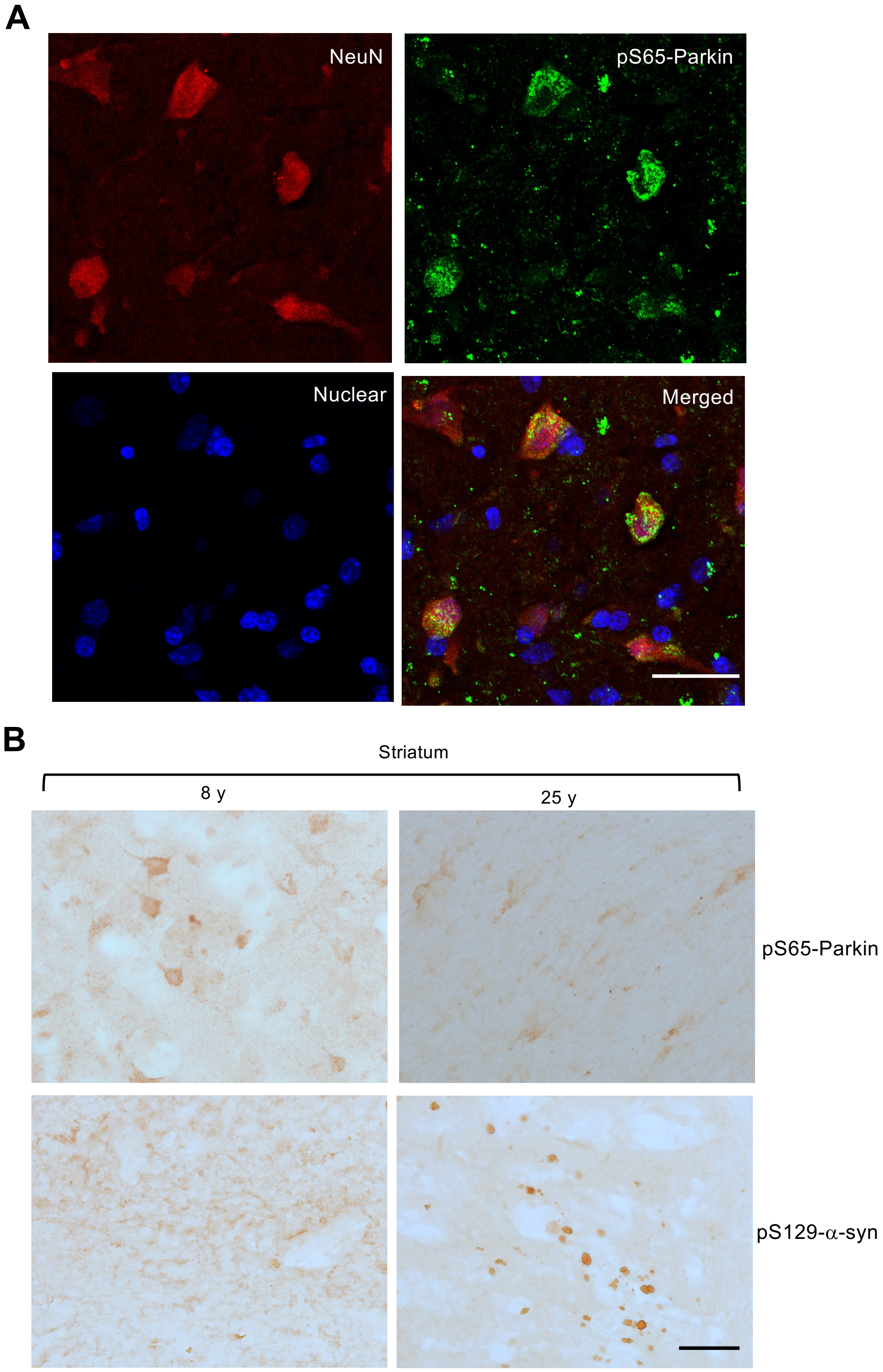

Supplemental Figure 8. Expression of pS65-Parkin in the monkey striatum. (A) Double immunofluorescent staining showing that neuronal cells in the monkey striatum express pS65-Parkin. **(B)** Immunohistochemistry showing the reduced expression of S65-Parkin and increased level of pS129-α-syn in the striatum of 25-year-old monkey as compared with 8-year-old monkey. Scale bar: 25 μm. Representative results of at least three technical replicates are presented.

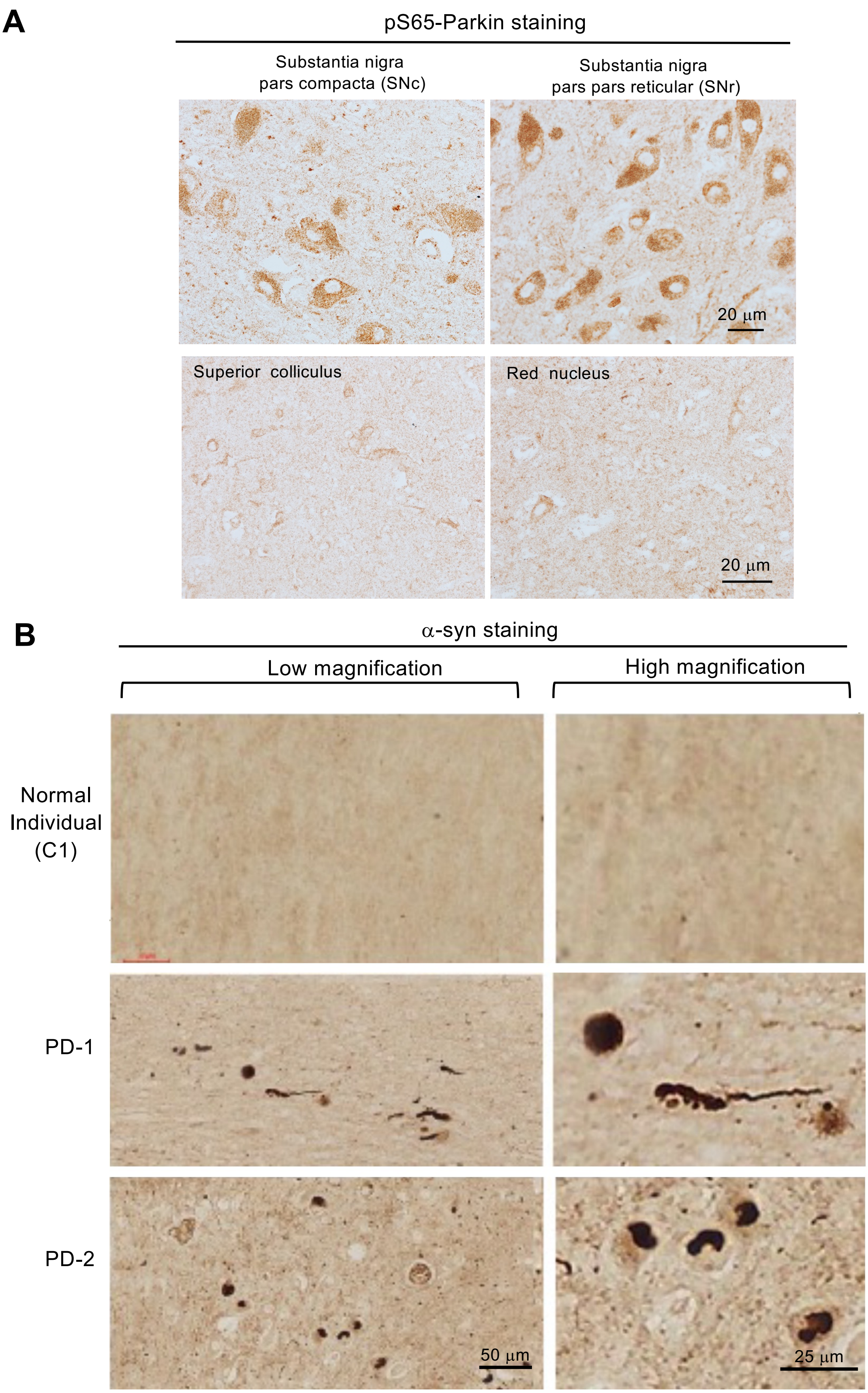

Supplemental Figure 9. The presence of Lewy bodies in the brains of sporadic PD patients. (A) Differential expression of pS65-Parkin in the normal human brain. **(B)** The presence of Lewy bodies in the SN of PD patient brains (PD-1 and PD-2) that were used for examining pS65-Parkin. C1 is the SN from a normal individual. Representative results of at least three technical replicates are presented.
