# Supplementary material for: Deficiency of parkin causes neurodegeneration and accumulation of pathological α-synuclein in monkey models
Source: J Clin Invest. 2024 Oct 15;134(20):e179633. doi: 10.1172/JCI179633 (PMC11473153; doi:10.1172/JCI179633)

Full unedited blot/gel

We normally used the same samples to run western blotting multiple times in independent experiments. Western blotting can analyze multiple proteins on the same blot for quantification and rigorous evaluation of the relative levels of detected proteins. Therefore, it is common practice to section the blot into strips containing proteins of varying molecular weights, each probed with different antibodies. A blot was also stripped to allow for additional probing with different antibodies. This method not only conserves precious samples obtained from small brain regions or limited non-human primate tissues but also enhances the efficiency of analysis.

When repeating the same samples using different gels, we typically also probed the same blot with antibodies for housekeeping gene products (such as vinculin, GAPDH, and others) to ensure an unbiased comparison of results from different gels under consistent conditions. For some proteins that do not exhibit changes in Parkin or PINK1 knocked down tissues, we also incorporated loading controls in the unedited blots. However, we specified that certain loading controls were omitted from the main figures due to space constraints or the presence of other protein bands that could serve as loading controls.

The following unedited blot/gel images are for the representative results in the manuscript.

Fig 1C

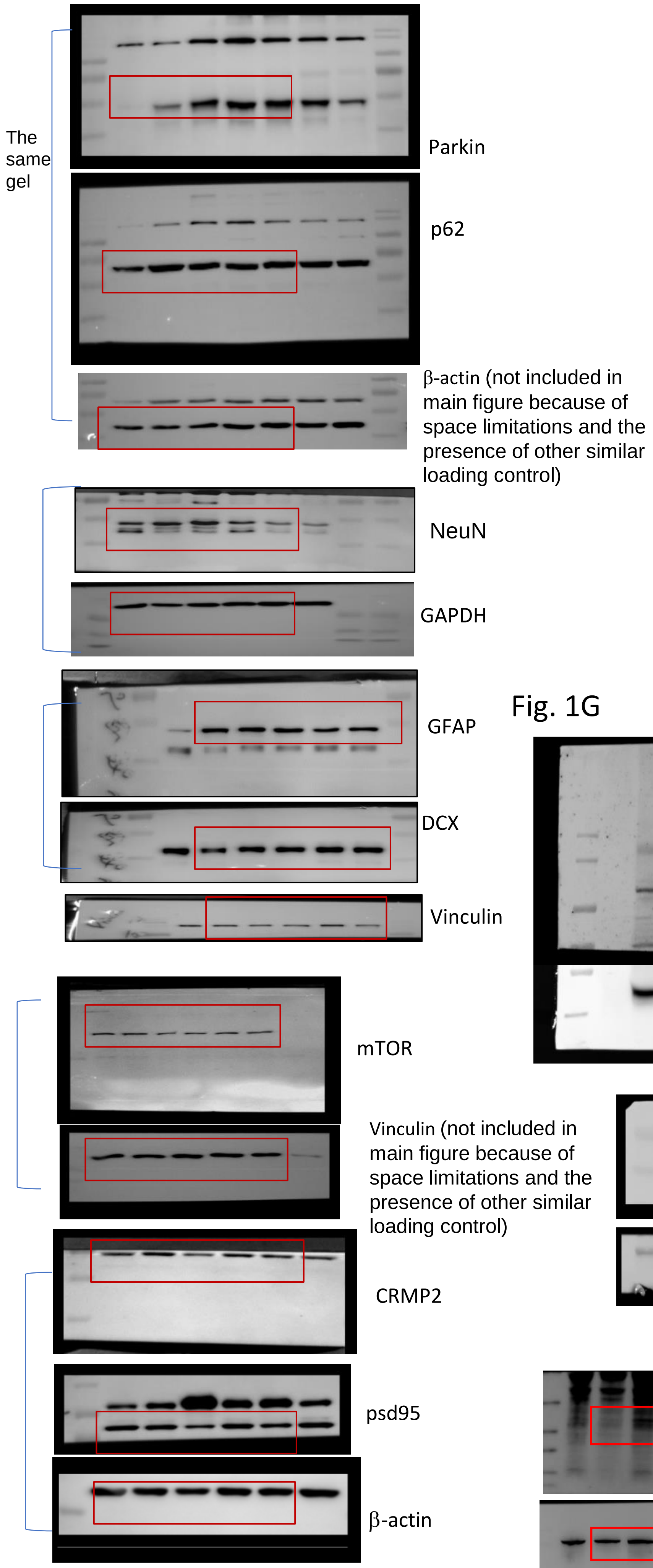

Fig 1D

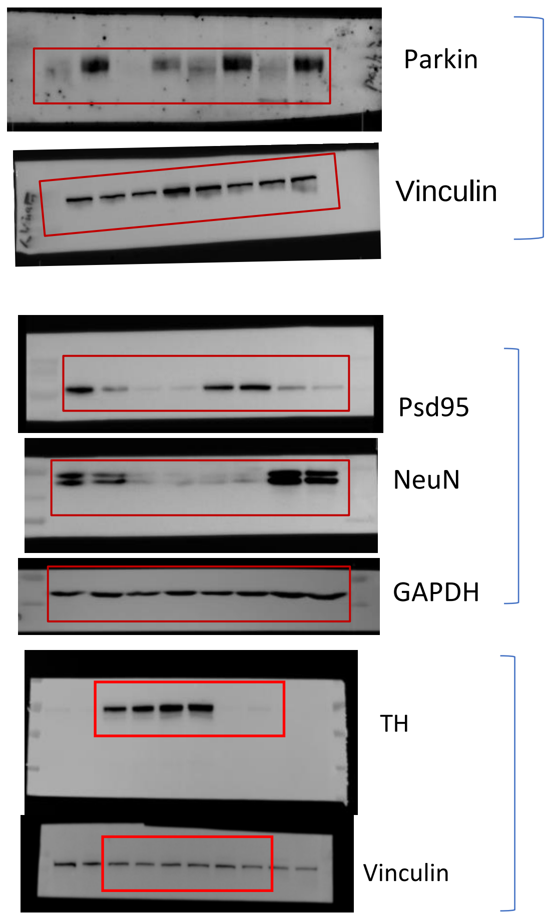

Fig. 1G

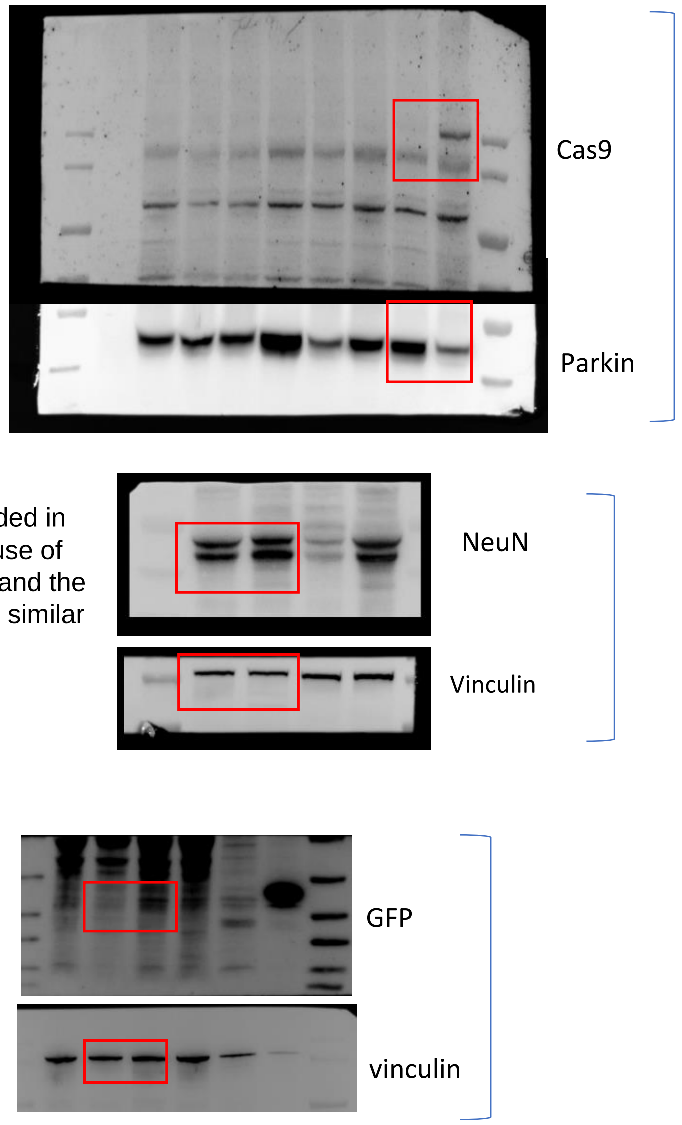

Fig 3C

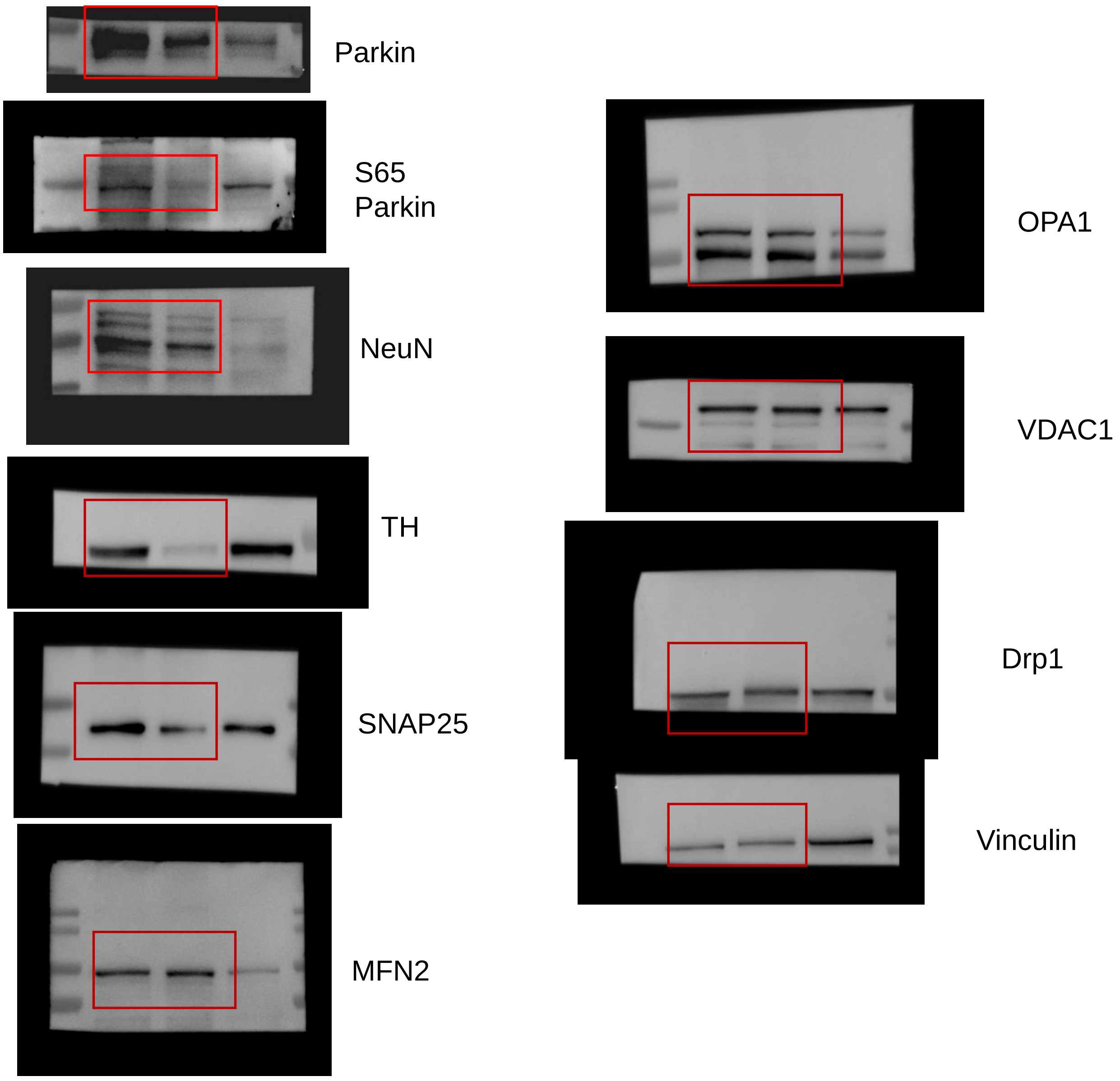
Parkin
S65 Parkin
NeuN
TH
SNAP25
MFN2
OPA1
VDAC1
Drp1
Vinculin

Fig. 4B

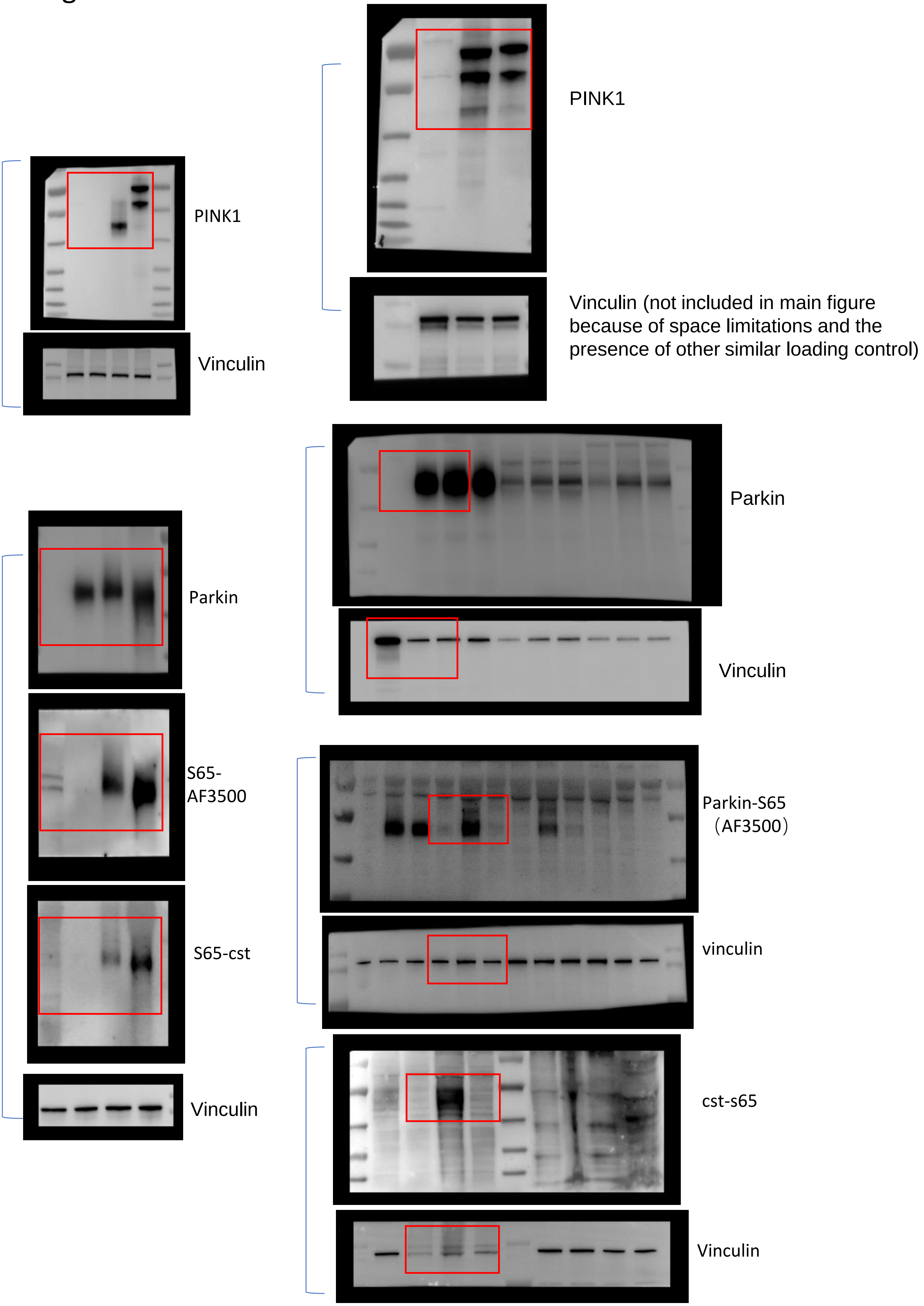
PINK1
Vinculin
PINK1
Vinculin (not included in main figure because of space limitations and the presence of other similar loading control)
Parkin
S65-
AF3500
S65-cst
Vinculin
Parkin
Vinculin
Parkin-S65
（AF3500）
vinculin
cst-s65
Vinculin

Fig. 4C

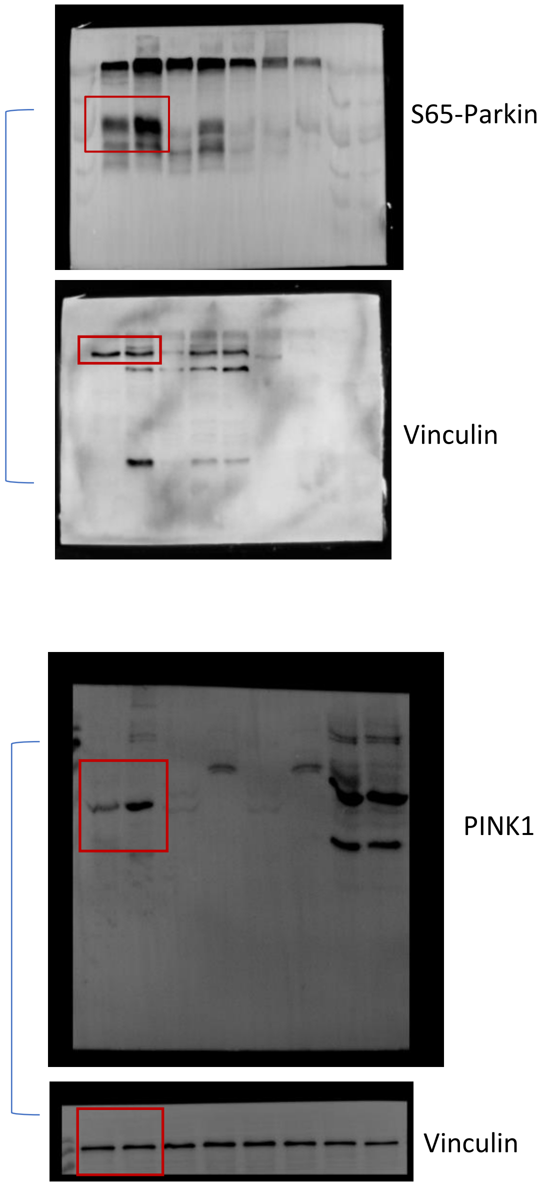
S65-Parkin
Vinculin
PINK1
Vinculin

Fig. 4D

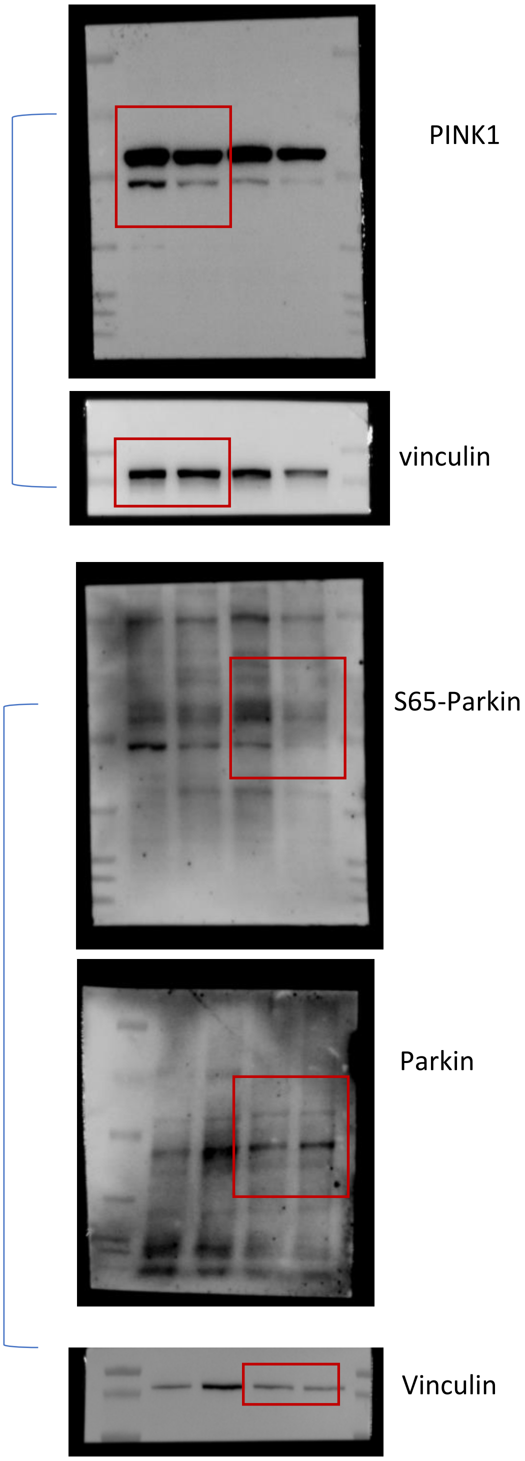
PINK1
vinculin
S65-Parkin
Parkin
Vinculin

Fig. 4E

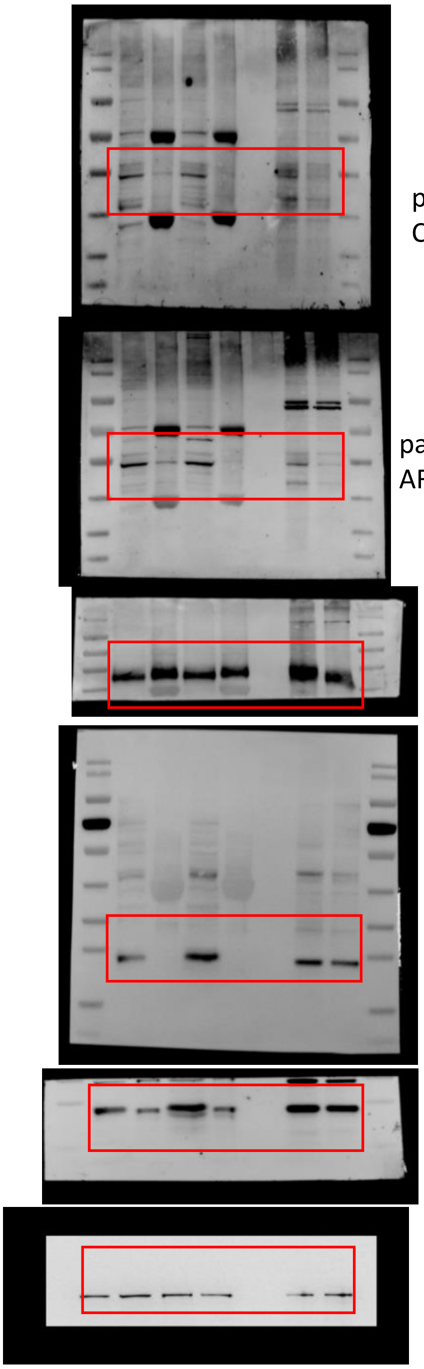

parkin S65
CST

parkin S65
AF3500

Parkin

BAD
S112

Total
BAD

Vinculin

Figure 4F

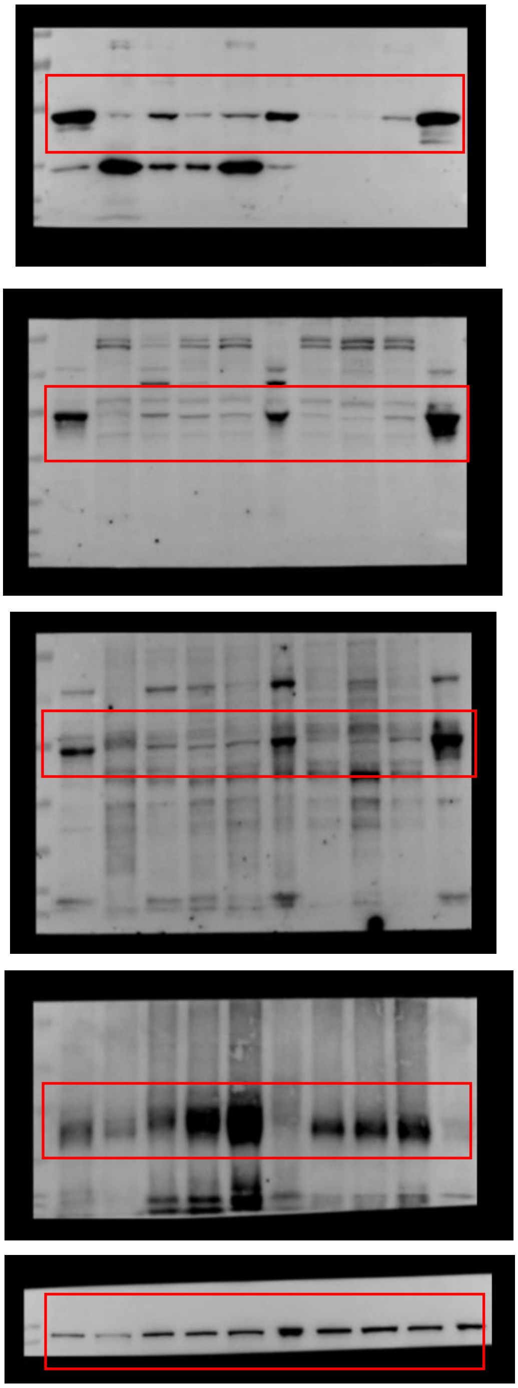

PINK1

pS65-
Parkin

pS65-
Parkin

Parkin

Vinculin

Fig 4G

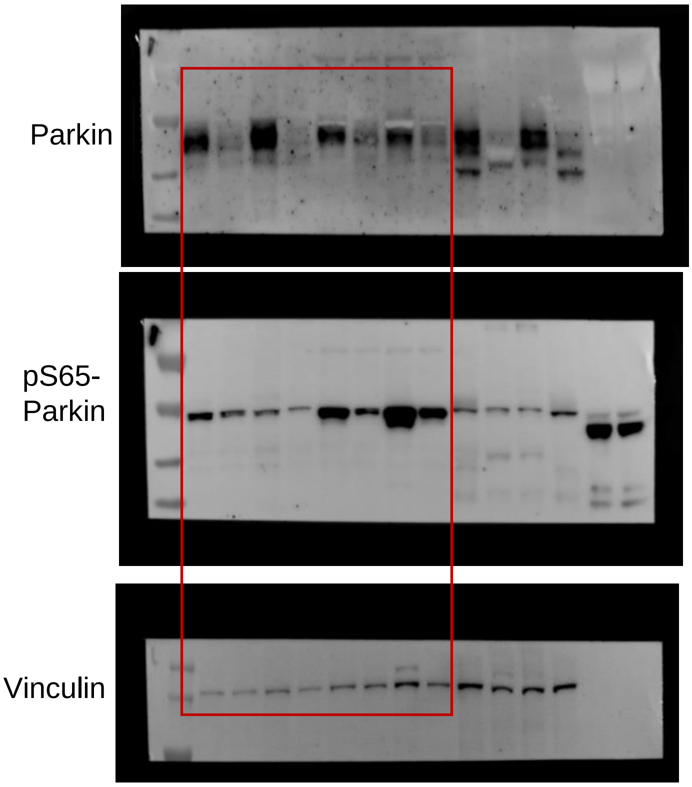
Parkin
pS65-
Parkin
Vinculin

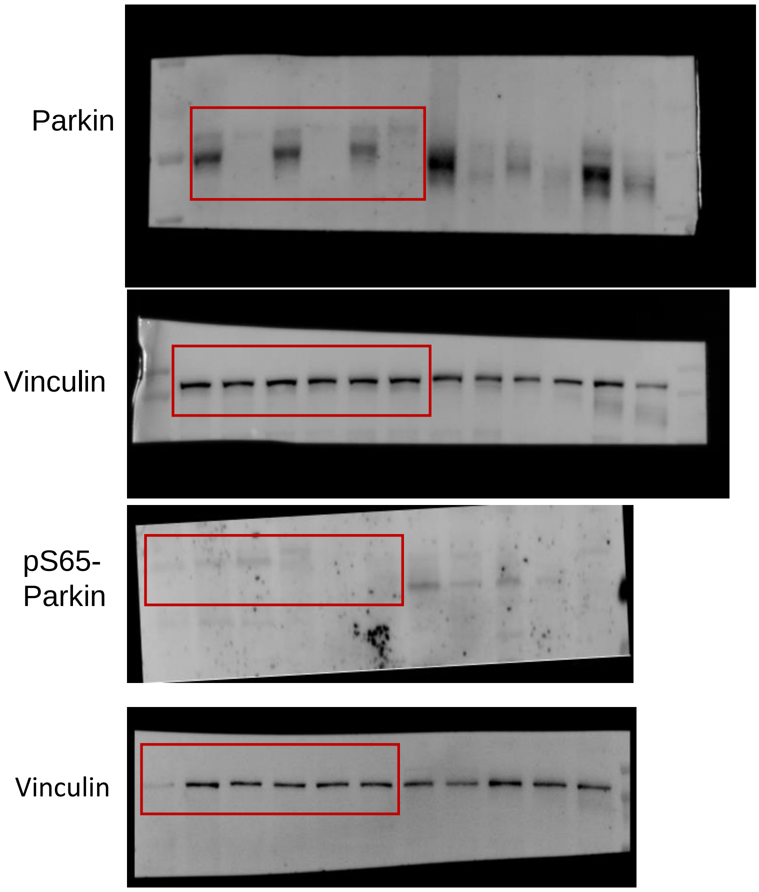
Parkin
Vinculin
pS65-
Parkin
Vinculin

Fig. 5C

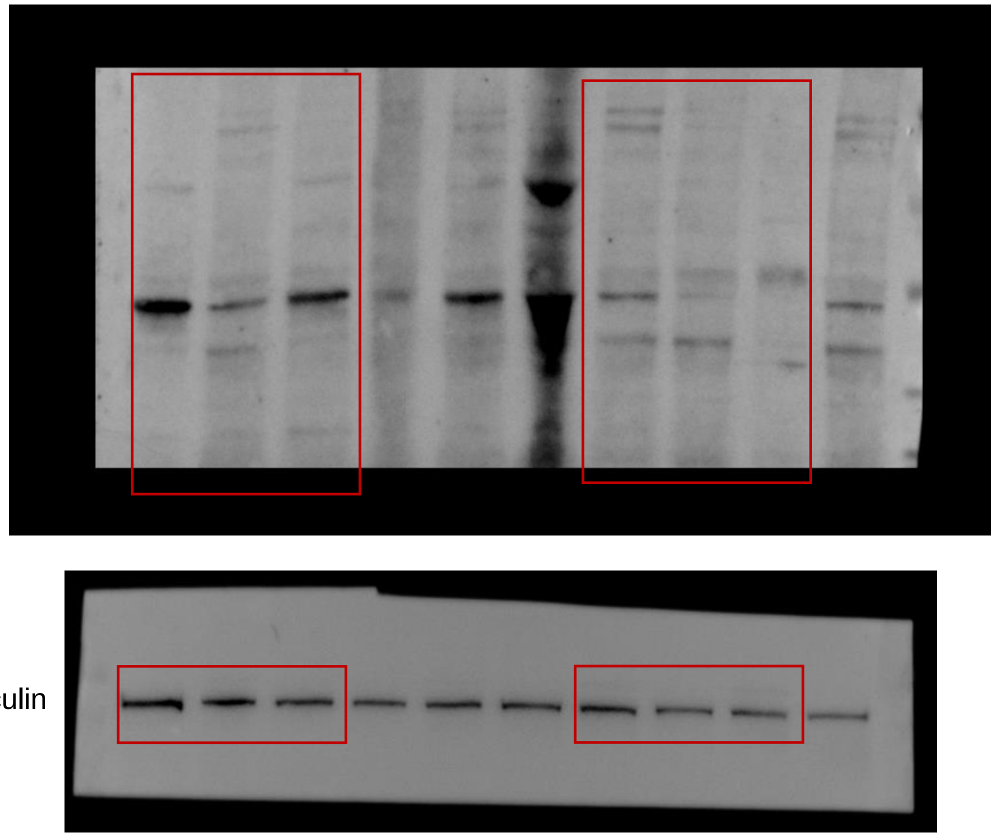

pS65-Parkin

Vinculin

Fig 5D

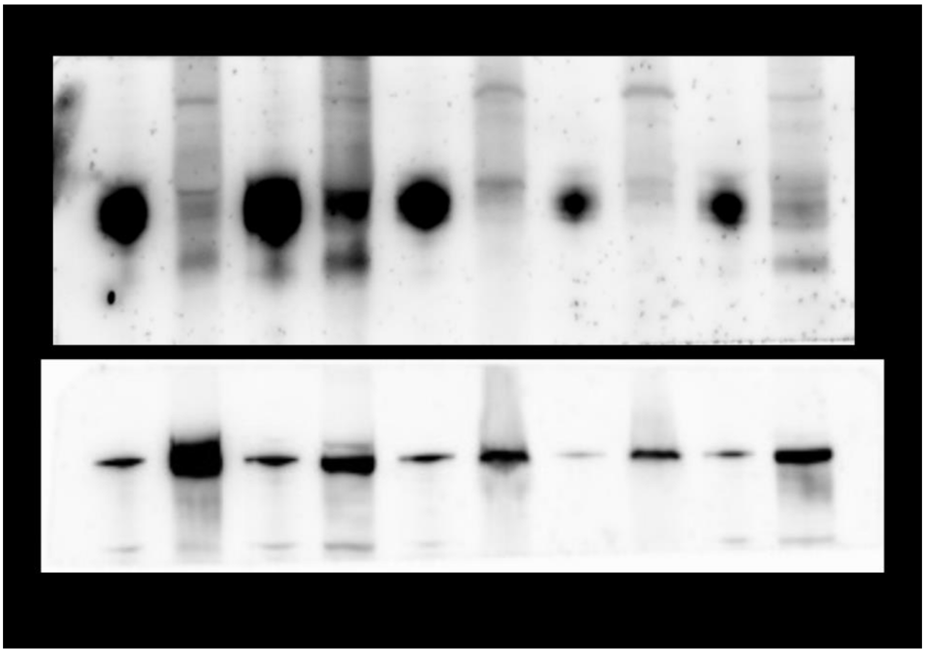

Parkin

Vinculin

Fig. 5E

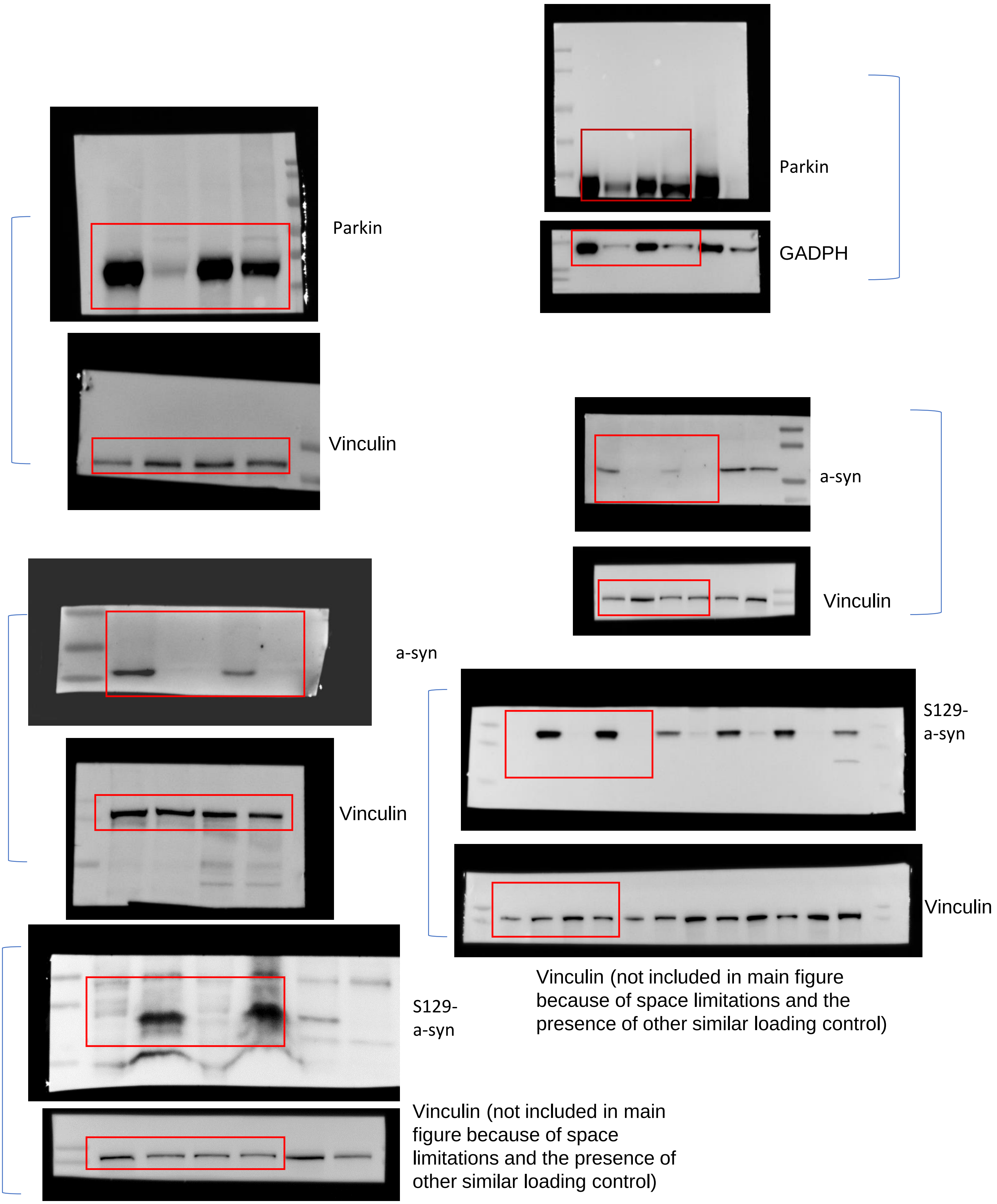
Parkin
Parkin
GADPH
Vinculin
a-syn
Vinculin
a-syn
S129-
a-syn
Vinculin
Vinculin
Vinculin (not included in main figure because of space limitations and the presence of other similar loading control)
S129-
a-syn
Vinculin (not included in main figure because of space limitations and the presence of other similar loading control)

Fig. 6A

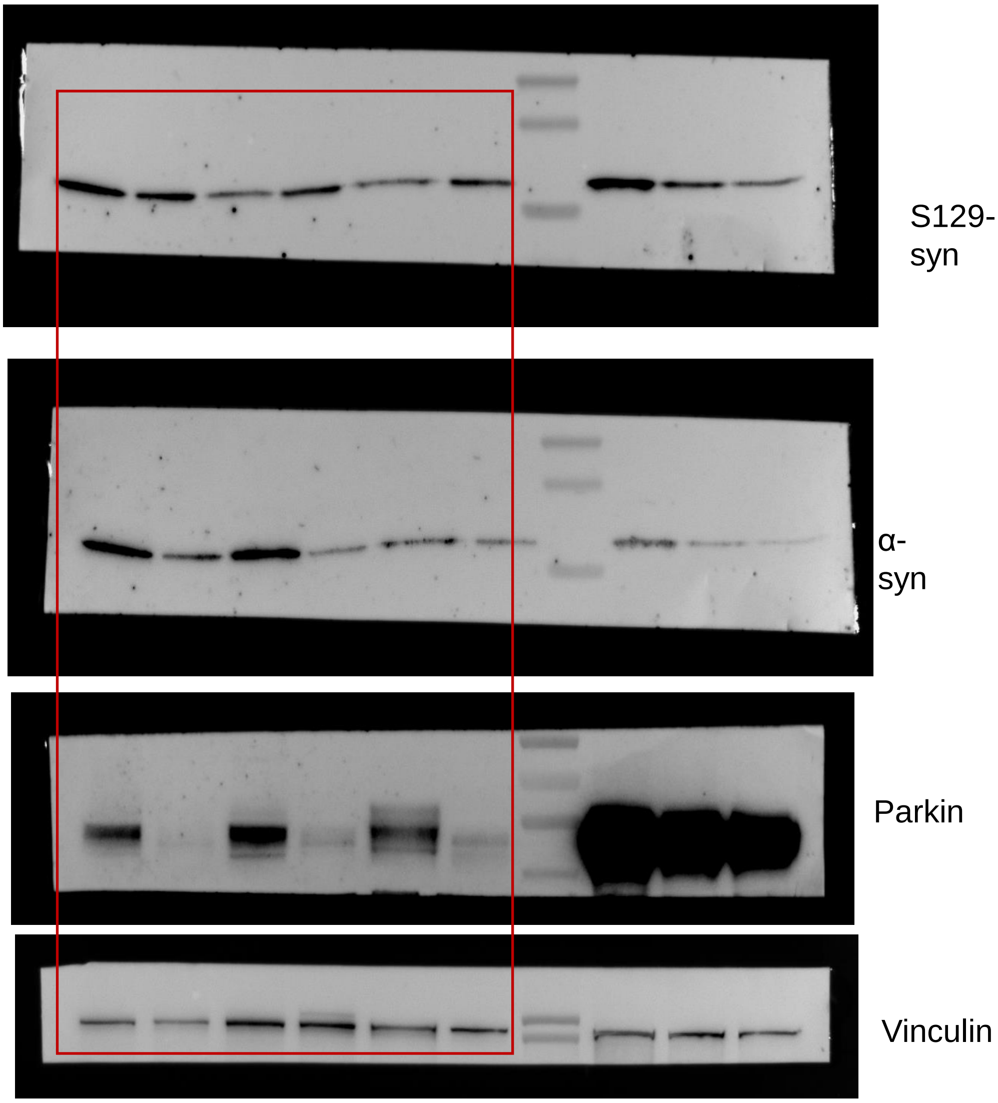
S129-
syn
α-
syn
Parkin
Vinculin

Fig. 6B

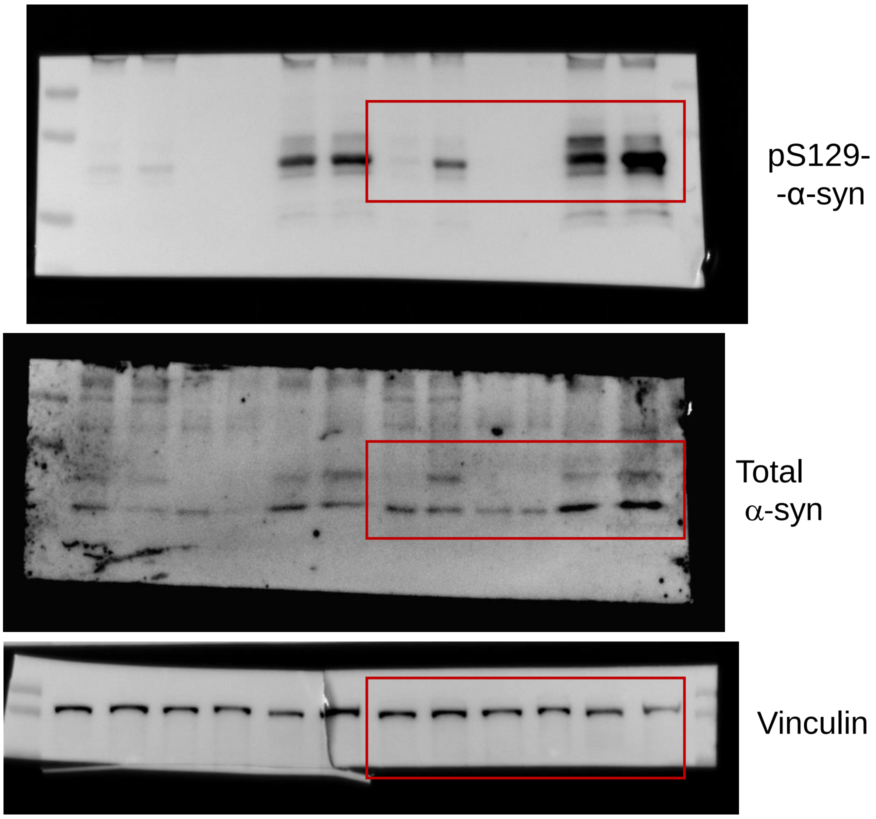
pS129-
-α-syn
Total
α-syn
Vinculin

Fig. 7D

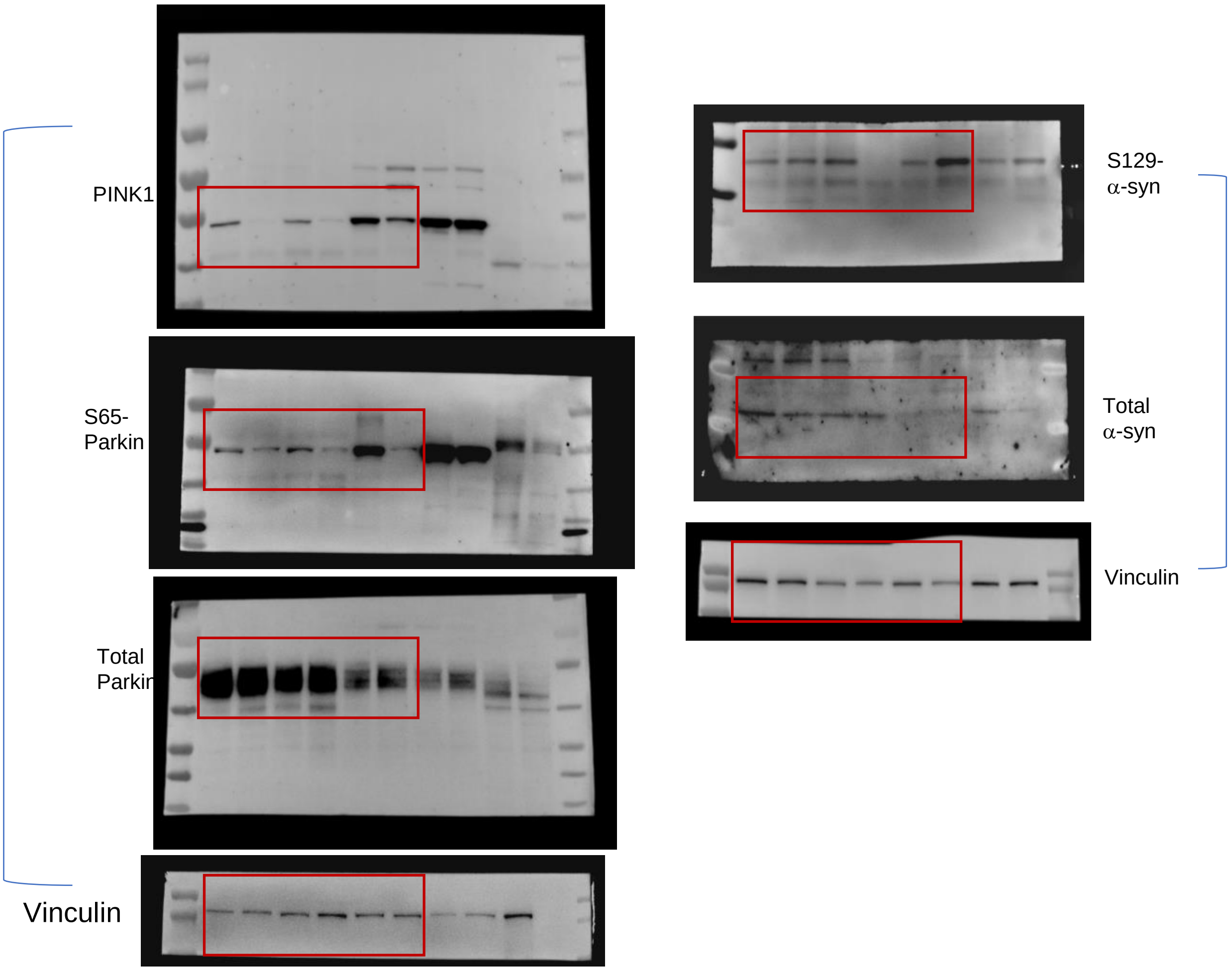
PINK1
S65-
Parkin
Total
Parkin
Vinculin
S129-
α-syn
Total
α-syn
Vinculin

Fig. 8B

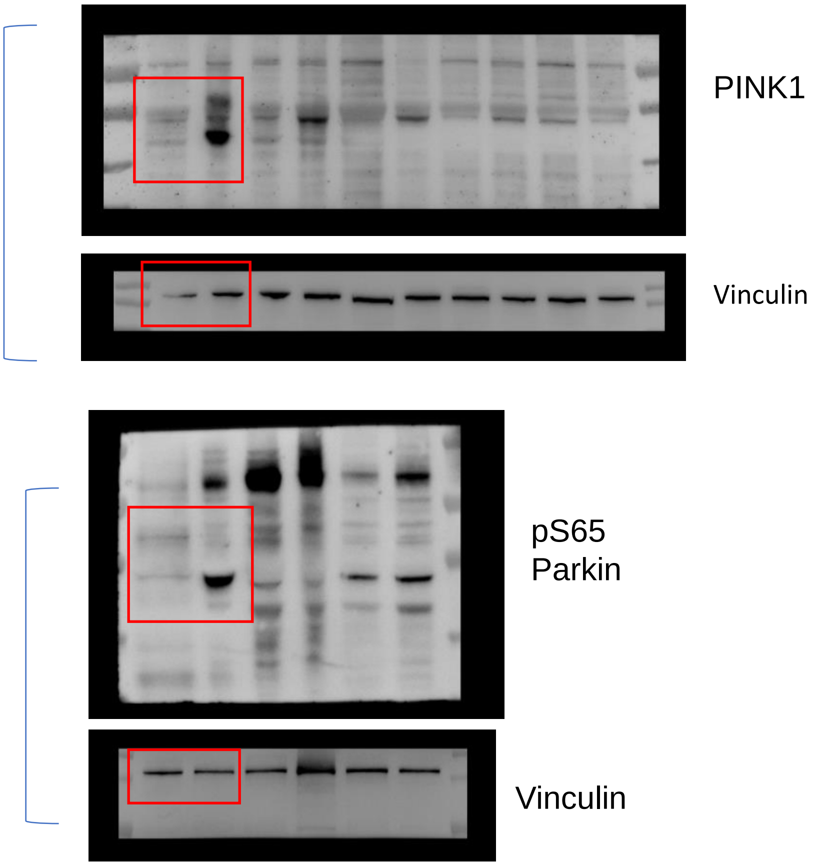
PINK1
Vinculin
pS65
Parkin
Vinculin

Fig. 8E

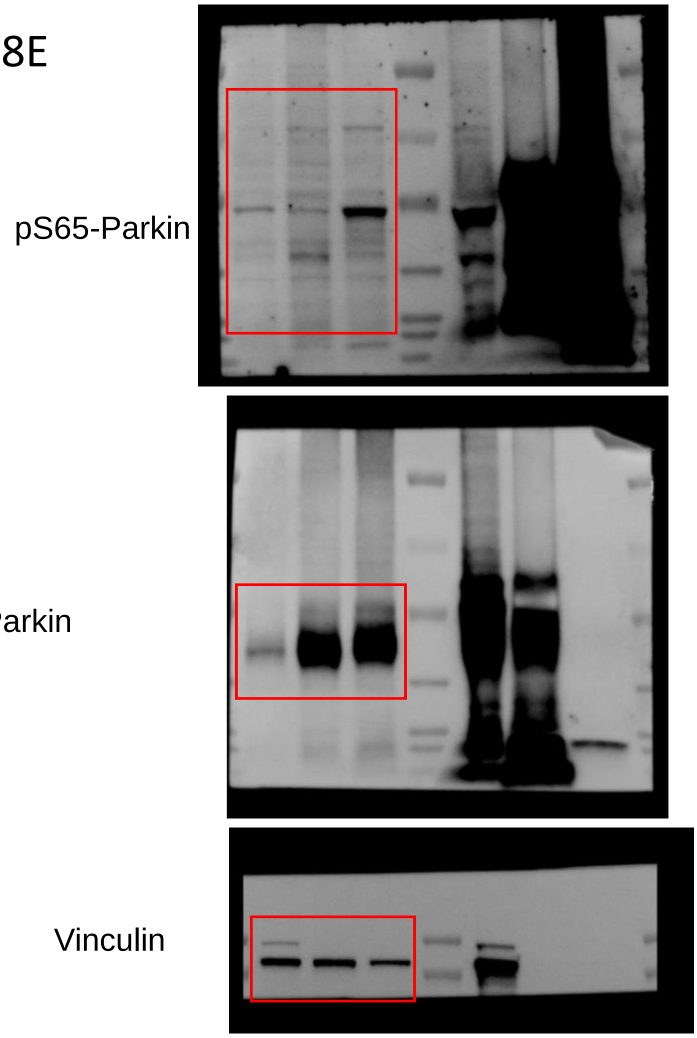
pS65-Parkin
Parkin
Vinculin

Supplemental Figure 1

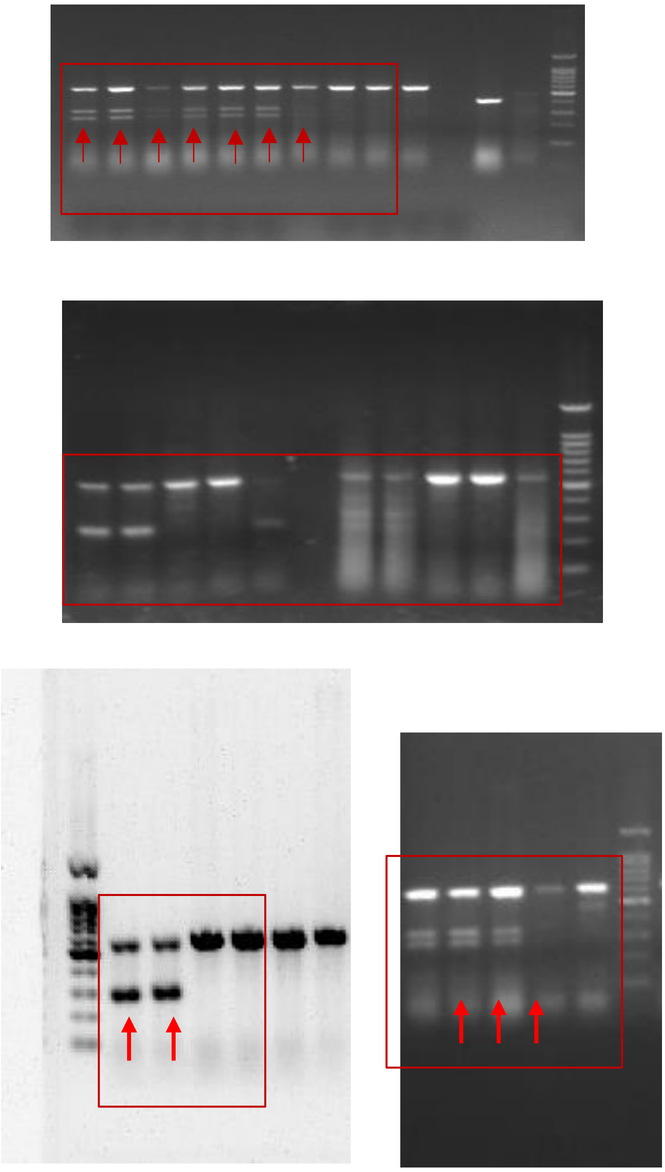

Sup Fig 3

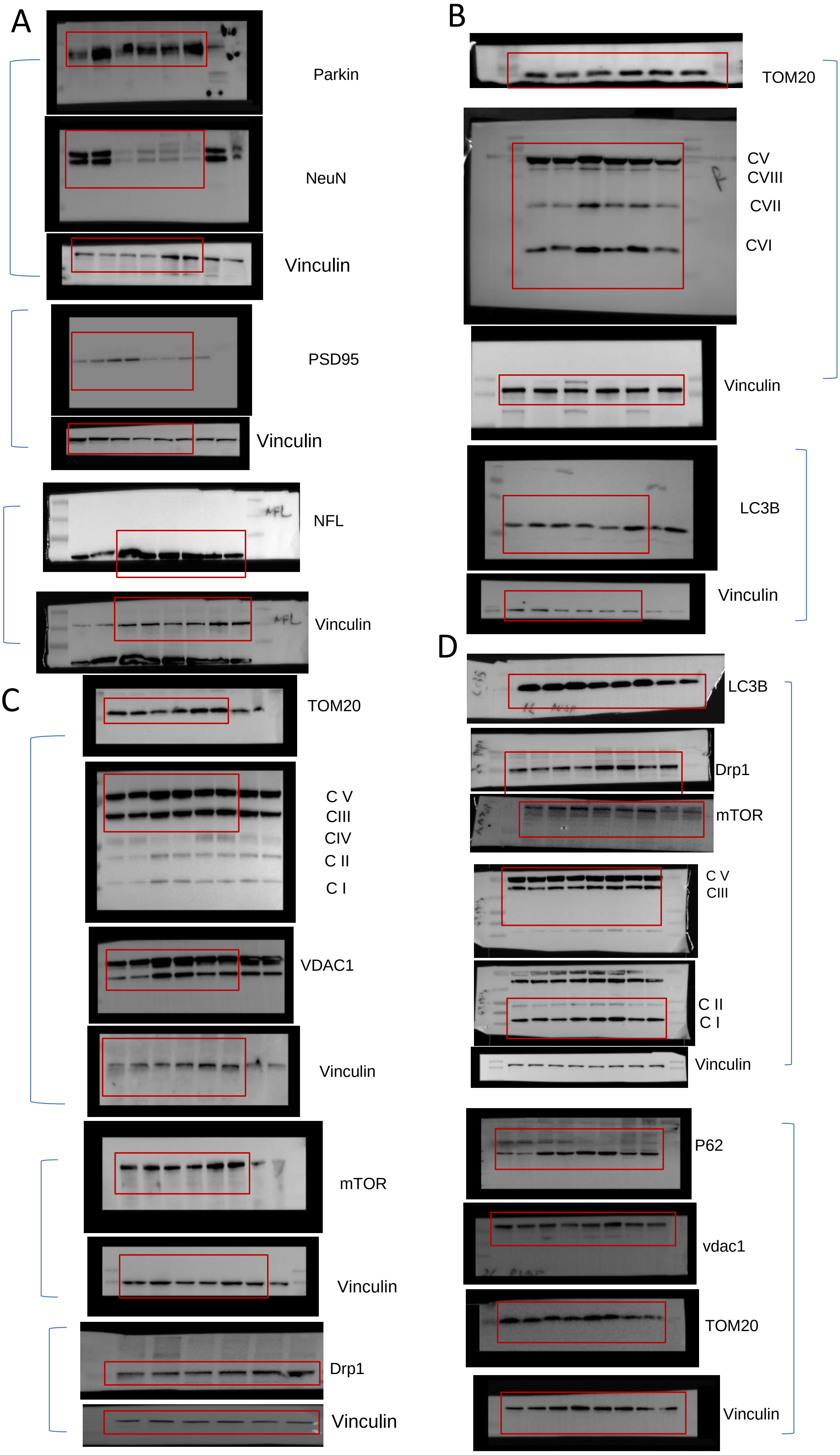
A
Parkin
NeuN
Vinculin
PSD95
Vinculin
NFL
Vinculin
B
TOM20
CV
CVIII
CVII
CVI
Vinculin
LC3B
Vinculin
C
TOM20
C V
CIII
CIV
C II
C I
VDAC1
Vinculin
mTOR
Vinculin
Drp1
Vinculin
D
LC3B
Drp1
mTOR
C V
CIII
C II
C I
Vinculin
P62
vdac1
TOM20
Vinculin

Sup Fig 5B

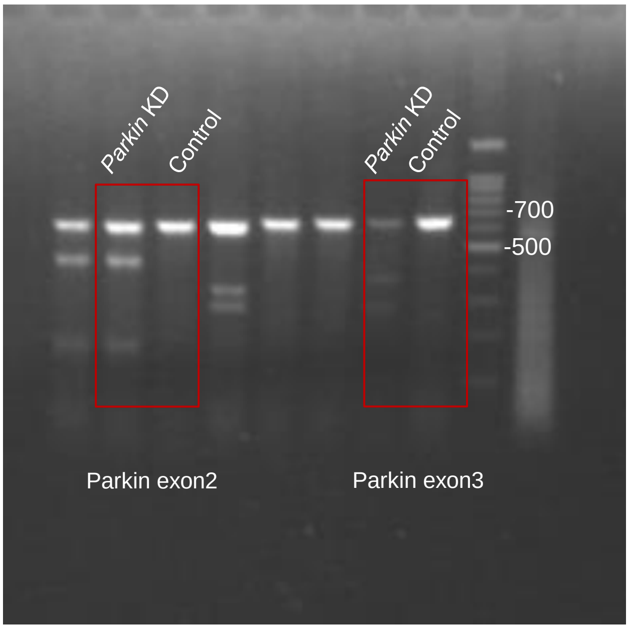
Parkin KD
Control
Parkin KD
Control
-700
-500
Parkin exon2
Parkin exon3

Suppl Fig 7A

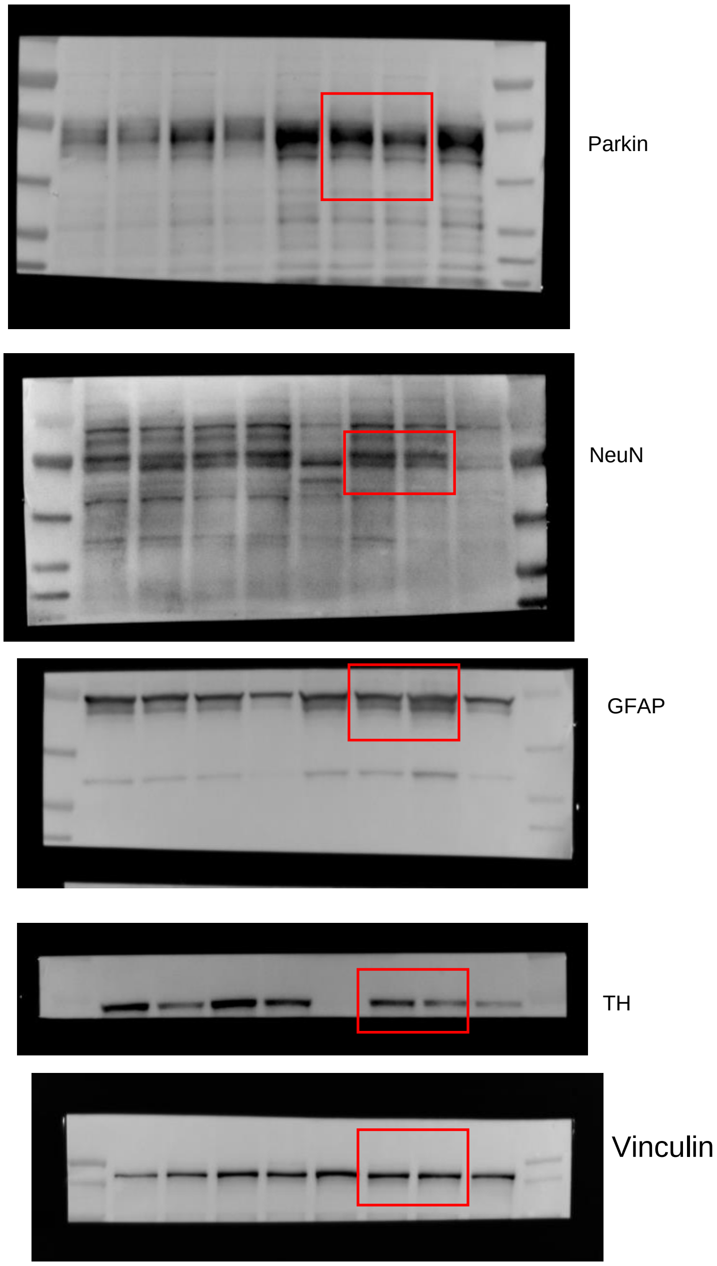

Supplement: Unedited blot and gel images [file jci-134-179633-s079.pdf]
